# Incorporation of Novel Synthetic Glycolipids in Liposomal Nanoparticles Affects Opsonization and In Vivo Clearance

**DOI:** 10.1002/anie.202520837

**Published:** 2026-04-22

**Authors:** Yingjie Yu, Xuehan Li, Yu Gao, Shijia Tao, Xiaofei Li, Lemei Zhao, Wenshuai Han, Hao Fan, Ying Qiu, Man Wang, Luying Zhou, Xiaoyan Fang, Wenhua Yang, Haiyang Zhang, Volker Mailänder, Daniel Crespy, Katharina Landfester, Shuai Jiang, Xiangzhao Mao

**Affiliations:** ^1^ State Key Laboratory of Marine Food Processing and Safety Control Key Laboratory of Marine Drugs Chinese Ministry of Education Ocean University of China Qingdao China; ^2^ School of Medicine and Pharmacy Ocean University of China Qingdao China; ^3^ Laboratory For Marine Drugs and Bioproducts Qingdao Marine Science and Technology Center Qingdao China; ^4^ College of Food Science and Engineering Ocean University of China Qingdao China; ^5^ Department of Dermatology University Medical Center of the Johannes Gutenberg‐University Mainz Germany; ^6^ Department of Materials Science and Engineering School of Molecular Science and Engineering Vidyasirimedhi Institute of Science and Technology VISTEC Rayong Thailand; ^7^ Max Planck Institute For Polymer Research Mainz Germany

**Keywords:** biosynthesis, drug delivery, nano–bio interactions, nanomedicine, saccharide

## Abstract

Mimicking cell membrane glycocalyx, saccharide modification of nanoparticles offers a potent means to regulate their in vivo fate. Here, we investigate how glycosylation (i.e., glucose, galactose, fructose, mannose, and *N*‐acetylglucosamine) regulates liposomal nanoparticle interactions with plasma proteins and immune cells, which further determine their biodistribution and therapeutic efficacy. While fructose conferred the greatest enhancement in tumor cell uptake in vitro, *N*‐acetylglucosamine‐modified nanoparticles achieved the highest tumor accumulation and markedly attenuated systemic clearance in vivo, highlighting a pronounced disparity between in vitro and in vivo performance. The compromised in vivo efficacy of glycosylated nanoparticles was linked to significant clearance in blood, liver, and spleen, primarily mediated by blood monocytes, hepatic stellate cells, and splenic macrophages. Proteomics revealed that adsorption of immunoglobulin G (IgG) and complement C3 facilitates in vivo clearance of nanoparticles. Moreover, IgG deposition further promotes subsequent C3 binding. Notably, *N*‐acetylglucosamine markedly mitigates IgG and C3 adsorption, leading to prolonged circulation and enhanced tumor accumulation and inhibition. Benefiting from glycosylation‐regulated protein corona, doxorubicin‐loaded *N*‐acetylglucosamine‐modified liposomal nanoparticles achieved superior antitumor efficacy compared with other glycosylated formulations. This study establishes a clear correlation between glycosyl ligand identity, protein corona composition, and in vivo performance, providing fundamental insights for rational design and clinical translation of glycosylated nanomedicines.

## Graphical abstract

We prepared five glycosylated liposomal nanoparticles (G‐LNPs) to investigate the role of glycosylation and protein corona in modulating the in vivo behavior of G‐LNPs. We show that IgG and complement C3 adsorption enhanced liposomal nanoparticle clearance, with IgG promoting subsequent C3 binding. This study establishes a clear correlation between glycosyl ligands, protein corona composition, and biological fate of G‐LNPs.

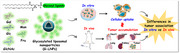

## 1 Introduction

The glycocalyx, a dense layer of glycoproteins and glycolipids on cell membranes, plays key roles in cell communication, adhesion, and homeostasis [1, 2, 3, 4]. Inspired by natural glycocalyx functions, surface glycosylation of nanomedicines has emerged as a promising strategy to enhance drug delivery by exploiting carbohydrate–receptor interactions [5, 6, 7]. For example, galactose and glucose can bind to highly expressed asialoglycoprotein receptor (ASGPR) in hepatocytes and hepatocellular carcinoma cells [8], while mannose and galactose exhibit strong affinity for lectin‐like receptors overexpressed on tumor cells [9]. Mannose also promotes dendritic cell uptake via mannose receptors, enhancing nanovaccine uptake and antigen presentation efficiency [10, 11]. Moreover, glucose recognition by glucose transporter subtype I enables drug delivery to the brain via crossing the blood‐brain barrier [12]. Despite their promising potentials, the facts that no actively targeted nanomedicines have successfully passed clinical trials and only a median of 0.7% of systemically administered nanoparticle doses reach tumor tissues [13, 14], underscore the need to better understand the in vivo fate and biological interactions of glycosylated nanomedicines.

Growing evidence indicates that glycosylation plays a critical role in shaping the biological behavior of nanomedicines. For instance, glucose‐modified liposomes show enhanced tumor accumulation and retention but faster blood clearance [15], whereas gangliosides [16, 17] and galactose modification [18] can prolong circulation and improve bioavailability. Adjusting the amino/hydroxyl ratios of glycosylated polymers on nanovesicles extends circulation time and enhances antitumor efficacy [7]. Moreover, glycocalyx‐mimicking nanoparticles bearing multiple glycosyl ligands enabled organ‐specific delivery, with defined sugar combinations directing liver, spleen, or kidney targeting [6]. However, most studies focus on empirical targeting outcomes, while the mechanistic basis by which glycosyl ligands govern nano–bio interactions and organ selectivity remains unclear. Moreover, current glycosylation strategies primarily rely on post‐modifications, which lack precise control over glycosylation density, thus limiting quantitative comparisons among different saccharide‐functionalized nanomedicines [19].

Upon entry into biological fluids, nanoparticles rapidly adsorb biomacromolecules to form a protein corona, which reshapes their physicochemical properties and imparts a new biological identity [20, 21, 22, 23]. The corona has been shown to reduce in vivo targeting efficiency by obstructing ligand–receptor interactions [24, 25, 26] and redirect nanoparticles toward clearance by the reticuloendothelial, mononuclear phagocytosis, and immune systems [27, 28]. In particular, opsonins such as immunoglobulins and complement proteins promote phagocytic recognition and clearance [29, 30], which complement activation may trigger inflammatory responses [31]. Apolipoproteins enriched on PEG‐modified nanoparticles can prolong blood circulation [32, 33], whereas anti‐PEG antibodies may induce accelerated blood clearance, posing a dilemma in drug delivery [34, 35]. Indeed, protein corona composition is predominantly influenced by nanoparticle physiochemical properties, such as material, size, shape, elasticity, and especially surface characteristics like topology, charge, and hydrophobicity [31, 32, 33, 34, 35, 36]. Notably, glycosylation has been reported to alter surface properties of nanoparticles, such as charge and hydrophilicity, thereby affecting their interactions with proteins and subsequent biological fate. Therefore, precise synthesis of glycosylated nanomedicines with defined saccharide structures and understanding how glycosylation impacts protein corona fingerprints and in vivo fate of nanomedicines is crucial for advancing clinical application of glycosylated nanomedicines.

**SCHEME 1:**
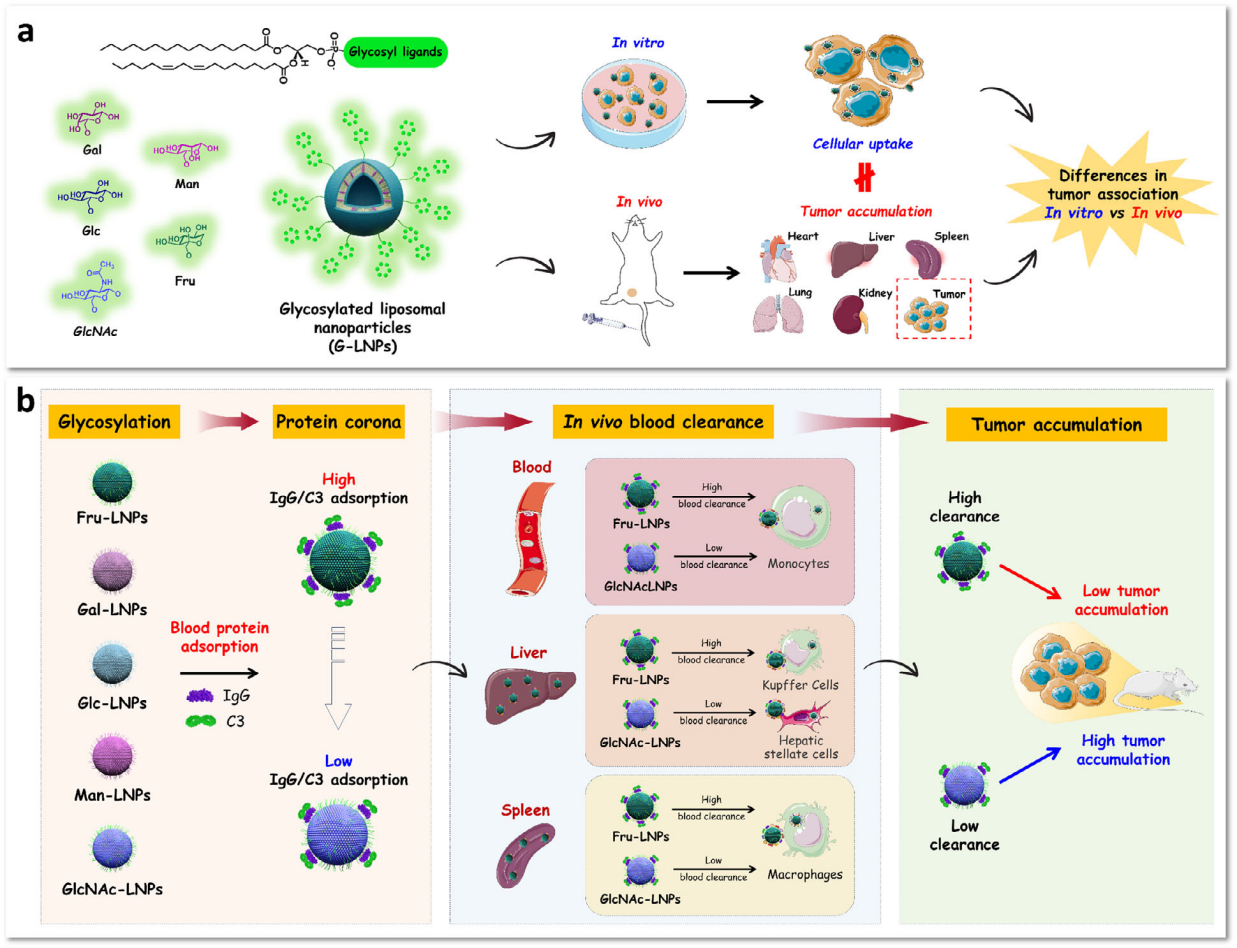
Schematic illustration of the design of glycosylated liposomal nanoparticles (G‐LNPs) containing various glycosyl ligands to explore the protein corona compositions on nanoparticle surfaces and key protein corona components‐affected in vivo clearance and tumor accumulation behaviors. (a) Different G‐LNPs show distinct tumor targeting in vitro and in vivo. (b) Immunoglobulin G/complement C3 specifically modulates the in vivo clearance and tumor accumulation behavior of G‐LNPs.

Here, we investigated how glycosylation regulates in vivo behavior of liposomal nanoparticles (LNPs) through protein corona modulation (Scheme 1). Glycosylated LNPs (G‐LNPs) were constructed by integrating phosphatidyl saccharides biosynthesized via phospholipase D‐catalyzed transphosphatidylation. We first investigated tumor cell uptake and in vivo biodistribution in tumor‐bearing mice to assess tumor association in vitro and in vivo (Scheme 1a). In view of the rapid blood clearance observed for G‐LNPs, we further dissected the contributions of major hepatic and splenic cell populations to their clearance. To elucidate the role of protein corona in modulating LNP clearance, macrophage uptake studies were conducted using LNPs pre‐incubated with sera from different species (human vs. mouse) and disease states (healthy vs. tumor). Complement activation assays and proteomics were performed to identify key corona proteins associated with clearance. Based on these results, we established correlations between glycosyl ligand types, protein corona composition, and biological behaviors (macrophage and tumor cell uptake, complement activation, circulation kinetics, clearance profiles, and tumor accumulation) (Scheme 1b). Finally, selected glycosyl ligands that conferred prolonged circulation and improved tumor accumulation were applied to doxorubicin‐loaded LNPs and validated in orthotopic 4T1 tumor models. This study defines protein corona remodeling as a central mechanism in regulating the clearance and therapeutic performance of glycosylated nanomedicines, providing guidance for their rational design and clinical translation.

## 2 Results and Discussion

### 2.1 Preparation and Characterization of G‐LNPs

We first synthesized five phosphatidyl saccharides, namely phosphatidyl‐galactose (PtdGal), phosphatidyl‐glucose (PtdGlc), phosphatidyl‐*N*‐acetylglucosamine (PtdGlcNAc), phosphatidyl‐mannose (PtdMan), and phosphatidyl‐fructose (PtdFru), through phospholipase D (PLD)‐catalyzed transphosphatidylation reactions between phosphatidylcholine (PC) and various saccharides. High performance liquid chromatography (HPLC) analysis revealed reaction yield of PtdGal, PtdGlc, PtdGlcNAc, PtdMan, and PtdFru as 96.0 ± 0.3%, 94.6 ± 0.5%, 65.8 ± 3.4%, 90.3 ± 0.8%, and 74.3 ± 2.1%, respectively (Figure S1a, b). Chemical structures of the obtained phosphatidyl saccharides were characterized by ^1^H‐NMR spectroscopy, mass spectrometry (MS), MS/MS, and attenuated total reflectance‐Fourier transform infrared (ATR‐FTIR) spectroscopy.

The ^1^H‐NMR spectra of synthesized phosphatidyl saccharides were shown in Figures S2–S6, where the anomeric proton signals of the sugar moieties appear as characteristic doublets at *δ* 4.5–5.5 ppm. However, due to the limited structural differentiation among the five compounds by ^1^H‐NMR, we subsequently employed mass spectrometry and MS/MS analysis for structural identification based on precise molecular masses and characteristic fragmentation patterns of the phospholipid and glycosidic moieties. MS analysis revealed two major aliphatic chain peaks for the five phosphatidyl saccharides, consistent with the fatty acid composition present in the hydrophobic tails of phosphatidylcholine (PC) used as substrate (Figure S7). Specifically, for PtdGlcNAc, the first characteristic peak M_1_ with an *m/z* of 875.55 comprises *N*‐acetyl‐D‐glucosamine as the hydrophilic head group, and palmitic acid and linoleic acid as hydrophobic tails [16:0, 18:2]. Meanwhile, another characteristic peak M2 with an *m/z* of 899.55 was associated with a hydrophilic head group of *N*‐acetyl‐D‐glucosamine and hydrophobic tails consisting of two linoleic acids [18:2, 18:2]. The other four glycoside donors were isomeric, therefore yielding identical *m/z* values for M_1_ [16:0, 18:2] and M_2_ [18:2, 18:2] at 858.52 and 834.52, respectively. Additionally, characteristic peaks at *m/z* 713 and 737 were consistently detected in the MS/MS spectra of all products (Figure S8). Chemical structure analysis confirmed that the phosphate group was linked to the glycoside donor via the 6‐OH group, resulting in the formation of 6‐phosphatidyl saccharides. This result aligns with the known preferential selectivity of PLD for primary hydroxyl groups. In the ATR‐FTIR spectra (Figure S9), the C─C─N^+^ absorption peak of PC at 967 cm^−1^ disappeared, while a broad peak appeared at 3300 cm^−1^, corresponding to hydrogen bonding stretching vibration between hydroxyl groups of the glucose ring, confirming the formation of phosphatidyl saccharides. For PtdGlcNAc, additional peaks at 1645 cm^−1^ (C═O vibration) and 1555 cm^−1^ (N─H vibration) indicated the incorporation of acetyl amino groups in its structure. These results confirmed the successful enzymatic synthesis of phosphatidyl saccharides.

**FIGURE 1:**
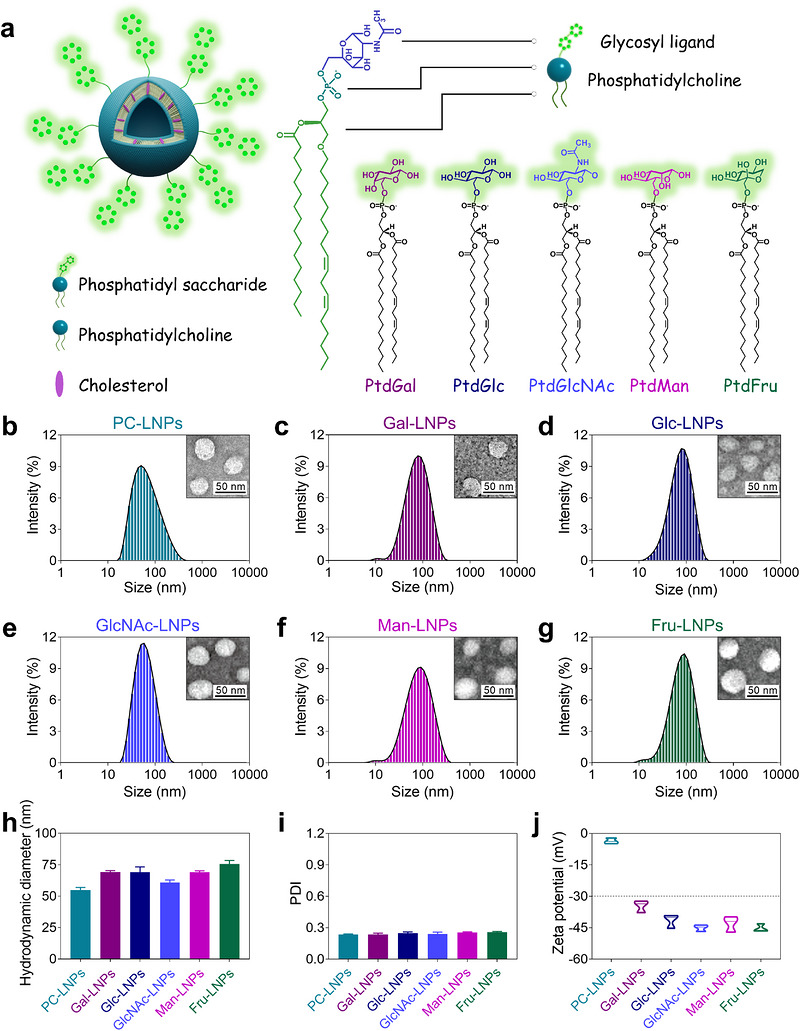
Characterization of glycosylated liposomal nanoparticles (G‐LNPs). (a) Schematic illustration of the composition of G‐LNPs. (b–g) Hydrodynamic diameter distributions of G‐LNPs measured by DLS (insets: corresponding TEM micrographs): PC‐LNPs (b), Gal‐LNPs (c), Glc‐LNPs(d) GlcNAc‐LNPs (e), Man‐LNPs (f), and Fru‐LNPs (g). (h and i) Average hydrodynamic diameters (h) and polydispersity index (PDI) (i) of G‐LNPs. (j) Zeta potentials of G‐LNPs. Data are presented as mean ± SD (*n* = 3).

G‐LNPs were prepared via a thin‐film dispersion‐sonication method by incorporating glycolipids into the lipid bilayers (Figure 1a). To eliminate the influence of surface charge on the biological behavior of G‐LNPs, phosphatidic acid (PA) was incorporated at the same molar percentage (37.5 mol%) as the glycolipids to generate charge‐matched LNPs as a reference. The resulting formulations were denoted as PC‐LNPs, PA‐LNPs, Gal‐LNPs, Glc‐LNPs, GlcNAc‐LNPs, Man‐LNPs, and Fru‐LNPs. As shown in Figure 1b‐I and Tables S1–S3, dynamic light scattering (DLS) analysis revealed that G‐LNPs possessed hydrodynamic diameters in the range of 50–80 nm with a PDI below 0.3. Transmission electron microscopy (TEM) further confirmed the well‐defined spherical morphology of the liposomal nanoparticles (Figures 1b–g and S10). Statistical quantitative analysis of size distributions from TEM images using Nano Measurer software showed particle diameters below 50 nm, which are smaller than the DLS‐derived hydrodynamic sizes, as expected due to sample dehydration during TEM preparation (Figure S11). In addition, incorporation of glycolipids markedly increased the magnitude of negative zeta potential relative to PC‐LNPs (Figure 1j, Table S1). Notably, PA‐LNPs exhibited particle sizes and zeta potentials nearly identical to those of the glycosylated LNPs (Figure S10, Table S1), thereby isolating surface charge as a controlled variable. Accordingly, PA‐LNPs were used as reference control, enabling differences in in vivo clearance and biological behavior to be attributed specifically to glycosylation rather than charge effects. Hemolysis tests showed hemolysis percentages below 5% for all G‐LNPs (Figure S12), while cell viability remained above 80% for HUVECs, L929, and RAW264.7 cells (Figures S13–S15), and blood biochemistry showed no difference at 24 h (Figure S16), confirming the safe character of the G‐LNPs.

### 2.2 Tumor Targeting and Pharmacokinetic Behaviors of G‐LNPs

**FIGURE 2:**
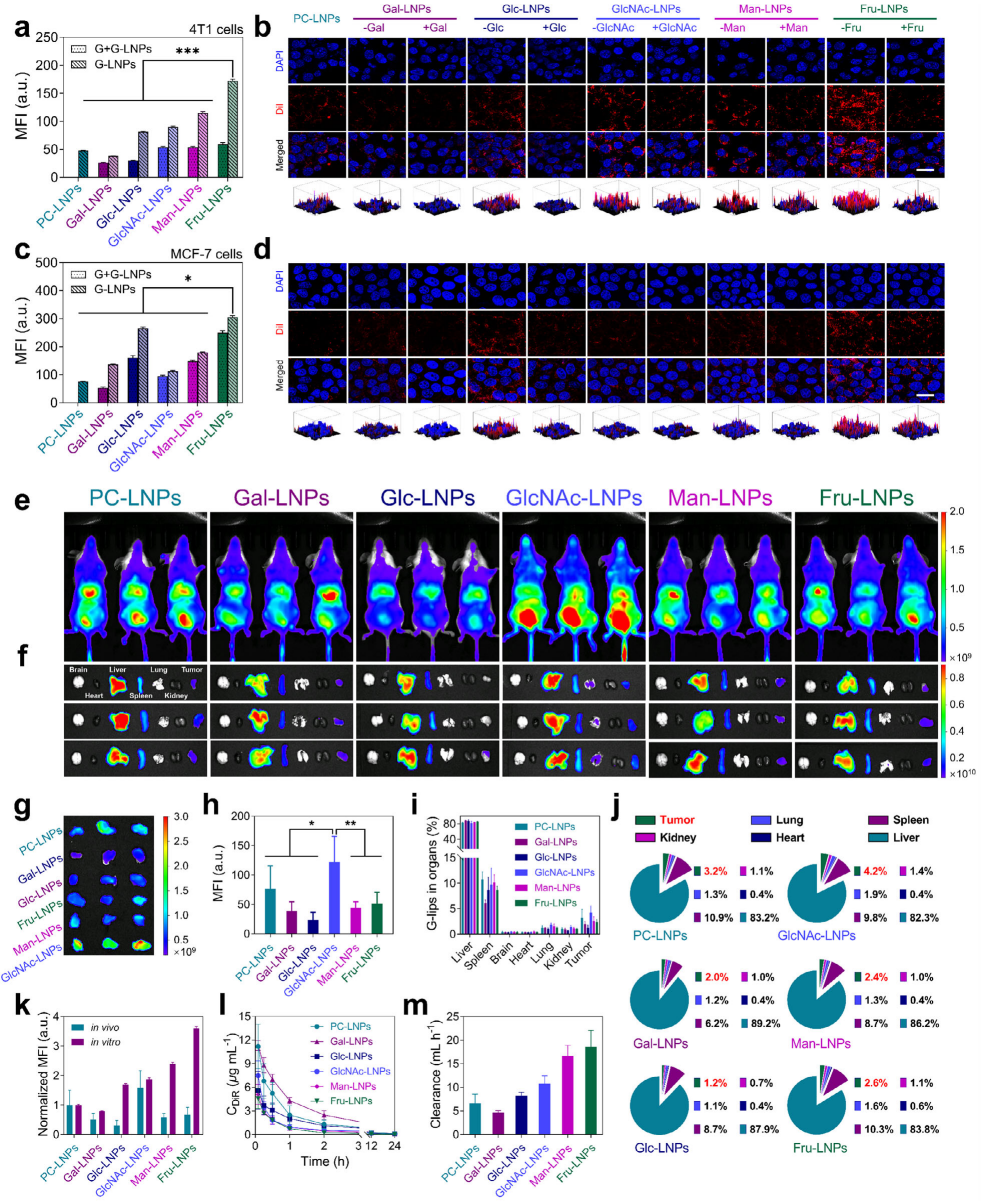
Tumor targeting and pharmacokinetic behaviors of G‐LNPs. (a) Flow cytometry analysis and (b) CLSM images of G‐LNPs uptake in 4T1 cells. Scale bars: 20 *µm*. (c) Flow cytometry analysis and (d) CLSM images of G‐LNPs uptake in MCF‐7 cells (*n* = 3). G‐LNPs were labeled with DiI for cellular uptake experiments. Scale bars: 20 *µm*. (e) In vivo fluorescence images of mice and (f) In vitro fluorescence images of major organs at 24 h post intravenous injection of G‐LNPs (*n* = 3). (g) In vitro fluorescence images of tumors (*n* = 3). (h) Quantitative analysis of the tumor fluorescence intensity of G‐LNPs from in vitro imaging (*n* = 3). (i) Quantitative analysis of the percentage of the fluorescence intensity of G‐LNPs in all major organs from in vitro imaging (*n* = 3). (j) Pie charts depicting the fluorescence intensity distribution percentages of G‐LNPs in major organs (*n* = 3). (k) Normalized comparison of in vivo and in vitro tumor targeting efficiency of various G‐LNPs (*n* = 3). (l) Blood concentration‐time curves of G‐LNPs (*n* = 6). (m) Total clearance of G‐LNPs (*n* = 6). G‐LNPs were labeled with DiR for in vivo experiments. Data are presented as mean ± SD. Statistical significance was tested with ANOVA. *p < 0.05, **p < 0.01, ***p < 0.001.

First, we investigated how glycosylation affects cellular uptake of LNPs by breast cancer cells. As shown in Figure 2a,b, the uptake of LNPs notably increased upon glycosylation with Fru, Man, GlcNAc, and Glc ligands, though not with Gal. Among these, Fru‐LNPs demonstrated the highest uptake efficiency. To exclude the potential contribution of surface charge to the observed differences in cellular uptake of G‐LNPs, PA‐LNPs were included as a charge‐matched control. Compared with PC‐LNP, PA‐LNPs exhibited a slight increase in uptake, whereas G‐LNPs showed relatively higher uptake profiles (except for Gal‐LNPs) (Figure S17). This result thus attributes the enhanced tumor cell uptake of G‐LNPs specifically to glycosylation, rather than to surface charge effects. To examine the contribution of ligands to endocytosis, we conducted competitive inhibition experiments where cells were preincubated with corresponding saccharides to saturate the receptors on cell membranes. The results showed a significant reduction in 4T1 uptake for all G‐LNPs after saccharide preincubation (Figure 2a,b), revealing a ligand‐mediated uptake mechanism. This result was further validated by the cell uptake results conducted at 4°C, which showed a significantly lower uptake compared to cell uptake performed at 37°C (Figure S18), thereby verifying the energy‐dependence of the receptor‐mediated endocytosis. Consistent uptake patterns were observed in MCF‐7 cells (Figures 2c,d and S19, S20), further validating the glycosylation‐dependent differences in cellular uptake.

We further investigated the biodistribution of various G‐LNPs using orthotopic 4T1 tumor‐bearing mice. As shown in Figures 2e,f and S21a, b, tumor accumulation differed markedly among the LNPs. GlcNAc‐LNPs exhibited the highest fluorescence signal at the tumor site, whereas Gal‐LNPs, Glc‐LNPs, Man‐LNPs, and Fru‐LNPs showed tumor accumulation comparable to PC‐LNPs or PA‐LNPs (Figures 2 g,h and S21c, d). Notably, Glc‐LNPs displayed low tumor accumulation in vivo despite its high cellular uptake observed in vitro. Organ‐level quantification revealed that the liver and spleen were the dominant clearance sites, accounting for > 80% and 6.2%–10.9% of G‐LNP accumulation, respectively, while < 5% reached the tumor (Figures 2i and S21e). Consistent with this, Glc‐LNPs achieved only ∼ 1.2% tumor accumulation in vivo, whereas GlcNAc‐LNPs, despite moderate in vitro uptake, showed the highest tumor accumulation (∼ 4.2% of injected dose; Figures 2j and S21f).

To better illustrate the differential targeting behaviors of various G‐LNPs in vitro and in vivo, we individually normalized the fluorescence intensity measured for in vitro 4T1 cell uptake and in vivo tumor accumulation (Figure 2k). The results showed distinct tumor targeting behaviors of G‐LNPs, particularly Fru‐LNPs, between in vitro and in vivo settings, underscoring the challenge of translating in vitro active targeting efficacy to complex in vivo environments. This finding was further supported by pharmacokinetic analysis, revealing overall rapid clearance of G‐LNPs compared with PC‐LNPs (Figures 2l and S22a), with blood concentrations declining rapidly within the first hour and leveling off after 4 h. Detailed analysis of systemic clearance showed varying degrees of accelerated clearance for Glc‐, Man‐, GlcNAc‐, and Fru‐modified LNPs compared to PC‐LNPs (Figures 2m and S22b), with Fru‐LNPs exhibiting the highest clearance rate and lowest AUC value (Figure S23). Notably, despite exhibiting comparable negative surface charges, PA‐LNPs were cleared more slowly than the G‐LNPs (Figures 2m, S18b), indicating that the accelerated clearance of G‐LNPs is driven primarily by the glycosyl ligands rather than by surface charge. This rapid clearance may contribute to the loss of targeting efficiency in vivo.

**FIGURE 3:**
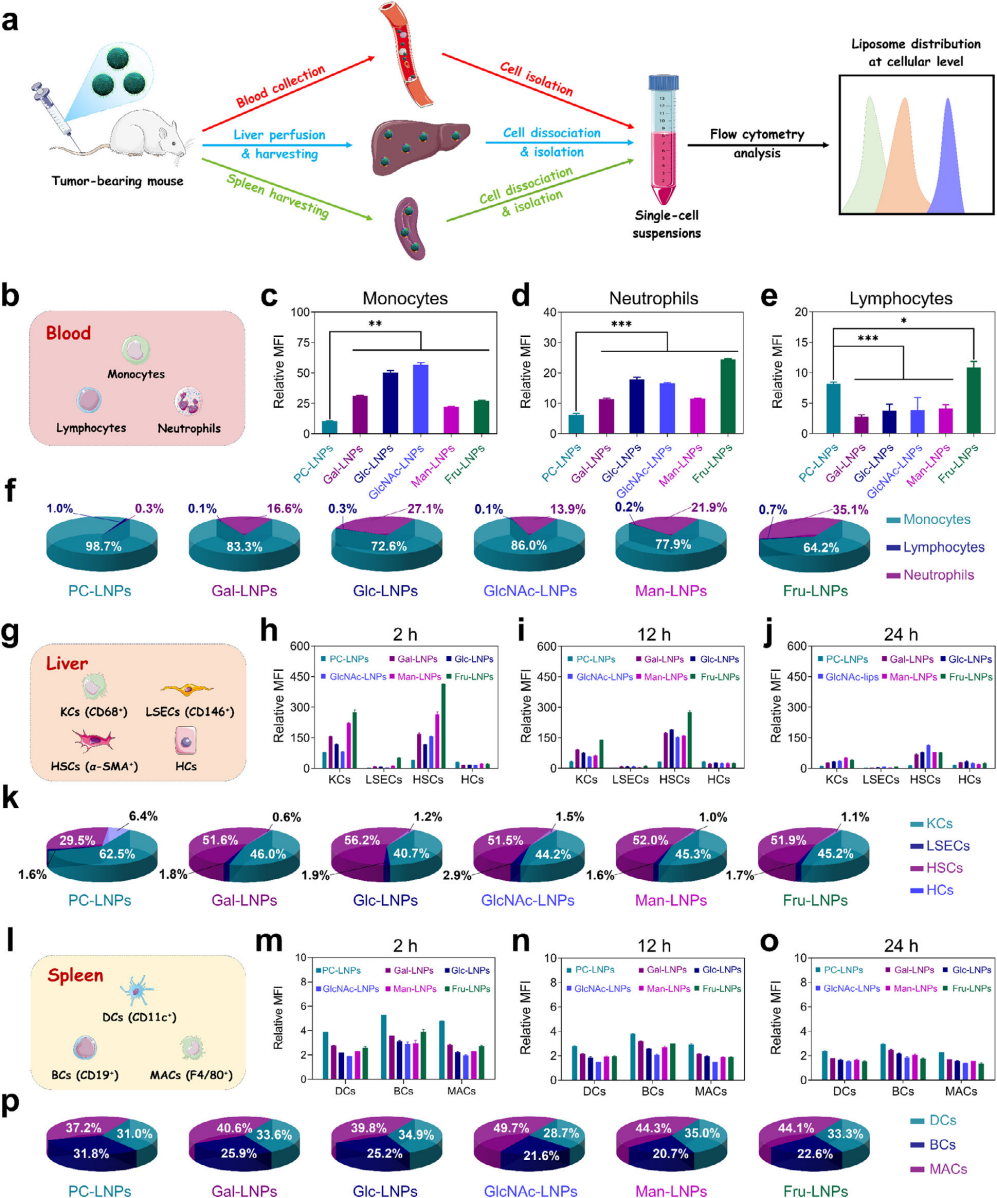
Cell tropism of G‐LNPs in the blood, liver, and spleen. (a) Schematic illustration of the procedure for isolating different cell types from blood, liver, and spleen. (b) Illustration of the 3 blood cell types primarily responsible for G‐LNPs clearance in the blood. After intravenous injection of G‐LNPs for 0.5 h, monocytes, lymphocytes, and neutrophils were isolated from mouse peripheral blood using hemocyte isolation kits. (c–e) Relative mean fluorescence intensity (MFI) of G‐LNPs uptake by blood monocytes (c), neutrophiles (d), and lymphocytes (e), detected via flow cytometry at 0.5 h post‐injection of DiD@G‐LNPs (*n* = 3). (f) Contribution of G‐LNPs clearance by the three blood scavenger cells at 0.5 h post‐injection. Contribution of cell_1_ (%) = (*Relative MFI_cell1_* × *number of cell_1_*)/ (*Relative MFI_cell1_* × *number of cell_1_* + *Relative MFI_cell2_* × *number of cell_2_* + *Relative MFI_cell3_* × *number of cell_3_*) × 100%. (g) Schematic diagram of the four hepatic cell types primarily responsible for G‐LNPs clearance in the liver. After intravenous injection of G‐LNPs for 2, 12, or 24 h, the liver was perfused with collagenase IV to dissociate cells. Hepatic cells were isolated, labeled with specific antibodies, and analyzed for G‐LNPs uptake using flow cytometry. Hepatocytes (HCs) were isolated by centrifugation, while non‐parenchymal cells (NPCs) were labeled with antibodies: AF488‐conjugated anti‐mouse CD68 antibody (for KCs), PE‐conjugated anti‐mouse CD146 antibody (for LSECs), and *α*‐SMA anti‐mouse antibody (for HSCs). Gating strategies are detailed in Figure S27. Representative flow cytometry plots for 2, 12, and 24 h results are provided in Figures S38–S30. (h–j) Relative MFI of G‐LNPs uptake in different hepatic cells at 2 , 12, and 24 h post‐injection. (k) Contribution of 4 hepatic cell types to G‐LNPs clearance at 12 h post‐injection, calculated using the same formula as for blood cells. (l) Schematic diagram of three antigen‐presenting cells (APCs) primarily responsible for G‐LNPs clearance in the spleen. Splenocyte suspensions were stained with allophycocyanin‐conjugated anti‐mouse CD11c antibody (for DCs), FITC‐conjugated anti‐mouse F4/80 antibody (for MACs), or PE‐Cy7‐conjugated anti‐mouse CD19 antibody (for B cells). Full gating strategy is shown in Figure S31. Representative flow cytometry plots showing G‐LNPs uptake in APCs at 2, 12, and 24 h post‐injection are presented in Figures S32–S34. (m–o) Relative MFI of G‐LNPs uptake in different APCs at 2, 12, and 24 h post‐injection (*n* = 3). (p) Contribution of three APCs to G‐LNPs clearance at 12 h post‐injection, calculated using the same formula as for blood cells. Data are presented as mean ± SD. Statistical significance was tested with ANOVA. *p < 0.05, **p < 0.01, ***p < 0.001.

Phagocytes in the blood, primarily monocytes, lymphocytes, and neutrophils, play a crucial role in nanoparticle clearance from blood circulation [37, 38, 39]. Hence, the distribution of G‐LNPs among these key scavenger cells was analyzed (Figure 3a,b). As shown in Figure 3c, the relative mean fluorescence intensity (MFI) of DiD in monocytes was significantly higher for all G‐LNPs compared to PC‐LNPs. Notably, the percentages of G‐LNPs‐positive monocytes were close to 100% across different G‐LNPs, suggesting high uptake ability of monocytes (Figure S24). A similar trend was observed for neutrophils (Figure 3d), where both the relative MFI and the percentage of G‐LNPs‐positive cells (%DiD^+^ cell) were markedly higher for all G‐LNPs relative to PC‐LNPs (Figure S25). In contrast, lymphocytes showed significantly lower relative MFI and %DiD^+^ cells for G‐LNPs, except Fru‐LNPs, when compared to PC‐LNPs (Figures 3e and S26). These results suggest that glycosylation enhances G‐LNPs uptake by blood phagocytes, particularly monocytes and neutrophils. To identify the predominant phagocyte responsible for G‐LNPs clearance, we calculated the contribution of each cell type by integrating relative MFI and %DiD^+^ cell data. Monocytes were found to account for the majority of G‐LNPs clearance (64.2%–98.7%), while neutrophils (0.3%–35.1%) and lymphocytes (0.1%–1.0%) contributed significantly less (Figure 3f). Glycosylation reduced monocyte and lymphocyte contributions to liposomal nanoparticle uptake but significantly increased neutrophil involvement. Beyond blood clearance, liver and spleen also contribute to the in vivo liposomal nanoparticle clearance, evidenced by their pronounced accumulation of G‐LNPs.

### 2.3 Hepatic Clearance Mechanisms of G‐LNPs

Liver, a highly perfused organ constituting 10%–15% of total blood volume, plays a crucial role in nanoparticles clearance from circulation [40], primarily mediated by hepatic resident macrophages (Kupffer cells, KCs) and liver sinusoidal endothelial cells (LSECs) [41]. Given the significant accumulation of G‐LNPs in the liver, we conducted a detailed analysis of their distribution across various hepatic cell types, including KCs, LSECs, hepatic stellate cells (HSCs), and hepatocytes (HCs) in tumor‐bearing mice, following the injection of DiD‐labeled glycosylated liposomal nanoparticles (DiD@G‐LNPs) at 2, 12, and 24 h. The aim was to identify the primary cell types responsible for hepatic clearance and to evaluate the impact of glycosylation on hepatic clearance of G‐LNPs (Figures 3a–g and S27). Overall, KCs and HSCs exhibited markedly higher uptake of LNPs compared to LSECs and HCs, with glycosylation significantly enhanced their uptake (Figure 3h). Notably, GlcNAc‐LNPs demonstrated a substantially lower uptake across all four cell types. In contrast, Fru‐LNPs displayed the highest uptake in KCs, LSECs and HSCs (Figure 3 h,i), aligning with its rapid clearance profile from blood circulation (Figure 2m). Over time, MFI and %DiD^+^ cell among these hepatic populations gradually decreased (Figures 3h–j and S28–S30). However, DiD^+^ HCs consistently remained near 100%, likely due to ongoing liposomal transport among diverse hepatic cells. The high percentage of DiD^+^ HCs, despite their relatively low uptake at the single‐cell level, can be attributed to the large population of HCs in the liver. Conversely, LSECs exhibited the lowest MFI and %DiD^+^ cells among the four hepatic cells, indicating a minimal role in hepatic clearance. We further quantified the contribution of each hepatic cell type to G‐LNPs by integrating the data of MFI and %DiD^+^ cells. Results revealed that HSCs accounted for the highest uptake, followed by KCs, while HCs and LSECs contribute only 0.6%–6.4% and 1.6%–2.9%, respectively (Figure 3k).

### 2.4 Splenic Clearance Mechanisms of G‐LNPs

Spleen, as a key immune organ, plays a central role in clearing blood‐borne antigens, including intravenously injected G‐LNPs [42]. The spleen is rich in antigen‐ presenting cells (APCs), such as dendritic cells(DCs), macrophages (MACs), and B cells (BCs). Therefore, it is essential for antigen presentation, immune activation, and antigen‐mediated immune clearance [43]. As exogenous substances, G‐LNPs can be recognized by APCs, triggering immune responses that accelerate their clearance from circulation. To investigate this process, we analyzed APCs uptake of G‐LNPs in tumor‐ bearing mice (Figures 3a,l and S31). Compared to PC‐LNPs, G‐LNPs demonstrated a significantly reduced uptake by APCs (Figure 3m–o), suggesting that glycosylation mitigates APC recognition and uptake contrary to the enhanced uptake observed in the liver. Notably, GlcNAc‐LNPs consistently showed the lowest uptake and %DiD^+^ rates across all APC populations from 2 to 24 h post‐injection (Figures S32–S34), consistent with hepatic clearance patterns, which may contribute to the reduced in vivo clearance of GlcNAc‐LNPs. Among the APC subtypes, uptake by DCs, BCs, and MACs were comparable, while MACs showed a significantly higher %DiD^+^ rate, underscoring their dominant role in immune clearance. Further analysis of the relative contributions of APC subtypes to nanoparticle clearance revealed that DCs, BCs, and MACs contributed comparable to PC‐LNPs clearance (31.0%‐37.2%, Figure 3p). In contrast, for G‐LNPs, MACs played a more prominent role, while the contribution of B cells decreased. These findings indicate that glycosylation attenuates splenic immune clearance of G‐LNPs, with macrophages serving as the principal mediators of this process.

### 2.5 Protein Corona Composition Regulates In Vitro Phagocyte and Immune Cell Uptake of G‐LNPs

**FIGURE 4:**
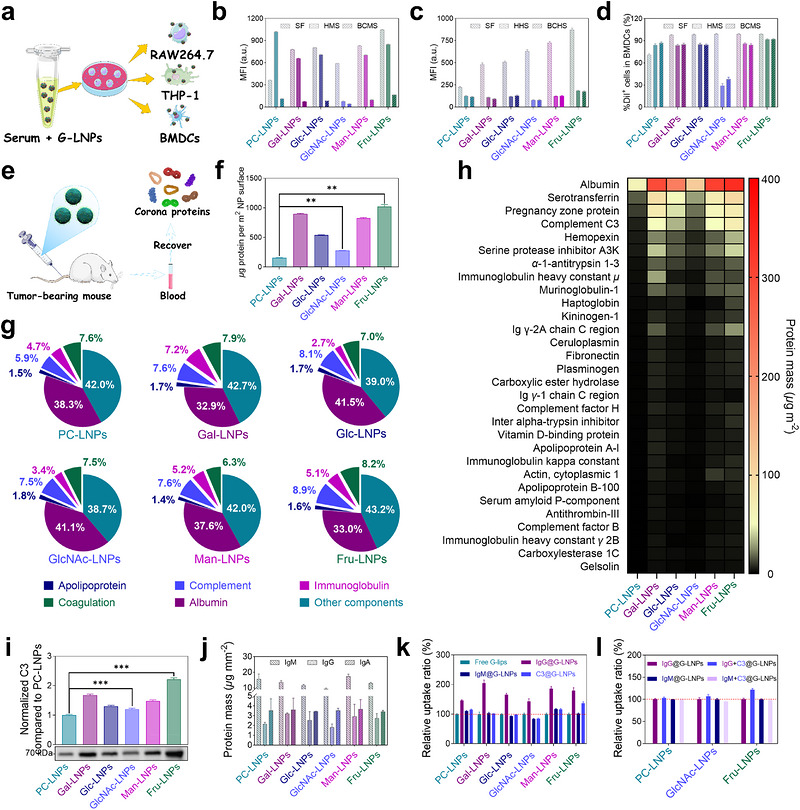
In vitro phagocyte and immune cell uptake and proteomic analysis of the protein corona. (a) Schematic diagram describing the uptake of G‐LNPs by three major clearance cells following incubation with various sera. (b) Uptake of mouse serum‐incubated G‐LNPs by mouse‐derived macrophage RAW264.7 cells (*n* = 3). (c) Uptake of human serum (healthy human serum, HHS or breast cancer human serum, BCHS)‐incubated G‐LNPs by human‐derived macrophage THP‐1 cells (*n* = 3). (d) Uptake of mouse serum‐incubated G‐LNPs by mouse bone marrow dendritic cells (BMDCs) (*n* = 3). (e) Schematic diagram illustrating the isolation process of the protein corona from tumor‐bearing mice following G‐LNPs injection. (f) Quantification of adsorbed proteins on G‐LNPs (*µg* protein per m^2^ NP surface) recovered from murine blood (*n* = 3). (g) All proteins identified by quantitative LC‐MS/MS were categorized into six different classes based on their biological function. (h) Heat map of the 30 most abundant proteins detected in the protein corona of G‐LNPs isolated from mice. (i) Western blot assay of complement C3 adsorbed on the surface of G‐LNPs. (j) Quantification of relative IgG/IgM/IgA contents in the protein corona of G‐LNPs isolated from mice. (k) Relative uptake ratio of G‐LNPs upon preincubation with C3/IgM/IgG by RAW264.7 cells (*n* = 3). Relative uptake ratio = *MFI_IgG/IgM/C3@G‐LNPs_* /*MFI_G‐LNPs_*, with *MFI_G‐LNPs_* defined as 100%. (l) Relative uptake ratio of G‐LNPs upon preincubation with IgG+C3 or IgM+C3 by RAW264.7 cells (*n* = 3). Relative uptake ratio = *MFI_IgG+C3@G‐LNPs or IgM+C3@G‐LNPs_*/*MFI_IgG@G‐LNPs or IgM@G‐LNPs_*, with *MFI_IgG@G‐LNPs_* and *MFI_IgM@G‐LNPs_* defined as 100%, respectively. Data are presented as mean ± SD. Statistical significance was tested with ANOVA. *p < 0.05, **p < 0.01, ***p < 0.001.

Phagocytes and antigen‐presenting cells such as DCs play a vital role in clearing nanoparticles from blood circulation. First, we studied the uptake of G‐LNPs by classical model phagocytes upon incubation with sera from mice and human under healthy/breast cancer status (Figure 4a). Mouse‐derived RAW264.7 and human‐derived THP‐1 macrophages, common models for studying nanoparticle clearance, showed lower uptake of serum‐incubated LNPs compared to serum‐free (SF, replace serum with PBS) conditions (Figure 4b,c). Incubation with mouse serum significantly influenced nanoparticle uptake by RAW264.7 cells, with healthy mouse serum (HMS) enhancing uptake and breast cancer mouse serum (BCMS) inhibiting uptake (Figure 4b), indicating the crucial effect of disease status on macrophages clearance. Notably, GlcNAc‐LNPs consistently exhibited the lowest uptake by RAW264.7 cells, while Fru‐LNPs showed the highest among G‐LNPs. To assess the influence of LNP surface charge on macrophage uptake, PA‐LNPs with a negative charge comparable to G‐LNPs were included as a control. GlcNAc‐LNPs consistently exhibited the lowest uptake by RAW264.7 cells, whereas Fru‐LNPs showed the highest uptake (Figure S35), indicating that surface‐displayed glycosyl ligands, rather than surface charge, predominantly govern the differential macrophage uptake of G‐LNPs. Consistent trend was observed in THP‐1 cells using human serum as the protein source, where glycosylation similarly modulated LNP uptake, with GlcNAc‐LNPs showing the lowest uptake (Figures 4c and S36). However, the differential RAW264.7 cells uptake between healthy and diseased mice serum incubation was not observed when using human sera and THP‐1 cells. To further investigate whether this distinct cell uptake behaviors was due to species‐differences of protein corona or macrophages, we conducted experiments utilizing protein corona from human serum and uptake by mouse‐derived macrophages RAW264.7 cells (Figure S37). The results were similar to those with human sera and THP‐1 cells (Figure 4c), thus suggesting that this disease‐caused difference in mice was stemming from the species‐dependent protein corona rather than disparities in cell species. Overall, these findings unveiled that both species and disease status of protein sources can influence macrophages clearance of LNPs.

To explore the immune clearance of G‐LNPs by APCs, we isolated mouse bone marrow dendritic cells (BMDCs) and differentiated them into an immune‐clearance cell model (Figure S38). To assess the impact of protein corona on BMDCs uptake of G‐LNPs, we used three conditions: incubation with PBS (serum‐free conditions, SF), HMS, or BCMS. As shown in Figure 4d, under SF conditions (i.e., in the absence of a protein corona), BMDCs uptake exhibited minimal variation across all G‐LNPs variants. However, preincubation with mouse serum led to a notable reduction in uptake, indicating that the mouse protein corona inhibited interactions between G‐LNPs and BMDCs. When comparing protein corona‐coated G‐LNPs derived from HMS and BCMS, no significant difference in BMDCs uptake was observed. This result contrasts with the trend seen in macrophages, where the disease state of serum source affected G‐LNPs uptake (Figure 4b). Notably, among the G‐LNPs variants, GlcNAc‐LNPs consistently showed the lowest positivity rate of cell uptake (HMS: 29.1%, BCMS: 37.7%), whereas Fru‐LNPs showed the highest (HMS: 91.6%, BCMS: 92.0%), regardless of the serum type. This trend aligns with the results of macrophage uptake (Figure 4b). Collectively, these findings demonstrated that variations in glycosyl ligands significantly influenced APC‐mediated immune clearance mechanisms.

To explore how the protein corona influences liposomal nanoparticle clearance in vivo, we injected the LNPs into tumor bearing‐mice and analyzed the recovered in vivo protein corona using BCA protein assay and LC‐MS/MS (Figure 4e). Quantitative analysis revealed that G‐LNPs adsorbed significantly more proteins than PC‐LNPs (155.3 *µg* m^−2^ nanoparticle surface), with GlcNAc‐LNPs adsorbing the least (279.2 *µg* m^−2^) and Fru‐LNPs the most (1020.3 *µg* m^−2^) (Figure 4f). Compared with PC‐LNPs, protein adsorption on PA‐LNPs only slightly increased (0.4‐fold increase, 216.7 *µg* m^−2^) (Figure S39), whereas Fru‐LNPs and GlcNAc‐LNPs showed 5.6‐ and 0.8‐fold increases, respectively. These results indicate that glycosylation, rather than surface charge, predominantly governs protein corona formation, correlating with macrophage uptake trends and suggesting that enhanced protein adsorption may drive accelerated in vivo clearance of G‐LNPs. LC‐MS/MS analysis revealed a preferential adsorption for smaller plasma proteins (< 70 kDa, Figure S40) and negatively charged proteins (isoelectric point < 7, Figure S41) across all LNPs. Although the types of adsorbed proteins were similar among LNPs (Figure S42), their abundance differed significantly. The proteins were categorized into six classes based on their biological functions (Figures 4g and S43). In addition to albumin, which is the most abundant blood protein and constitutes 27.3%‐41.5% of the protein corona, specific low‐abundance proteins such as apolipoproteins (1.4%–2.3%), complement proteins (3.8%–8.9%), immunoglobulins (2.7%–7.2%), and coagulation proteins (5.2%–8.2%), had also been reported to play pivotal roles in regulating biological fate of nanomedicines. For example, adsorption of complement proteins and immunoglobulins as opsonins could promote the immune recognition and clearance of nanocarriers [31, 44, 45, 46]. Enrichment of apolipoproteins, for example, Apo A1 and Apo J (clusterin), in the protein corona endows nanoparticles a stealth behavior, prolonging their blood circulation [32, 47]. Fibrinogen is involved in blood coagulation and potentially mediates the aggregation of nanoparticles [48]. Among the top 30 most abundant proteins, albumin was a dominant component on all LNP surfaces, with markedly higher enrichment on Fru‐LNPs and the lowest level on PC‐LNPs (Figures 4h and S44). In parallel, Fru‐LNPs displayed the highest recruitment of complement proteins, and PC‐LNPs had the least. Quantitative analysis showed that complement C3 adsorption reached 69.3 *µg* m^−2^ on Fru‐LNPs, whereas GlcNAc‐LNPs and PC‐LNPs displayed substantially lower levels of 15.1 and 6.0 *µg* m^−2^, respectively (Figure S45). A similar trend was observed for immunoglobulins, with minimal adsorption detected on GlcNAc‐LNPs and PC‐LNPs (Figure S46). To decouple the effect of surface charge from glycosylation, PA‐LNPs was included as a charge‐matched control. In contrast to glycosylated LNPs, PA‐LNPs showed only slight increases in albumin, complement C3 (6.1 *µg*·m^−2^), and immunoglobulin adsorption relative to PC‐LNPs (Figures 4h and S44–S46). These findings indicate that glycosylation, rather than surface charge, plays a dominant role in shaping the protein corona. Notably, the observed protein adsorption patterns were consistent with the in vivo clearance behavior of the LNPs, implicating complement C3 and immunoglobulins as potential contributors to their differential in vivo fate. In addition, due to the strong nonspecific adsorption and low elution efficiency, the yield of purified glycolipids was insufficient to support extensive downstream biological studies, particularly in vivo experiments requiring large material quantities. Thus, the synthesized glycolipids were directly used for G‐LNP preparation without additional purification. Given that using PA‐LNPs as control consistently proved the superior biological effects (Figures 2h–m and S17, S21, S22) arise from the glycosylated headgroup rather than from the PA impurity, the use of glycolipids without further purification would not change the main conclusions of this study.

Complement C3 (C3) is a crucial factor facilitating opsonization and initiating downstream immune responses [49]. Its cleaved fragment, C3b, plays a pivotal role in immune recognition and phagocytic clearance of nanoparticles [50, 51]. Western Blot analysis (Figure 4i) revealed consistent trends between C3 adsorption, proteomics data (Figures 4h and S45), and phagocytosis results (Figure 4b–d). Specifically, GlcNAc‐LNPs demonstrated the least C3 adsorption and the lowest uptake by RAW264.7 cells and BMDCs, decreased by 15.0% ± 1.6%). This result was in accordance with the quantitative results of C3 adsorption on these two G‐LNPs determined by Western Blot (Figure 4i). These findings verified the critical role of complement C3 in modulating G‐LNPs clearance. Because all complement activation pathways converge at C3, subsequently producing C3a and C5a [52, 53], we used an enzyme‐linked immunosorbent assay (ELISA) to measure serum C3a and C5a levels to evaluate the overall complement activation. As shown in Figures S47–S48, C3a and C5a levels were lower in all G‐LNPs groups compared to the PBS group, particularly with GlcNAc‐LNPs exhibiting the lowest C3a and C5a levels, implying potential complement inhibitory activity of *N*‐acetylamino‐D‐glucose. The reduced C3a and C5a levels in G‐LNPs can be attributed to the C3 stabilization on their surface, which prevents further cleavage and downstream activation of the complement system.

Immunoglobulins, such as immunoglobulin G (IgG) and immunoglobulin M (IgM), are key mediators of antigen recognition and macrophage‐mediated clearance [31, 54]. Proteomics analysis revealed significant immunoglobulin adsorption on G‐LNPs (Figures 4h and S46), although the specific immunoglobulin types could not be distinguished. To clarify their roles in regulating liposomal nanoparticles clearance, we quantified the three primary sera immunoglobulins in the protein corona using ELISA measurements. The results showed that IgM was the most abundant (> 60%), while IgA and IgG accounted for only 15.3%–24.2% and 10.1%–15.6% of adsorption, respectively (Figure S49). We further analyzed the absolute quantity of immunoglobulins adsorbed onto G‐LNPs. As shown in Figures 4j, G‐LNPs exhibited lower IgM adsorption compared to PC‐LNPs, with GlcNAc‐LNPs displaying the lowest IgM levels. In contrast, IgA adsorption was consistent across all G‐LNPs variants, excluding its responsibility for the differential in vivo clearance of the G‐LNPs variants. For IgG, GlcNAc‐LNPs also showed the lowest adsorption, whereas other G‐LNPs adsorbed more IgG than PC‐LNPs. These results indicated that IgG and IgM may be involved in the clearance process of G‐LNPs. To validate this hypothesis, we preincubated G‐LNPs with IgG or IgM and investigated their uptake by RAW264.7 cells. As shown in Figure 4k, IgG preincubation significantly enhanced G‐LNPs uptake, while IgM preincubation only slightly increased it, indicating that IgG plays a more prominent role in macrophage uptake of G‐LNPs compared to IgM. Recent studies have shown that IgG and IgM can bind to nanomedicines and expose complement‐binding sites, thereby accelerating recognition and clearance by macrophages through increased complement C3 deposition [31, 55]. To further clarify which immunoglobulin (IgG or IgM) interacts with C3 in mediating G‐LNPs clearance, we examined the uptake of GlcNAc‐LNPs and Fru‐LNPs preincubated with IgG+C3 or IgM+C3 by RAW264.7 cells. As shown in Figure 4l, preincubation with C3+IgG markedly increased the uptake of Fru‐LNPs by 22.0% ± 2.4% compared to G‐LNPs preincubated with IgG alone (IgG@G‐LNPs). However, preincubation with C3+IgM did not result in a detective increase. Similarly, C3+IgG preincubation increased GlcNAc‐LNPs uptake by 7.1% ± 3.4%, whereas preincubation with C3+IgM slightly decreased their uptake (4.1% ± 1.7%). Notably, even though IgM was more abundantly adsorbed than IgG on G‐LNPs (Figure 4j), G‐LNPs preincubated with C3+IgG significantly enhanced macrophage uptake compared to C3+IgM preincubation, implying that the interaction between C3 and IgG was predominant in modulating the phagocytic clearance of G‐LNPs.

### 2.6 Quantitative Validation of the Effects of Glycosyl Ligand Density on IgG&C3 Adsorption and Macrophage Uptake

**FIGURE 5:**
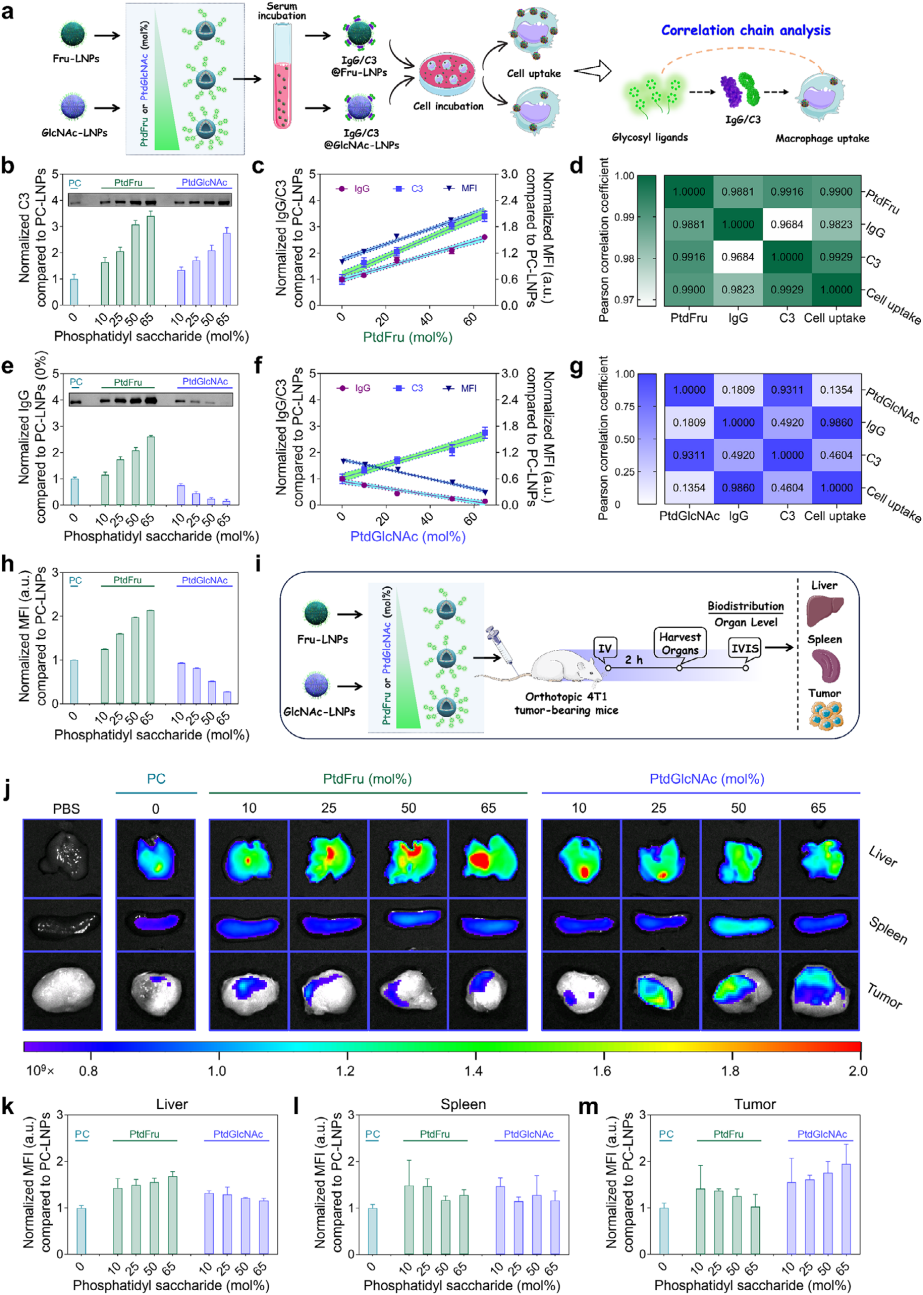
Correlation analysis among glycosyl ligand density, IgG&C3 adsorption, and cellular uptake by RAW264.7 cells. (a) Schematic illustration showing how variations in the molar ratio of PtdFru or PtdGlcNAc affects G‐LNPs uptake by RAW264.7 cells. (b) Western blot analysis of C3 adsorbed on G‐LNPs (*n* = 3). (c) Linear regression analysis of IgG and C3 adsorption levels versus Fru‐LNPs uptake by RAW264.7 cells (*n* = 3). (d) Pearson correlation coefficients among PtdFru molar ratio, IgG/C3 adsorption, and Fru‐LNPs uptake by RAW264.7 cells. (e) Western blot analysis of IgG adsorbed on G‐LNPs (*n* = 3). (f) Linear regression analysis of IgG and C3 adsorption levels versus GlcNAc‐LNPs uptake by RAW264.7 cells (*n* = 3). (g) Pearson correlation coefficients for determining correlation among PtdGlcNAc molar ratio, IgG/C3 adsorption, and GlcNAc‐LNPs uptake by RAW264.7 cells. (h) Uptake of Fru‐LNPs or GlcNAc‐LNPs with varying molar ratios of PtdFru or PtdGlcNAc by RAW264.7 cells (*n* = 3). (i) Schematic illustration showing how variations in the molar ratio of PtdFru or PtdGlcNAc affect the biodistribution of G‐LNPs in major clearance organs (liver and spleen) and tumor. (j) In vitro fluorescence images of the major clearance organs (liver and spleen) and tumor at 2 h post intravenous injection of Fru‐LNPs or GlcNAc‐LNPs with varying molar ratios of PtdFru or PtdGlcNAc (*n* = 3). (k–m) Quantitative analysis of the liver (k), spleen (l), and tumor (m) fluorescence intensity of Fru‐LNPs or GlcNAc‐LNPs with varying molar ratios of PtdFru or PtdGlcNAc from in vitro imaging (*n* = 3). Data are presented as mean ± SD. Statistical significance was tested with ANOVA. *p < 0.05, **p < 0.01, ***p < 0.001.

To elucidate the impact of glycosyl ligands on protein corona composition and macrophage uptake, we selected GlcNAc‐LNPs, which exhibit the highest tumor accumulation, and Fru‐LNPs, which display the fastest in vivo clearance, for comparative analysis (Figure 5a). By adjusting the molar ratio of phosphatidyl saccharides from 0% to 65% in G‐LNPs, we systematically evaluated the correlations among glycosyl ligand density, key corona component abundance (IgG and C3), and macrophage uptake. Both liposomal nanoparticle types exhibited a slight increase in hydrodynamic diameter and a notable increase in the absolute value of zeta potential with increasing glycosylation density (Figures S50, S51). However, the magnitude of these changes was similar between Fru‐LNPs and GlcNAc‐LNPs, suggesting that size and surface charge alone are unlikely to account for the distinct biological behaviors observed.

As shown in Figure 5b, increasing the PtdGlcNAc content from 10% to 65% led to a progressive increase in C3 adsorption, with the increase of 33.9% to 175.5% relative to PC‐LNPs (0% PtdGlcNAc). Across all tested compositions, Fru‐LNPs consistently adsorbed 1.2‐ to 1.5‐fold more C3 than GlcNAc‐LNPs, indicating that both ligand density and glycosyl type influence C3 recruitment, with PtdFru displaying a stronger affinity for C3. For both G‐LNPs types, C3 adsorption correlated strongly with glycosyl ligand density (*r > 0.9*, Figures 5c,d). In contrast, IgG adsorption exhibited opposing trends (Figure 5e): Fru‐LNPs showed increasing IgG adsorption with rising glycosylation, while GlcNAc‐LNPs displayed a marked decrease. At the highest glycosylation level (65%), Fru‐LNPs adsorbed 17.8 times more IgG than GlcNAc‐LNPs, highlighting the importance of glycosyl ligand identity in modulating IgG binding, beyond simple density effects.

We further assessed macrophage uptake of protein corona‐coated G‐LNPs prepared in fresh serum from breast tumor‐bearing mice. As shown in Figure 5h, Fru‐LNPs exhibited increased uptake with higher glycosyl ligand density, whereas GlcNAc‐LNPs showed a decreasing trend. These uptake patterns closely aligned with the respective IgG adsorption profiles (Figure 5c,f), implicating IgG as a major determinant of phagocytic clearance. Correlation analysis revealed that, for Fru‐LNPs (Figure 5c,d), both IgG and C3 adsorption levels increased with glycosylation density and strongly correlated with macrophage uptake (*r > 0.9*), suggesting coordinated regulation of clearance by both proteins. In contrast, for GlcNAc‐LNPs (Figure 5f,g), macrophage uptake was positively correlated with IgG adsorption (*r > 0.9*) but showed an inverse and weak correlation with C3 levels (*0.3 ≤ r < 0.5*), indicating that IgG plays a more prominent role in their clearance. Collectively, these results demonstrate that the phagocytic clearance of G‐LNPs is jointly regulated by the glycosyl ligand density and type, which modulate the adsorption of key opsonins. Among these, IgG exerts a dominant influence, and a dose‐dependent relationship exists between glycosyl ligand density, protein corona composition, and macrophage uptake.

Next, we systematically evaluated the in vivo distribution of G‐LNPs with varying molar percentages of glycosyl ligands in major clearance organs (liver and spleen) and in tumor tissues. As shown in Figure 5j,k, increasing glycosyl ligand content led to ligand‐dependent distribution profiles: PtdFru progressively enhanced hepatic fluorescence signals, whereas PtdGlcNAc gradually reduced liver accumulation. In the spleen, both PtdFru and PtdGlcNAc‐modified G‐LNPs showed reduced accumulation to varying degrees (Figure 5j,l). Notably, tumor accumulation of GlcNAc‐LNPs increased steadily with higher PtdGlcNAc content and reached a maximum at 65 mol%, while Fru‐LNPs showed an opposite trend (Figure 5m). Based on these in vivo distribution profiles (minimized liver and spleen clearance combined with maximized tumor accumulation), the formulation containing 65 mol% glycolipid was selected as the optimal candidate for subsequent therapeutic evaluation.

### 2.7 Assessment of Therapeutic Efficacy of Doxorubicin‐loaded Glycosylated Liposomal Nanoparticles

**FIGURE 6:**
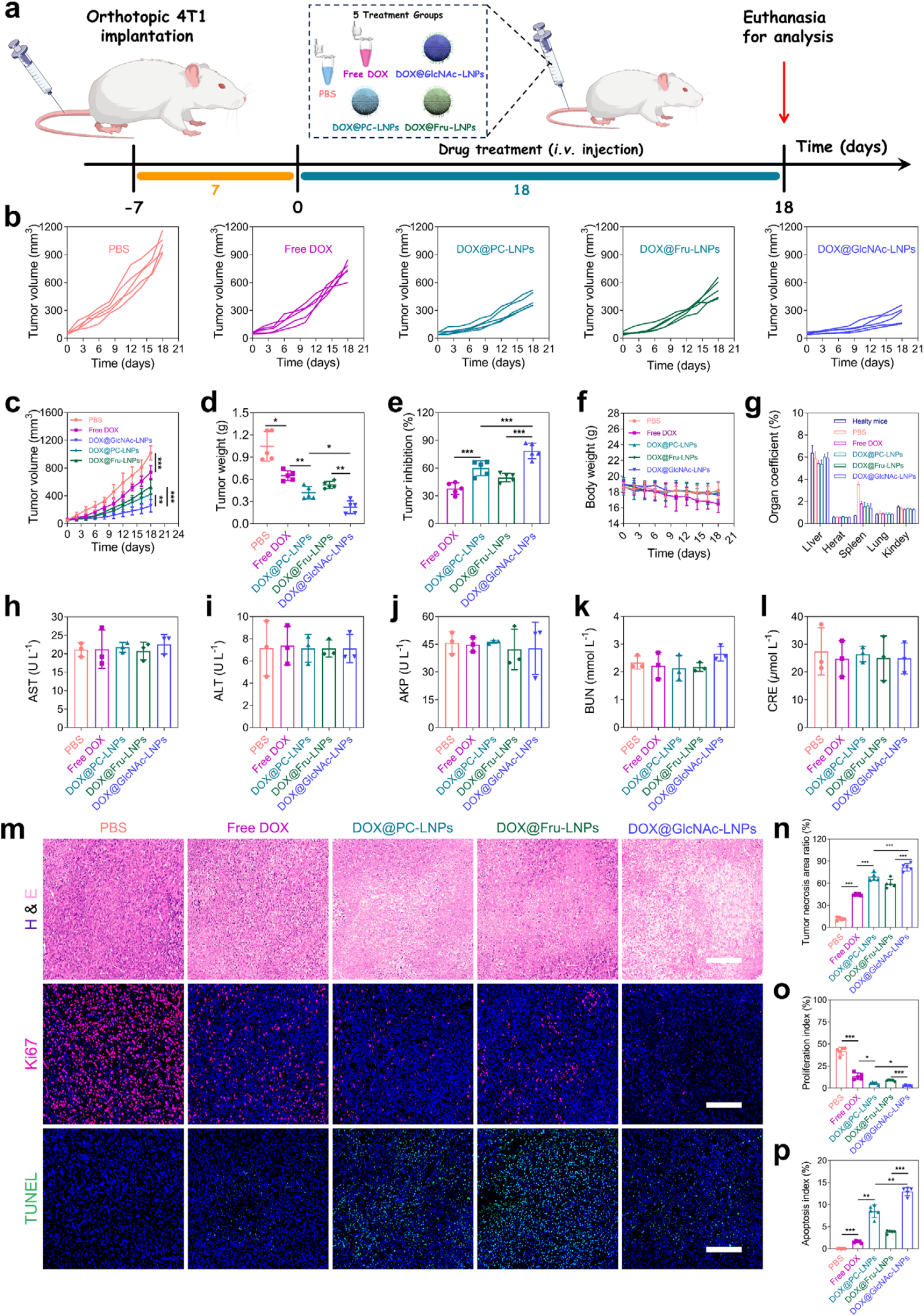
In vivo antitumor efficacy of DOX@G‐LNPs. (a) Schematic of the animal administration protocol in an orthotopic 4T1 tumor‐bearing mouse model. (b) Tumor growth curves for individual mice in different treatment groups over 18 days (*n* = 5). (c) Tumor volume changes over 18 days (*n* = 5). (d) Tumor weight on day 18 post‐treatment (*n* = 5). (e) Tumor growth inhibition rates compared to the PBS group (*n* = 5). (f) Body weight changes in mice over time following different treatments (*n* = 5). (g) Organ coefficient of each treatment group on day 18 (*n* = 5). (h–l), Blood biochemical indicators: AST (h), ALT (i), AKP (j), BUN (k), and CRE (l) (*n* = 3). (m) Representative images of H&E, Ki67, and TUNEL‐stained tumor sections in different treatment groups. Scale bar: 100 *µ*m. (n–p) Necrosis area ratio (%) of the H&E‐staining tumor sections (n), proliferation index (%) of the Ki67‐staining tumor sections (o), and apoptosis index (%) of the TUNEL‐staining tumor sections (p) quantified using Image J software (*n* = 5). Data are presented as mean ± SD. Statistical significance was tested with ANOVA. *p < 0.05, **p < 0.01, ***p < 0.001.

Based on the differential targeting profiles observed, where GlcNAc‐LNPs exhibited the highest tumor accumulation in vivo and Fru‐LNPs displayed the largest tumor cell uptake in vitro, we further evaluated the in vitro and in vivo antitumor efficacy of doxorubicin‐loaded glycosylated liposomal nanoparticles (DOX@G‐LNPs) (Table S4), specifically comparing doxorubicin‐loaded PC‐LNPs (DOX@PC‐LNPs), doxorubicin‐loaded GlcNAc‐LNPs (DOX@GlcNAc‐LNPs), and doxorubicin‐loaded Fru‐LNP (DOX@Fru‐LNPs). As shown in Figure S52, DOX@Fru‐LNPs demonstrated superior in vitro antitumor performance compared to DOX@GlcNAc‐LNPs and DOX@PC‐LNPs, attributed to enhanced cellular uptake (Figures S53, S54) and a higher apoptosis rate (Figure S55). In contrast, in vivo assessments using orthotopic 4T1 tumor‐bearing mice (Figure 6a) revealed that DOX@GlcNAc‐LNPs achieved the most potent tumor growth inhibition, with an inhibition rate of 78.6%, evidenced by changes of tumor volume (Figure 6b,c) and tumor weight (Figures 6d,e and S56). H&E, Ki67, and TUNEL staining further validated the superior in vivo antitumor efficacy of DOX@GlcNAc‐LNPs (Figure 6m–p). These therapeutic outcomes align with the observed targeting profiles: (i) GlcNAc‐LNPs, despite lower in vitro cell uptake, achieved greater tumor accumulation in vivo (Figure 2a–h); (ii) GlcNAc‐LNPs displayed the lowest uptake by scavenger cells, contrasting with the higher uptake seen with Fru‐LNPs (Figures 4b–d). The enhanced tumor accumulation of GlcNAc‐LNPs, likely due to reduced opsonin deposition and in vivo clearance, underpins its superior antitumor efficacy in vivo. Safety assessments showed no significant weight loss, pathological damage, or abnormal blood indicators in liposomal nanoparticle‐treated groups, indicating a favorable safety profile for DOX@G‐LNPs (Figures 6f–l and S57, S58). In summary, by modulating the surface glycosyl ligands of G‐LNPs, we are able to effectively regulate opsonin deposition (e.g., complement C3, IgG, or IgM) in the protein corona, thereby enhancing in vivo tumor accumulation and significantly improving antitumor efficacy.

## 3 Conclusion

We prepared five glycosylated liposomal nanoparticles to investigate the role of glycosylation and protein corona in modulating the in vivo behavior of liposomal nanoparticles. Among the saccharides examined, GlcNAc demonstrated a superior tumor accumulation and reduced systemic clearance compared to glucose, galactose, fructose, and mannose. Cellular analysis of clearance organs highlighted blood monocytes, hepatic stellate cells, and splenic macrophages as primary mediators of liposomal nanoparticle clearance. Proteomic studies revealed that IgG and complement C3 adsorption enhance liposomal nanoparticle clearance, with IgG promoting subsequent C3 binding. Importantly, GlcNAc modification significantly reduced the adsorption of both IgG and C3, resulting in prolonged circulation and enhanced tumor accumulation and therapeutic efficacy. This study establishes a clear correlation between glycosyl ligands, protein corona composition, and biological fate of LNPs. Our findings underscore the critical role of protein corona in regulating liposomal nanoparticle clearance, which is essential for understanding the in vivo fate and therapeutic efficacy of glycosylated nanomedicines and facilitating their rational design for clinical application.

## Conflicts of Interest

The authors declare no conflicts of interest.

## Supporting information




**Supporting File 1**: anie72114‐sup‐0001‐SuppMat.Docx.

## Data Availability

The data that support the findings of this study are available from the corresponding author upon reasonable request.
