# Supplementary material for: Incorporation of Novel Synthetic Glycolipids in Liposomal Nanoparticles Affects Opsonization and In Vivo Clearance
Source: Angew Chem Int Ed Engl. 2026 Apr 22;65(28):e20837. doi: 10.1002/anie.202520837 (PMC13340472; doi:10.1002/anie.202520837)
Supplement: Supplementary file 1 — Supporting File 1: anie72114‐sup‐0001‐SuppMat.Docx. [file ANIE-65-e20837-s001.docx]

Supporting Information

Incorporation of Novel Synthetic Glycolipids in Liposomal Nanoparticles Affects Opsonization and *In Vivo* Clearance

Yingjie Yu,^+^ Xuehan Li,^+^ Yu Gao, Shijia Tao, Xiaofei Li, Lemei Zhao, Wenshuai Han, Hao Fan, Ying Qiu, Man Wang, Luying Zhou, Xiaoyan Fang, Wenhua Yang, Haiyang Zhang, Volker Mailänder, Daniel Crespy, Katharina Landfester^*^, Shuai Jiang^*^, Xiangzhao Mao^*^

**Table of Contents**

[Supporting Information 1](#_Toc221177473)

[Table of Contents 2](#_Toc221177474)

[1. Experimental Section 3](#_Toc221177475)

[1.1 Materials 3](#_Toc221177476)

[1.2 Cell culture and animal care 4](#_Toc221177477)

[1.3 Synthesis and characterization of phosphatidyl saccharides 4](#_Toc221177478)

[1.4 Preparation and characterization of glycosylated liposomal nanoparticles 5](#_Toc221177479)

[1.5 Preliminary safety evaluation 6](#_Toc221177480)

[1.6 Uptake of G-LNPs by 4T1 and MCF-7 tumor cells 7](#_Toc221177481)

[1.7 Construction of orthotopic 4T1 tumor-bearing mice model 7](#_Toc221177482)

[1.8 Uptake of G-LNPs by RAW264.7 and THP-1 cells 7](#_Toc221177483)

[1.9 Uptake of G-LNPs by BMDCs 8](#_Toc221177484)

[1.10 Cytotoxicity of DOX@G-LNPs 8](#_Toc221177485)

[1.11 Apoptosis of 4T1 and MCF-7 tumor cells induced by DOX@G-LNPs 8](#_Toc221177486)

[1.12 Analysis of main cell populations for G-LNPs clearance in blood using flow cytometry 8](#_Toc221177487)

[1.13 Analysis of main cell populations for G-LNPs clearance in liver and spleen using flow cytometry 9](#_Toc221177488)

[1.14 Isolation and characterization of protein corona 9](#_Toc221177489)

[1.15 LC-MS/MS analysis 9](#_Toc221177490)

[1.16 Quantitative analysis of C3/IgG deposition on G-LNPs surface using western blot 10](#_Toc221177491)

[1.17 Quantitative analysis of immunoglobulins in protein corona using ELISA 10](#_Toc221177492)

[1.18 *In vitro* evaluation of effect of C3/IgG/IgM deposition on macrophage uptake of G-LNPs 10](#_Toc221177493)

[1.19 *In vitro* C3a/C5a determination using ELISA 10](#_Toc221177494)

[1.20 *In vivo* biodistribution imaging of G-LNPs 11](#_Toc221177495)

[1.21 Pharmacokinetics study 11](#_Toc221177496)

[1.22 *In vivo* anti-tumor efficacy 11](#_Toc221177497)

[1.23 Statistical analysis 12](#_Toc221177498)

[2. Results 13](#_Toc221177499)

[References 48](#_Toc221177500)

**1. Experimental Section**

**1.1 Materials**

L-*α*-phosphatidylcholine (purity 95.0%, from soybean) was purchased from Avanti Polar-Lipids, Inc. Phospholipase D (*Streptomyces* sp.) was heterologously expressed by *Bacillus subtilis* (*B. subtilis*), which has been constructed in our laboratory. D-Glucose (purity 99.8%), D-galactose (purity 99.0%), D-fructose (purity 99.0%), D-mannose (purity 99.0%), *N*-acetyl-D-glucosamine (purity 98.0%), recombinant mouse GM-CSF (Cat# P00184), recombinant mouse IL-4 (Cat# P00196), mouse IgG powder (Cat# SP031), and human IgG powder (Cat# SP001), Mouse Monocyte Extraction Kit (catalog number P5230), Mouse Neutrophil extraction kit (catalog number P9201), and Mouse Lymphocyte extraction kit (catalog number P8620) were purchased from Beijing Solarbio Science & Technology Co., Ltd. Cholesterol (powder, ≥ 99%), collagenase from *Clostridium histolyticum* (Type IV), and human IgM protein (Cat# I8260) were purchased from Merck. The dye 1,1'-dioctadecyl-3,3,3',3'-tetramethylindocarbocyanine perchlorate (purity 98.0%, DiI), 2-[5-(3,3-dimethyl-1-octadecylindol-1-ium-2-yl)penta-2,4-dienylidene]-3,3-dimethyl-1-octadecylindole (purity 98.0%, DiD), 1,1'-dioctadecyl-3,3,3',3'-tetramethylindotricarbocyanine iodide (purity 95.0%, DiR), and L-*α*-phosphatidic acid (sodium salt, purity 98%, from soybean) were acquired from Shanghai Aladdin Bio Chem Technology Co., Ltd. Alexa Fluor 488-labeled anti-CD68 antibody (Cat# 137011) and PE-labeled anti-CD146 antibody (Cat# 134704) were obtained from BioLegend, Inc. Anti-*α*-SMA antibody (Cat# 14-9760-82), PE-cyanine7-labeled anti-mouse IgG2a secondary antibody (Cat# 25-4210-82), Dulbecco's modified Eagle's medium (DMEM), fetal bovine serum (FBS), and Roswell Park memorial institute 1640 medium (RPMI 1640) were purchased from Thermo Fisher. FITC-labeled anti-F4/80 antibody (Cat# 52267), PE-cyanine7-labeled anti-CD19 antibody (Cat# 27221), and APC-labeled anti-CD11c antibody (Cat# #33293) were from Cell Signaling Technology, Inc. Rabbit monoclonal [EPR19394] to C3 (ab200999), goat anti-mouse IgM mu chain (HRP) (ab97230), and rabbit monoclonal [RIGG-69] to IgG (ab133470) were provided by Abcam. Complement C3 proteins (Cat# HY-P78247 for mouse and Cat# HY-P7862 for human) were from MedChemExpress. Mouse IgM protein (Cat# B1120) was purchased from Bersee Technology Co. Immunoglobulin G (IgG) ELISA Kits (JL12948 for mouse and JL12687 for human), immunoglobulin M (IgM) ELISA Kits (JL12937 for mouse and JL10665 for human), and immunoglobulin A (IgA) ELISA kits (JL11756 for mouse and JL12695 for human) were purchased from Jianglai biology. Complement C3a ELISA kits (EM0882 for mouse) and complement C5a ELISA kits (EM0885 for mouse) were acquired from Wuhan Fine Biotech Co., Ltd. Aspartate aminotransferase Assay Kit (C010-2-1), Alanine aminotransferase Assay Kit (C009-2-1), Urea Assay Kit (C013-2-1), and Creatinine Assay kit (C011-2-1) were purchased from Nanjing Jiancheng Bioengineering Institute (Nanjing, China). Annexin V-FITC/PI apoptosis detection kits (KGA1101) were purchased from KeyGEN BioTECH. All chemicals and materials were used as received. Mouse serum was collected from orthotopic 4T1 tumor-bearing mice and healthy mice and stored at −80 °C. Human serum was obtained from the Qingdao municipal hospital (China) from breast cancer patients and healthy donors pooled and stored at -80 °C, in accordance with the Declaration of Helsinki. Prior to use, human serum was centrifuged at 20 000 g for 30 min to remove protein aggregates. All individuals and/or their parents provided informed consent to participate in this study and approval was provided by Specialized Committee on Science Ethics of the Academic Council of Ocean University of China (OUC-HM-2024-115-010).

**1.2 Cell culture and animal care**

Human breast cancer cell line MCF-7 (RRID: CVCL_0031), mouse breast cancer cell line 4T1 (RRID: CVCL_0125), human monocytic leukaemia cell line THP-1 (RRID:CVCL_0006), mouse monocyte macrophage leukaemia cell line RAW264.7 (RRID: CVCL_0493), mouse fibroblast cell line L929 (RRID: CVCL_4238), and human umbilical vein endothelial cell line HUVEC (RRID: CVCL_9Q53) were obtained from the Type Culture Collection Committee of the Chinese Academy of Sciences. The cell lines were verified through morphological assessment and PCR-based approaches to confirm their identities and ensure they were free of mycoplasma contamination. The cells were cultured in DMEM or RPMI 1640 supplemented with 10% FBS, 100 U mL^−1^ penicillin, and 100 *µ*g mL^−1^ streptomycin.

Female BALB/c mice (6 weeks old, 16-18 g) and female Sprague-Dawley rats (6 weeks old, 210 g) were purchased from Pengyue Experimental Animal Breeding Co. Ltd. (Jinan, China). The animals were maintained on a standard diet and water ad libitum at 22 °C and 50%~60% relative humidity. All animal experiments were performed in accordance with the Institutional Animal Care and Use guidelines and the National Research Council’s Guide. Animal experiments were approved by the Ethics and Animal Welfare Committee of the Ocean University of China (Application No. OUC-SMP-2022-11-08, OUC-SMP-2023-04-09).

**1.3 Synthesis and characterization of** **phosphatidyl saccharides**

Bacillus subtilis was used as protein expression carrier. We activated the secreted expression strain, previously produced in the laboratory, and cultured it in LB medium for 12 h. Subsequently, phospholipase D (PLD) enzyme powder was prepared through freeze-drying. Due to the abundance of hydroxyl groups in the saccharide molecules, PLD can combine them with phosphate groups of phospholipids (*e.g.*, phosphatidylcholine) to catalyze the synthesis of phosphatidyl saccharides. Transphosphatidylation reactions between phosphatidylcholine (PC) and various saccharides were efficiently catalyzed by PLD in a dual-phase reaction system comprising an organic solvent and a buffer. Specifically, 10 mg of PC was dissolved in 1 mL of ethyl butyrate, while 1.0 U of PLD and the saccharides (galactose, glucose, *N*-acetylamino-D-glucose, mannose, or fructose) were dissolved in 1 mL of sodium citrate buffer (pH 6.0). Afterwards, the two phases were added in the reactor to initiate the reaction. The reaction was continued under stirring at 40 °C for 12 h. Finally, the resulting organic phase was carefully separated by centrifugation at 1800 g for 5 min, and dried by rotary evaporation to evaporate the organic solvent. The obtained mixture was stored at -20 °C as final product. The structures of synthesized phosphatidyl saccharides were characterized using ^1^H-NMR spectroscopy (Agilent ProPulse, USA), high-resolution mass spectrometry and tandem mass spectrometry (MS/MS) (Thermo Scientific Q Exactive Orbitrap MS, USA), and attenuated total reflectance-Fourier transform infrared spectroscopy (ATR-FTIR, Thermo Scientific Nicolet iS50 FTIR, USA). The reaction yield of phosphatidyl saccharides were determined using high-performance liquid chromatography coupled with an evaporative light-scattering detector (HPLC-ELSD, SHIMADZU Essentia ELSD-16, Japan) and calculated based on the peak area of each component using following equation (1):

$Reaction yield \left( \% \right)=\frac{C_{Phosphatidyl Saccharides}}{C_{PC+PA+Phosphatidyl Saccharides}}\times100\%$ (1)

Where *C_Phosphatidyl Saccharides_* represents the concentration of synthesized phosphatidyl saccharides, while *C_PC+PA+Phosphatidyl Saccharides_* refers to the total concentration of substrate PC, by-product PA, and product phosphatidyl saccharides in the mixture.

**1.4 Preparation and characterization of glycosylated liposomal nanoparticles**

Glycosylated liposomal nanoparticles (G-LNPs) were prepared using a thin-film dispersion-sonication method by incorporating phosphatidyl saccharides into the lipid bilayers. The obtained liposomal nanoparticles (LNPs) were denoted as PC-LNPs (unmodified), PA-LNPs (unmodified, a charge-matched control), Gal-LNPs, Glc-LNPs, GlcNAc-LNPs, Man-LNPs, and Fru-LNPs. For blank G-LNPs (without dye or drug loading), a mixture of phospholipid/cholesterol (3:1 molar ratio) in ethyl butyrate was rotary evaporated to form a uniform and transparent film. The added molar percentages of phosphatidyl saccharides (10 mg mL^-1^), L-*α*-phosphatidylcholine (10 mg mL^-1^), and cholesterol (10 mg mL^-1^) were 37.5%, 37.5%, and 25%, respectively. Residual organic solvent was removed by overnight vacuum evaporation. The dried lipid film was subsequently hydrated with 2 mL aqueous phase (PBS, 0.1 M, pH = 7.4) at 45 °C for 20 min, and the resulting lipid dispersion was sonicated (300 W, 2 s on, 1 s off) for 10 min to obtain the G-LNPs solution. For Fru-LNPs and GlcNAc-LNPs with varying molar percentages of phosphatidyl saccharides (0% to 65%), the same formulation and preparation process were carried out, except for the adjustment of molar percentages of phosphatidyl saccharides within the total phospholipids. Fluorescence dye (including DiI, DiD, and DiR, 1 mg mL^-1^ in anhydrous ethanol)-labeled liposomal nanoparticles (DiI/DiD/DiR@G-LNPs) were prepared using the same procedure except that the corresponding fluorescent probe (50 *μ*L) was added to the organic phase. The added molar percentage of fluorescence dye was 0.37%. Doxorubicin-loaded glycosylated liposomal nanoparticles (DOX@G-LNPs) were prepared using a traditional ammonium sulfate gradient loading method,^[1, 2]^ where the LNPs were incubated with a doxorubicin solution (10 mg mL^-1^) at the lipid/DOX molar ratio of 10:1 at 45 °C for 1 h. After cooling to room temperature, free DOX was removed by dialysis.

The particle size, PDI, and zeta potential of LNPs were measured by a Zetasizer (Nano S90, Malvern Panalytical GmbH, Germany). Morphology of LNPs was characterized using a transmission electron microscope (TEM) (Tecnai G2 F20 S-Twin, FEI, Hillsboro, OR, USA). For transmission electron microscopy (TEM) analysis, sample preparation was conducted as follows. A dilute suspension of the liposomal nanoparticles (0.1 mg mL^-1^) was prepared to minimize particle aggregation. Subsequently, a 20 *µ*L aliquot of this suspension was deposited onto a carbon-coated copper grid and incubated for 15 min. Excess liquid was then carefully removed by blotting with filter paper. To enhance contrast, the samples were negatively stained with a 1% (w/v) uranyl acetate solution. Finally, the prepared grids were allowed to air-dry completely at room temperature prior to imaging. TEM micrographs were acquired from multiple, widely separated grid squares to ensure statistically representative sampling. TEM images were further subjected for statistical size analysis using Nano Measurer software 1.2. For each type of G-LNPs, at least 50 individual nanoparticles were randomly selected and measured. The resulting size distributions were statistically analyzed using one-way ANOVA to evaluate differences among the various G-LNP formulations. Encapsulation efficiency (EE) of DOX in G-LNPs was measured by using fluorescence spectrometry (*λ*_ex_ = 495 nm, *λ*_em_ = 595 nm) and calculated based on the equation (2):

$EE\left( \% \right)=\frac{m_{DOX detected}}{m_{DOX input}}\times100\%$ (2)

where *m_DOX detected_* represents the mass of DOX in DOX@G-LNPs samples calculated from the standard curve, whereas *m_DOX input_* refers to the mass of DOX input.

**1.5 Preliminary safety evaluation**

To evaluate the preliminary safety of G-LNPs, a hemolysis test was conducted. Erythrocytes isolated from sterile defibrinated sheep blood were washed 3 times with normal saline to prepare 2% (*v/v*) erythrocyte suspensions. Various concentrations of G-LNPs (from 2.5 *μ*g mL^-1^ to 100 *μ*g mL^-1^) were incubated with 2% erythrocyte suspensions at 37 °C for 1 h, with ultrapure water and normal saline as positive and negative controls, respectively. After incubation, the red blood cell suspensions were centrifuged at 300 g for 10 min, and the absorption of supernatants were measured at 540 nm using UV spectroscopy. The hemolysis rate was calculated using the following equation (3):

$Hemolysis rate \left( \% \right)=\frac{{Abs}_{sample} - {Abs}_{negative}}{{Abs}_{positive} - {Abs}_{negative}} \times100\%$ (3)

where *Abs_sample_* represents the absorbance of supernatants of the G-LNPs groups, whereas *Abs_positive_* and *Abs_negative_* represent the absorbance of supernatants of the ultrapure water and normal saline-treated groups, respectively.

To assess the cytotoxicity of G-LNPs on normal cells, a cell viability assay was performed. Three cell types (HUVEC, RAW264.7, and L929) were seeded in 96-well plate at a density of 1 × 10^4^ cells per well and cultured overnight. After that, complete medium containing the same concentration of G-LNPs as used in the hemolysis test were added to replace the old medium, and incubation was continued for 48 h. Cell viability was measured by using a CCK8 kit and a multifunctional plate reader (Tecan Spark, Switzerland).

To evaluate the potential hepatic and renal toxicity of G-LNPs, serum levels of liver enzymes (aspartate aminotransferase, AST; alanine aminotransferase, ALT) and kidney function markers (blood urea nitrogen, BUN; serum creatinine, Scr) were measured. 200 *μ*L blank G-LNPs (1 mg mL^-1^) was injected into female BALB/c mice (6 weeks old, 16-18 g) *via* the tail vein. At 48 h post-injection, blood was collected *via* retro-orbital bleeding under anesthesia, and serum was separated for analysis. Blood biochemical indicators (AST, ALT, BUN, and Scr) were measured using the corresponding Assay Kit with a multifunctional plate reader (Tecan Spark, Switzerland).

**1.6 Uptake of G-LNPs by 4T1 and MCF-7 tumor cells**

4T1 and MCF-7 cells were seeded separately in a 6-well plate at a density of 5×10^5^ cells per well. After overnight culturing, the old medium was replaced with complete medium containing the same concentration of DiI@G-LNPs, and continued to incubate for 2 h. The fluorescence intensity of the cells was then measured using a flow cytometer (Beckman Coulter Gallios, USA). Identically, images of 4T1 or MCF-7 cells subjected to the same dosing schedule as flow cytometry were captured using a CLSM. To investigate time-, concentration-, and temperature-dependent uptake, the same experimental procedures were followed. For competitive uptake studies, cells were pre-incubated with the corresponding saccharide molecules for 1 h before co-culturing with G-LNPs. In addition, the same experimental procedure was applied to assess the uptake of DOX@G-LNPs (PC-LNPs, GlcNAc-LNPs, Fru-LNPs, and Free DOX as control) by 4T1 or MCF-7 cells.

**1.7 Construction of** **orthotopic 4T1 tumor-bearing mice model**

Orthotopic 4T1 tumor-bearing mice model was established to evaluate the *in vivo* biodistribution and antitumor efficacy of G-LNPs. Briefly, mouse breast cancer 4T1 cells (approximately 1 × 10^5^ cells suspended in 100 *μ*L PBS) were inoculated under mammary fat pads of female BALB/c mice (6 weeks, 16-18 g), and tumor volume was monitored.

**1.8 Uptake of G-LNPs by RAW264.7 and THP-1 cells**

RAW264.7 cells were cultured in DMEM medium containing 10% FBS with 5% CO_2_ at 37 °C. Cells were seeded in 6-well plates at a density of 5 × 10^5^ cells per well and incubated overnight. FBS was then removed, and DiI@G-LNPs pre-incubated with sera from either healthy mice or orthotopic 4T1 tumor-bearing mice at 37 °C for 1 h were added. After additional 2 h co-incubation, cells were harvested and analyzed by flow cytometry to assess differences in macrophage phagocytosis of various G-LNPs. Moreover, the impact of species differences in serum on macrophage uptake was investigated by substituting mice sera with human sera from individuals with identical health/disease states. THP1 cells underwent the same procedure, except that the human sera from either healthy donors or breast cancer patients were used.

To explore the correlation between molar percentages of glycosyl ligands and macrophage uptake, RAW264.7 cells were incubated with Fru-LNPs or GlcNAc-LNPs containing varying molar percentages of phosphatidyl saccharides (0% to 65%).

**1.9 Uptake of G-LNPs by BMDCs**

Bone marrow cells were isolated from BALB/c mice, and cultured in RMPI 1640 medium supplemented with FBS (10%), GM-CSF (20 ng mL^-1^), and IL-4 (20 ng mL^-1^) at 37 °C for 7 days.^[3]^ Once the percentage of CD11c^+^ cells exceeded 70%, as determined by flow cytometry, the bone marrow dendritic cells (BMDCs) were considered ready for subsequent experiments. For investigating the uptake of DiI@G-LNPs, DiI@G-LNPs pre-treated with mice sera form different health/cancer states at 37 °C for 1 h were co-incubated with BMDCs for 2 h. Cells were then harvested and the proportion of DiI^+^ cells was quantified by flow cytometry.

**1.10 Cytotoxicity of DOX@G-LNPs**

4T1 or MCF-7 cells (1 × 10^4^ cells per well) were seeded in 96-well plate in RMPI 1640 complete medium and cultured overnight. Free DOX, DOX@PC-LNPs, DOX@GlcNAc-LNPs, and DOX@Fru-LNPs were added to the cells at varying DOX concentrations (0.0125, 0.025, 0.05, 0.1, 0.25, 0.5, 1.0, and 2.5 *μ*g mL^-1^). After 48 h of incubation, cell viability was assessed using CCK-8 kit and calculated with the following equation (5):

$Cell viability \left( \% \right)=\frac{{OD}_{treated}}{{OD}_{control}} \times100\%$ (4)

**1.11 Apoptosis of 4T1 and MCF-7 tumor cells induced by DOX@G-LNPs**

Annexin V-FITC/PI apoptosis detection kit was used to detect cell apoptosis induced by DOX@ G-LNPs (DOX@PC-LNPs, DOX@GlcNAc-LNPs, or DOX@Fru-LNPs). 4T1 and MCF-7 cells in the exponential growth stage were seeded in 6-well plates at a density of 1 × 10^6^ cells per well and cultured overnight. The culture medium was then removed, and the same treatment conditions as used in the cytotoxicity assay were applied, with DOX concentrations of 0.25 *μ*g mL^-1^ for both the free DOX and DOX@G-LNPs groups. After treatment, all cells were collected to detect the apoptosis by using flow cytometry.

**1.12 Analysis of main cell populations for G-LNPs clearance in blood** **using flow cytometry**

To analyze the blood cell populations responsible for G-LNPs clearance, uptake of DiD@G-LNPs in different blood cell types was detected using flow cytometry. 200 *μ*L DiD@G-LNPs was injected into tumor-bearing mice *via* the tail vein. At 0.5 h post-injection, mice were anesthetized, and peripheral blood was collected *via* retro-orbital bleeding. Monocyte, neutrophil, and lymphocyte were isolated from the blood using Mouse Monocyte Extraction Kit, Mouse Neutrophil extraction kit, and Mouse Lymphocyte extraction kit. Then, the ratio of DiD^+^ cells in blood cells was determined by using flow cytometry.

**1.13 Analysis of main cell populations for G-LNPs clearance in liver and spleen** **using flow cytometry**

To analyze the major cell populations responsible for G-LNPs clearance in the liver, uptake of DiD@G-LNPs in different liver cell types was detected using flow cytometry. 200 *μ*L of DiD@G-LNPs was injected into tumor-bearing mice *via* the tail vein. At 2, 12, and 24 h post-injection, hepatic parenchymal cells (hepatocytes) and hepatic non-parenchymal cells (Kupffer cells, endothelial cells, and hepatic stellate cells) were isolated using a 2-step collagenase perfusion centrifugation method as described previously^[4]^. Mice were anaesthetized with isoflurane, and the inferior vena cava and portal vein were surgically exposed. The inferior vena cava was cannulated and the portal vein was severed. A pre-warmed HBSS solution (lacking Ca^2+^ and Mg^2+^ and containing 0.5 × 10^-3^ M EDTA and 25 × 10^-3^ M HEPES) was then continuously perfused for 5-10 min. Liver digestion buffer (200 units of collagenase type IV and 25 × 10^-3^ M HEPES in HBSS buffer containing Ca^2+^ and Mg^2+^) was then perfused for 10-20 min. The digested liver was collected, chopped, and plated in liver digestion buffer. HBSS solution (10 mL) was added, and low-speed centrifugation at 50 g for 3 min at 4 °C was used to separate hepatocytes (pellet) and nonparenchymal cells (Kupffer cells, endothelial cells, and hepatic stellate cells; supernatant). The liver non-parenchymal cells were stained with Alexa Fluor 488-labeled anti-CD68 antibody (for Kupffer cells), PE-labeled anti-CD146 antibody (for endothelial cells), and anti-*α*-SMA antibody (for hepatic stellate cells). The ratios of DiD^+^ cells in hepatocytes and nonparenchymal cells were determined by flow cytometry.

To analyze the primary splenic antigen-presenting cell types responsible for clearing G-LNPs, the same injection protocol (200 *μ*L DiI@G-LNPs per mouse) was followed. Mice were euthanized, and their spleens were collected, minced, and processed into single-cell suspensions. The spleen cell suspension was treated with erythrocyte lysate to remove erythrocytes. After that, the spleen cell suspension was stained with FITC-labeled anti-F4/80 antibody (for macrophages), PE-cyanine7-labeled anti-CD19 antibody (for B cells), and APC-labeled anti-CD11c antibody (for dendritic cells). The ratio of DiI^+^ cells in splenic antigen presenting cells was determined by using flow cytometry.

**1.14 Isolation and characterization of protein corona**

BALB/c mice were intravenously injected with 200 *μ*L of DiR@G-LNPs (50 mg phospholipids per kg) *via* the tail vein and anesthetized at 1 h. Blood was sampled and the serum was collected after centrifugation at 1000 g for 10 min. The liposomal nanoparticle-protein complex was collected by centrifuging at 14,000 g for 1 h. The pellet was then resuspended in 2 wt% SDS with 62.5 mM Tris-HCl, incubated at 95 °C for 5 min, and centrifuged again (14,000 g, 1 h, 4 °C). Finally, the supernatant containing desorbed corona proteins was analyzed using SDS-PAGE, BCA Protein Assay Kit, and LC-MS/MS.

**1.15 LC-MS/MS analysis**

The samples were analyzed using an Vanquish Neo nano system coupled online with Q Exactive HF mass spectrometer through a Nano spray Flexion source (Thermo Scientific). The obtained peptide samples were injected into a C18 trap column and separated in a reversed-phase C18 column. The separation gradient consisted of mobile phase A (0.1% formic acid aqueous H_2_O) and mobile phase B (0.1% formic acid and 80% acetonitrile) at a flow rate of 300 nL min^-1^. Mass spectrometry data collection was performed in DDA mode with MS1 scan resolution set as 60 K@200 m/z and a sweep range of 350-1500 m/z. The parameters of MS2 scan are set as follows: a resolution of 15K@200 M/z and an AGC target of 1E5. The time interval for dynamic elimination was 30 s.

**1.16 Quantitative analysis of C3/IgG deposition on G-LNPs surface using western blot**

To elucidate the correlation between the amount of adsorbed C3/IgG and glycosyl ligands, these proteins deposited on the surface of G-LNPs were quantitatively determined by using western blot. The same protocol was used to isolate the mice protein coronas. The obtained protein corona pellets were resuspended in a mixed solution (50 *μ*L) containing 1 *μ*g SDS, 1.2 *μ*L 1.5 M Tris-HCl, and 10 *μ*L SDS-PAGE (5x) loading buffer. The samples were boiled at 100 °C for 10 min and then separated by SDS-PAGE. In addition, after transferring the separated gels to nitrocellulose membranes, antibodies to mouse C3 or IgG were respectively added to detect corresponding proteins followed by enhanced chemiluminescence.

**1.17 Quantitative analysis of immunoglobulins in protein corona using ELISA**

To further elucidate the composition of immunoglobulins in the protein corona, the relative amounts of the three major immunoglobulins (IgG, IgM, and IgA) were investigated by ELISA measurements. The desorbed protein coronas, prepared from serum of mice or human, were isolated by utilizing the aforementioned procedures. The concentration of each immunoglobulin (IgG, IgM, or IgA) was determined respectively using the IgG, IgM, or IgA ELISA kits according to the manufacturer’s instructions.

**1.18 *In vitro* evaluation of effect of C3/IgG/IgM deposition on macrophage uptake of G-LNPs**

50 *μ*L of DiI@G-LNPs was incubated with 50 *μ*L of protein solution (C3: 10 *μ*g mL^-1^, IgG: 20 *μ*g *μ*L^-1^, or IgM: 2 *μ*g *μ*L^-1^) at 37 °C for 1 h, with 50 *μ*L of PBS serving as a control. Next, these G-LNPs were diluted 10-fold and then cultured with RAW264.7 cells for 2 h. After that, the cells were collected and subjected to flow cytometry for cell uptake analysis.

**1.19 *In vitro* C3a/C5a determination using ELISA**

To investigate the effect of G-LNPs on the complement system, the levels of complement activation products C3a and C5a were detected by ELISA measurements. Specifically, 200 *μ*L of G-LNPs were incubated with 200 *μ*L of different sera from mice at 37 °C for 1 h. PBS was used as negative control while Zymosan was set as positive control. The complement activation reaction was terminated by adding a cold EDTA solution (60 mM in PBS). Next, the mixture was centrifuged at 14,000 g for 1 h and the C3a or C5a in supernatant was measured respectively using the C3a or C5a ELISA kits according to the manufacturer’s instructions.

**1.20 *In vivo* biodistribution imaging of G-LNPs**

The *in vivo* fluorescence distribution of DiR@G-LNPs was investigated in an orthotopic 4T1 tumor-bearing mice model using the IVIS Spectrum CT imager (PerkinElmer, Waltham, USA). Tumor-bearing mice (tumor volume was up to about 300 mm^3^) was intravenously injected with DiR@G-LNPs at the dose of 5 mg phospholipids per kg. After 24 h, mice were imaged to observe the biodistribution of G-LNPs *in vivo*. Subsequently, the mice were euthanized, and the major organs (brain, heart, liver, spleen, lung, and kidney) and tumor tissue were collected for imaging and their fluorescence intensities were recorded.

To further examine the influence of glycosyl ligand density on organ-level distribution, DiR@Fru-LNPs and DiR@GlcNAc-LNPs with varying phosphatidyl saccharide contents (0-65 mol%) were intravenously administrated to tumor-bearing mice. At 2 h post-injection, mice were euthanized, and major organs (liver and spleen) as well as tumor tissues were collected for *ex vivo* fluorescence imaging to assess the biodistribution of G-LNPs.

*Ex vivo* imaging of DiR@G-LNPs in major organs and tumor was performed using an IVIS Spectrum system (PerkinElmer). All organs were collected at indicated time points, rinsed with PBS, and imaged under identical acquisition parameters (excitation/emission: 740 nm/790 nm; exposure time: 2 s; binning: 4*; f/stop: 2; field of view: 12.5 cm). For quantification, regions of interest (ROIs) were drawn to cover each entire organ, and the total radiant efficiency ([p s^-1^] [*μ*W cm^-2^]^-1^) was calculated using Living Image software. Background signals were subtracted using ROIs from non-tissue areas and tissues from non-injected control mice. Data are presented as mean ± SD from at least three mice per group.

**1.21 Pharmacokinetics study**

To investigate the pharmacokinetic profile of G-LNPs, SD rats (*n* = 6) were intravenously administered with various DiR@G-LNPs (2.5 mg phospholipids per kg). Blood was sampled at 0 min, 5 min, 15 min, 30 min, 1 h, 2 h, 4 h, 8 h, 12 h, and 24 h after injection. Plasma was separated by centrifugation at 1000 g for 15 min. The plasma concentration of DiR was measured by a fluorescence spectrophotometer (*λ*_ex_ = 740 nm, *λ*_em_ = 780 nm).

**1.22 *In vivo* anti-tumor efficacy**

The *in vivo* antitumor efficacy of DOX@G-LNPs was evaluated using an orthotopic 4T1 tumor-bearing mice model. Once the tumor size reached ~ 50 mm^3^, the mice were randomly divided into 5 groups (*n* = 5), including: PBS (negative control), Free DOX (positive control), DOX@PC-LNPs, DOX@GlcNAc-LNPs, and DOX@Fru-LNPs. The first administration was designated as day 0, and a dose of 2 mg kg^-1^ was administered *via* tail vein injection at 2-day intervals (on days 0, 2, 4, 6, 8, 10, 12, 14, and 16). At the same time, tumor volumes and body weights were recorded every 2 days. At the end of experiment (day 18), the mice were euthanized, and tumors were extracted and weighed to calculate the inhibition rate. Tumor tissue was subjected to hematoxylin and eosin (H&E), TUNEL, and Ki67 staining. The major organs (heart, lung, spleen, kidney, and liver) were removed, weighed, and subjected to pathological sections, and peripheral blood was used for routine blood tests and liver and kidney safety evaluation.

**1.23 Statistical analysis**

Statistical analysis was performed using SPSS 21.0 software. A two-sided Student's *t*-test (*α = 0.05*) was used for comparisons between two different conditions, while ANOVA was used for comparing multiple groups. Data are presented as mean ± standard deviation (SD) and **p < 0.05* was considered statistically significant.

**2. Results**


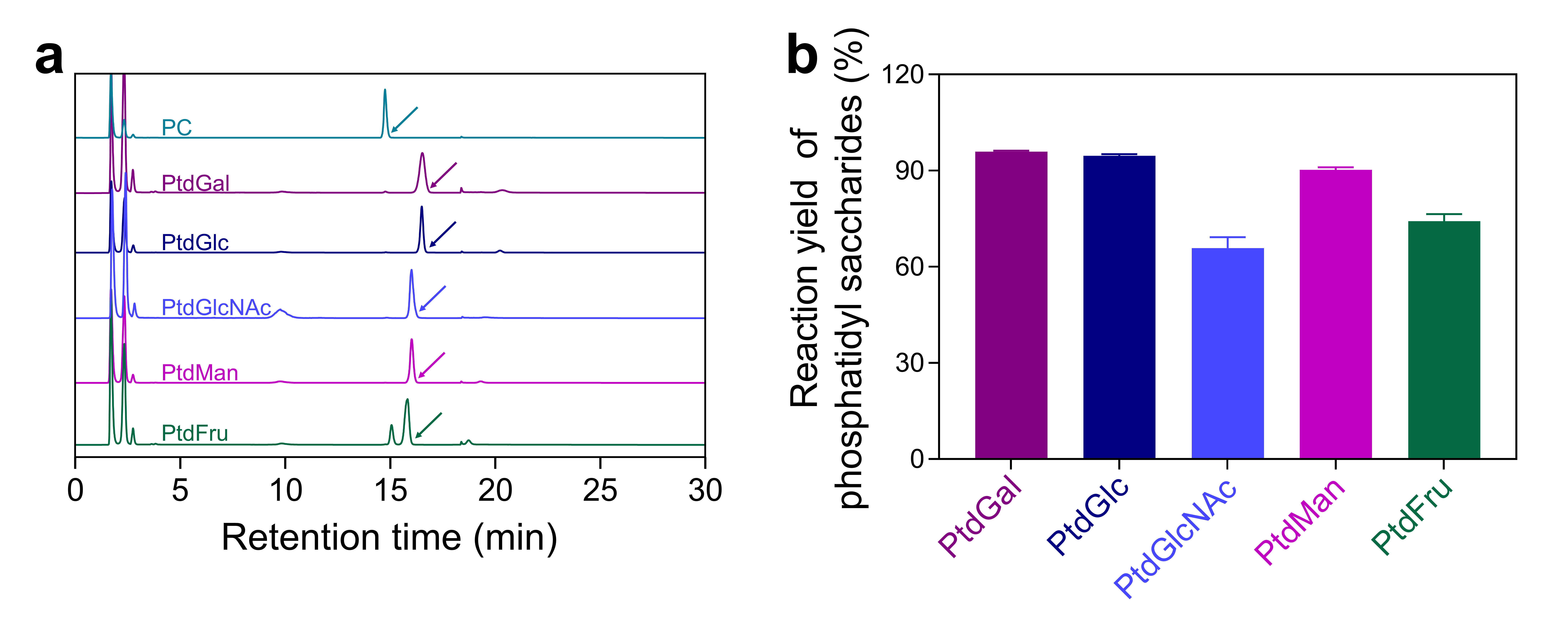


**Figure S1.** HPLC-ELSD of phosphatidyl saccharides synthesized by PLD. (a) Retention time. (b) Reaction yield. Data are presented as mean ± SD (*n* = 3).


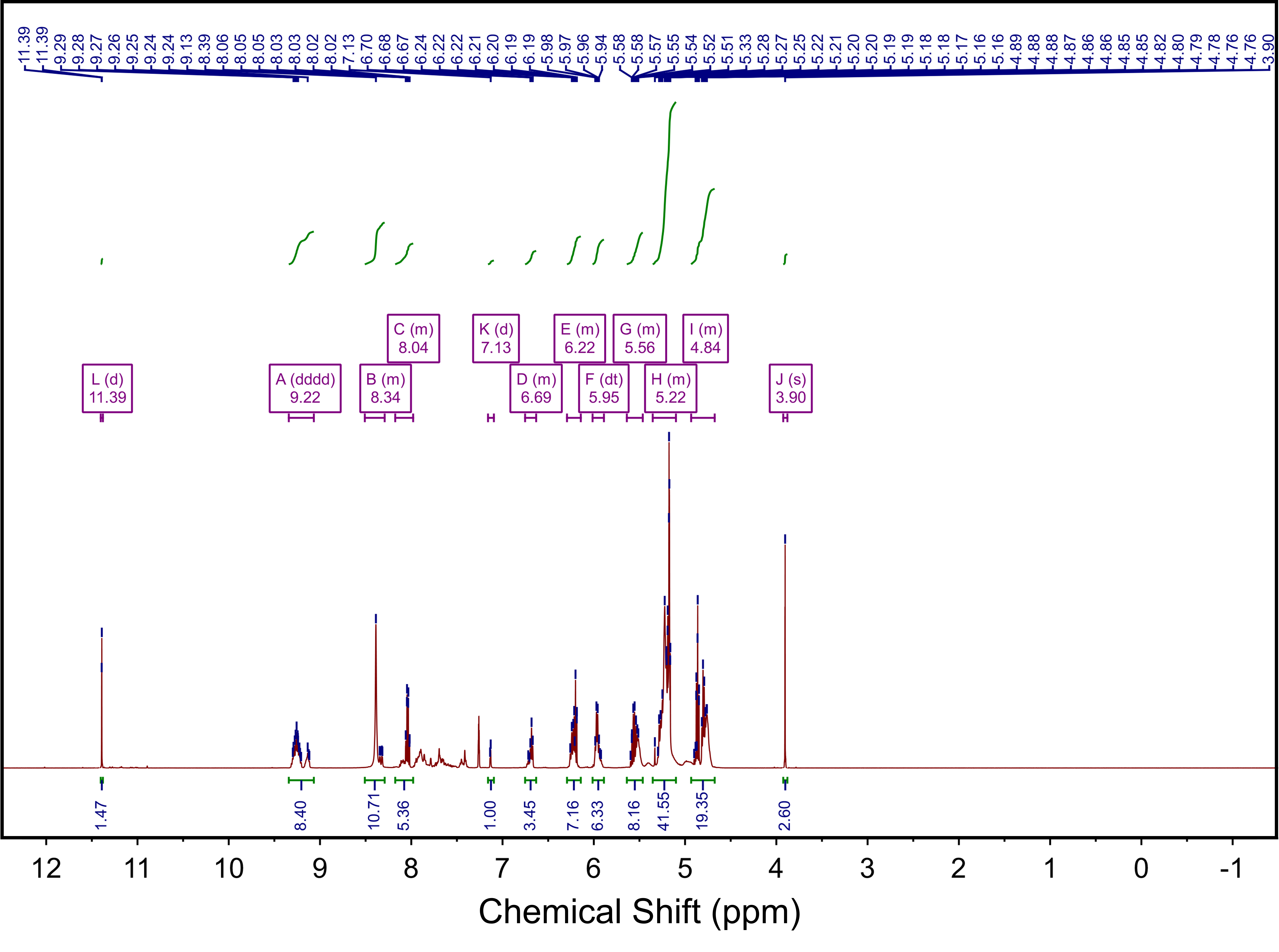


**Figure S2.** ^1^H-NMR spectrum (500 MHz, CDCl_3_: CD_4_O = 2:1) of synthesized PtdGal.

**PtdGal:** ^1^H NMR (500 MHz, cdcl3) δ 11.39 (s, 1H), 9.21 (dddd, J = 41.9, 38.5, 23.2, 4.3 Hz, 5H), 8.39 (s, 7H), 8.15 – 8.00 (m, 3H), 7.13 (t, J = 5.4 Hz, 1H), 6.69 (dd, J = 15.7, 9.0 Hz, 2H), 6.28 – 6.15 (m, 4H), 5.95 (dt, J = 11.2, 6.2 Hz, 4H), 5.64 – 5.45 (m, 5H), 5.37 – 5.08 (m, 26H), 4.93 – 4.67 (m, 11H), 3.90 (s, 2H).


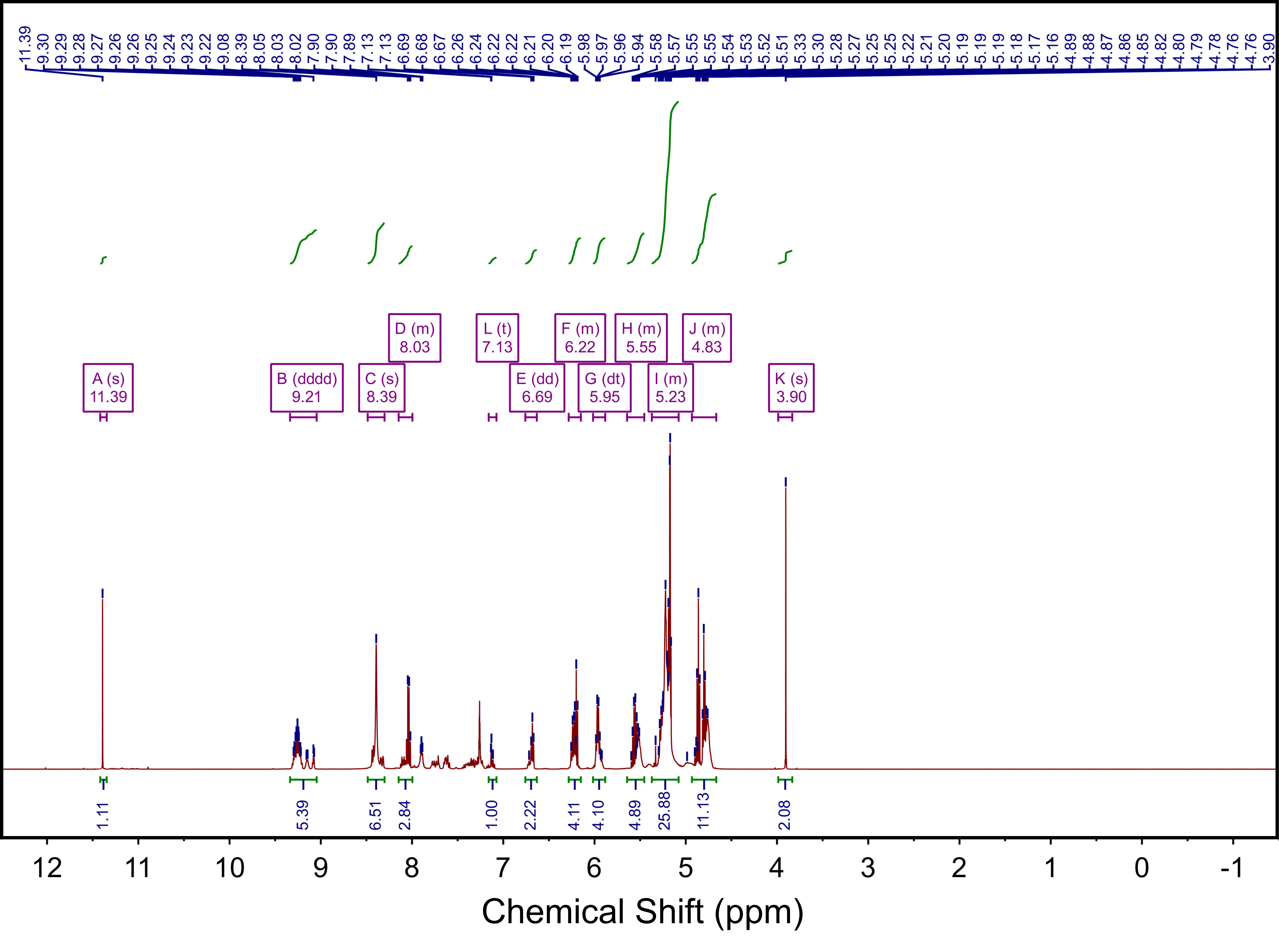


**Figure S3.** ^1^H-NMR spectrum (500 MHz, CDCl_3_: CD_4_O = 2:1) of synthesized PtdGlc.

**PtdGlc:** ^1^H NMR (500 MHz, cdcl3) δ 11.39 (s, 1H), 9.21 (dddd, J = 41.9, 38.5, 23.2, 4.3 Hz, 5H), 8.39 (s, 7H), 8.15 – 8.00 (m, 3H), 7.13 (t, J = 5.4 Hz, 1H), 6.69 (dd, J = 15.7, 9.0 Hz, 2H), 6.28 – 6.15 (m, 4H), 5.95 (dt, J = 11.2, 6.2 Hz, 4H), 5.64 – 5.45 (m, 5H), 5.37 – 5.08 (m, 26H), 4.93 – 4.67 (m, 11H), 3.90 (s, 2H).


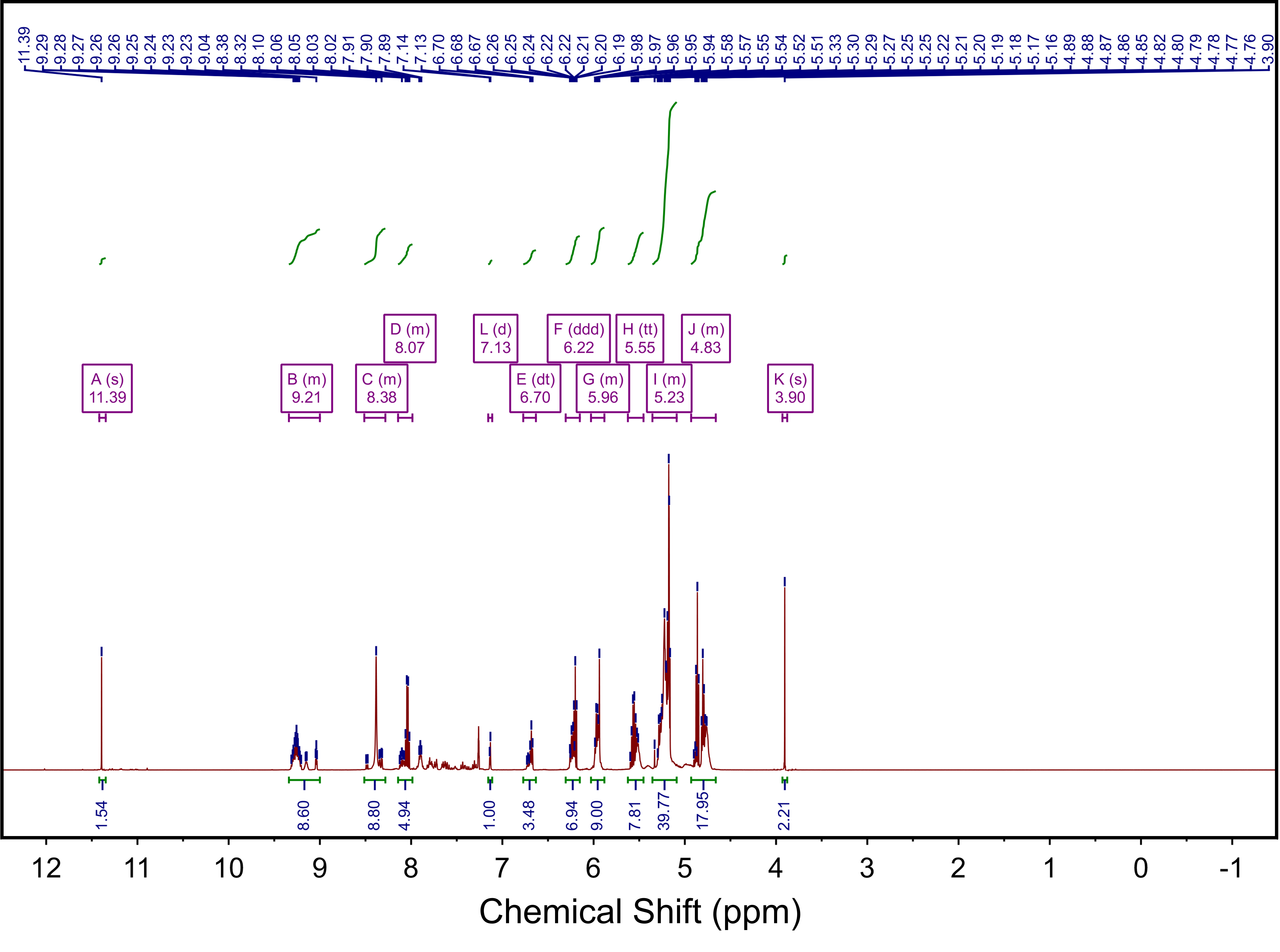


**Figure S4.** ^1^H-NMR spectrum (500 MHz, CDCl_3_: CD_4_O = 2:1) of synthesized PtdGlcNAc.

**PtdGlcNAc:** ^1^H NMR (500 MHz, cdcl3) δ 11.39 (s, 2H), 9.34 – 9.00 (m, 9H), 8.51 – 8.28 (m, 9H), 8.14 – 7.99 (m, 5H), 7.13 (d, J = 4.0 Hz, 1H), 6.70 (dt, J = 13.3, 6.4 Hz, 3H), 6.22 (ddd, J = 14.8, 10.4, 4.9 Hz, 7H), 6.03 – 5.88 (m, 9H), 5.55 (tt, J = 13.6, 6.8 Hz, 8H), 5.36 – 5.09 (m, 40H), 4.93 – 4.66 (m, 18H), 3.90 (s, 2H).


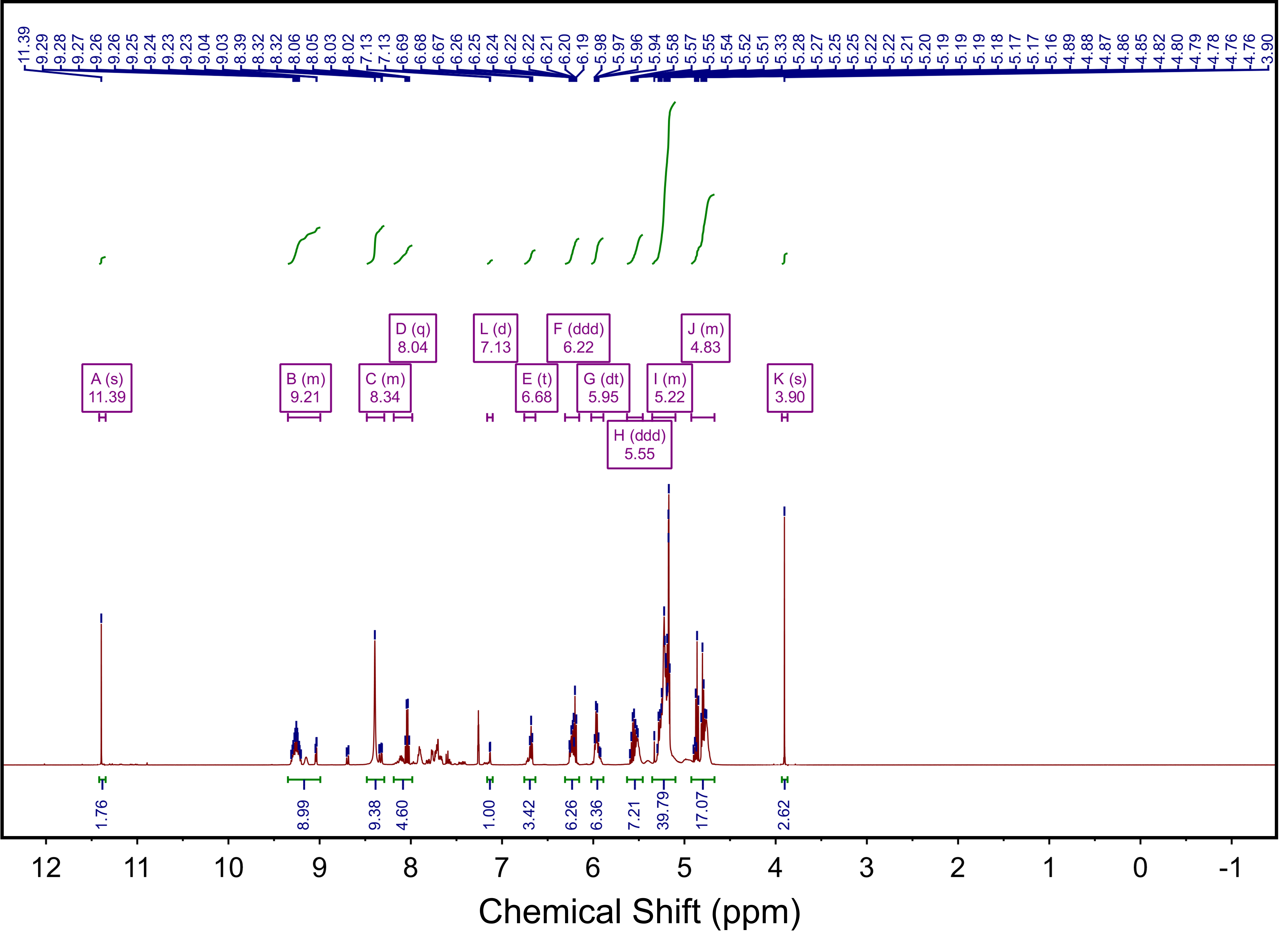


**Figure S5.** ^1^H-NMR spectrum (500 MHz, CDCl_3_: CD_4_O = 2:1) of synthesized PtdMan.

**PtdMan:** ^1^H NMR (500 MHz, cdcl3) δ 11.39 (s, 1H), 9.21 (dddd, J = 41.9, 38.5, 23.2, 4.3 Hz, 5H), 8.39 (s, 7H), 8.15 – 8.00 (m, 3H), 7.13 (t, J = 5.4 Hz, 1H), 6.69 (dd, J = 15.7, 9.0 Hz, 2H), 6.28 – 6.15 (m, 4H), 5.95 (dt, J = 11.2, 6.2 Hz, 4H), 5.64 – 5.45 (m, 5H), 5.37 – 5.08 (m, 26H), 4.93 – 4.67 (m, 11H), 3.90 (s, 2H).


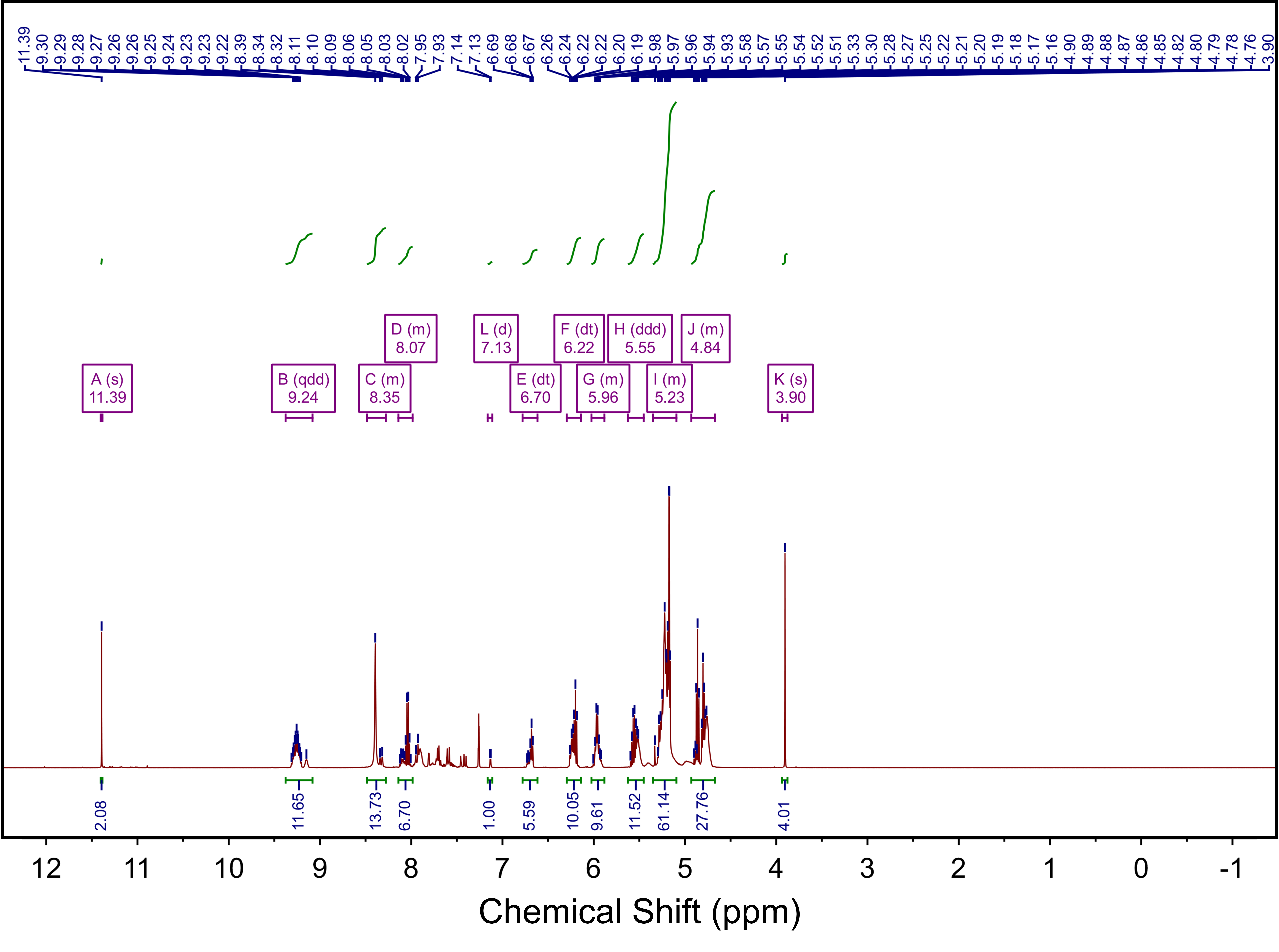


**Figure S6.** ^1^H-NMR spectrum (500 MHz, CDCl_3_: CD_4_O = 2:1) of synthesized PtdFru.

**PtdFru:** ^1^H NMR (500 MHz, cdcl3) δ 11.39 (s, 1H), 9.21 (dddd, J = 41.9, 38.5, 23.2, 4.3 Hz, 5H), 8.39 (s, 7H), 8.15 – 8.00 (m, 3H), 7.13 (t, J = 5.4 Hz, 1H), 6.69 (dd, J = 15.7, 9.0 Hz, 2H), 6.28 – 6.15 (m, 4H), 5.95 (dt, J = 11.2, 6.2 Hz, 4H), 5.64 – 5.45 (m, 5H), 5.37 – 5.08 (m, 26H), 4.93 – 4.67 (m, 11H), 3.90 (s, 2H).





**Figure S7.** Mass spectra of phosphatidyl saccharides characterized by LC-MS/MS.


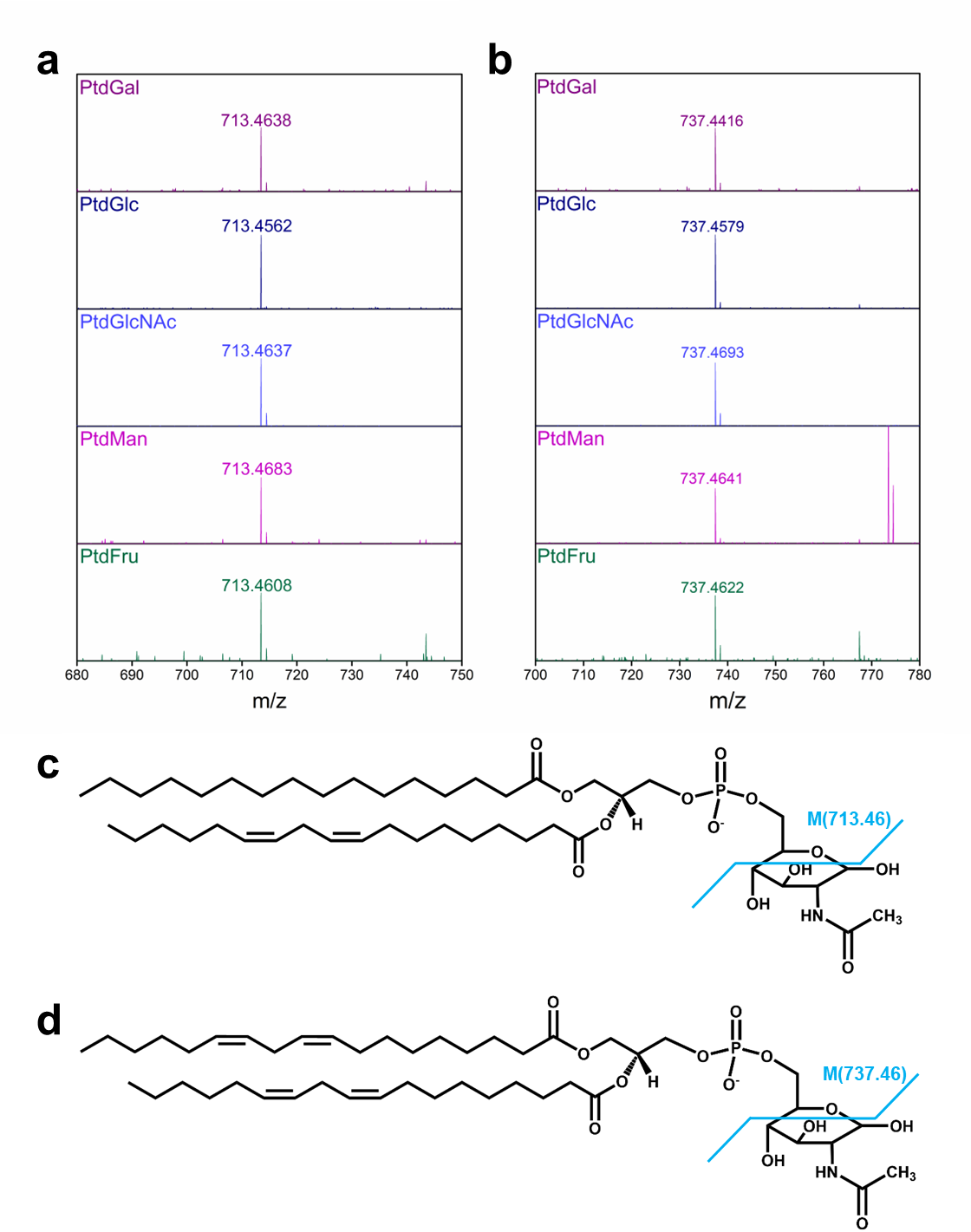


**Figure S8.** MS/MS of phosphatidyl saccharides. (a-b) MS/MS results for phosphatidyl saccharides composed of two different lipid chains ([18:2, 18:2], [16:0, 18:2]), respectively. (c-d) Molecular structure of the phospholipid part composed of two aliphatic chains that can be connected with the glycosyl 6-OH.





**Figure S9.** Infrared spectra of phosphatidyl saccharides characterized by ATR-FTIR.


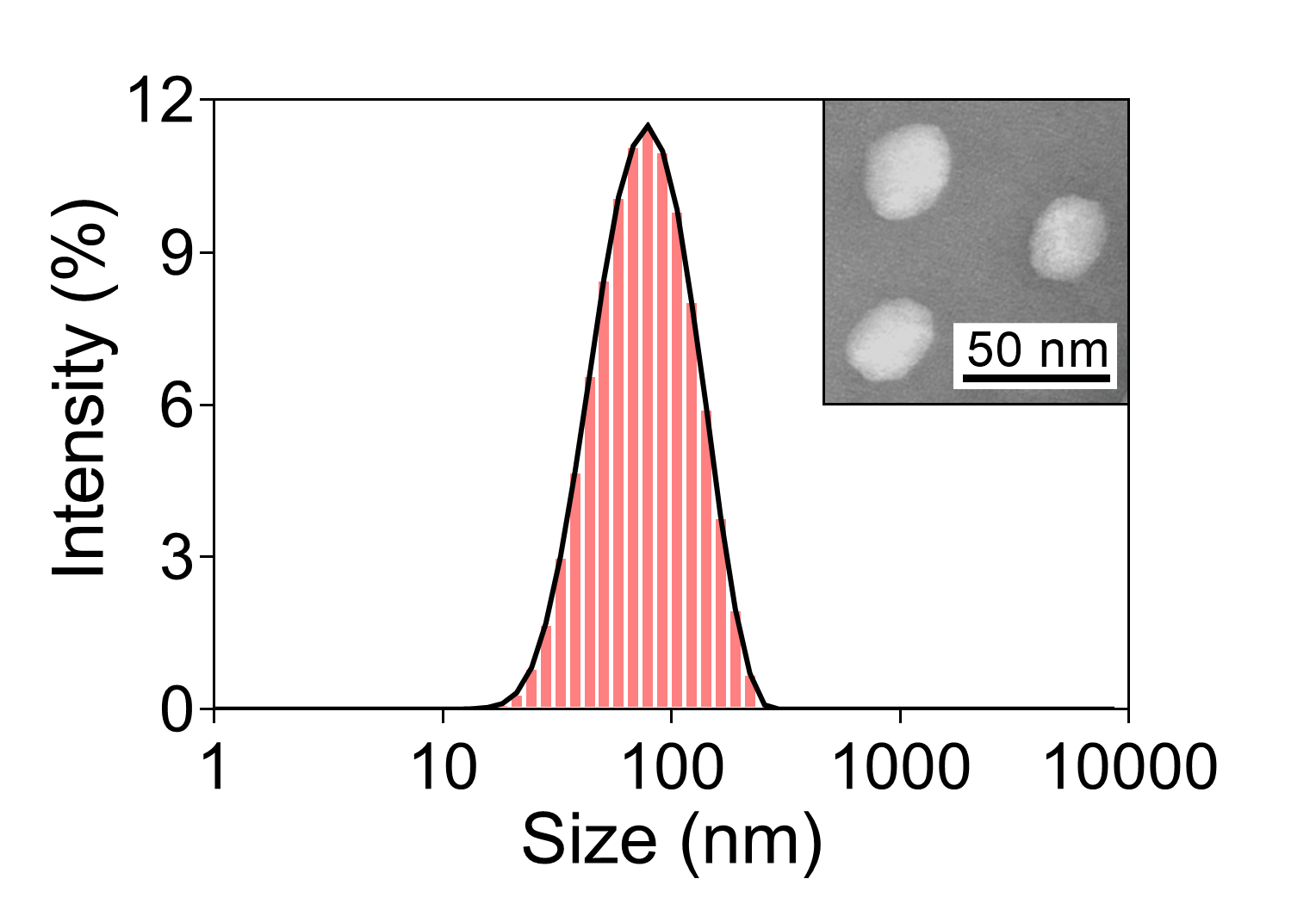


**Figure S10.** Hydrodynamic diameter distributions of PA-LNPs measured by DLS (insets: corresponding TEM micrographs).


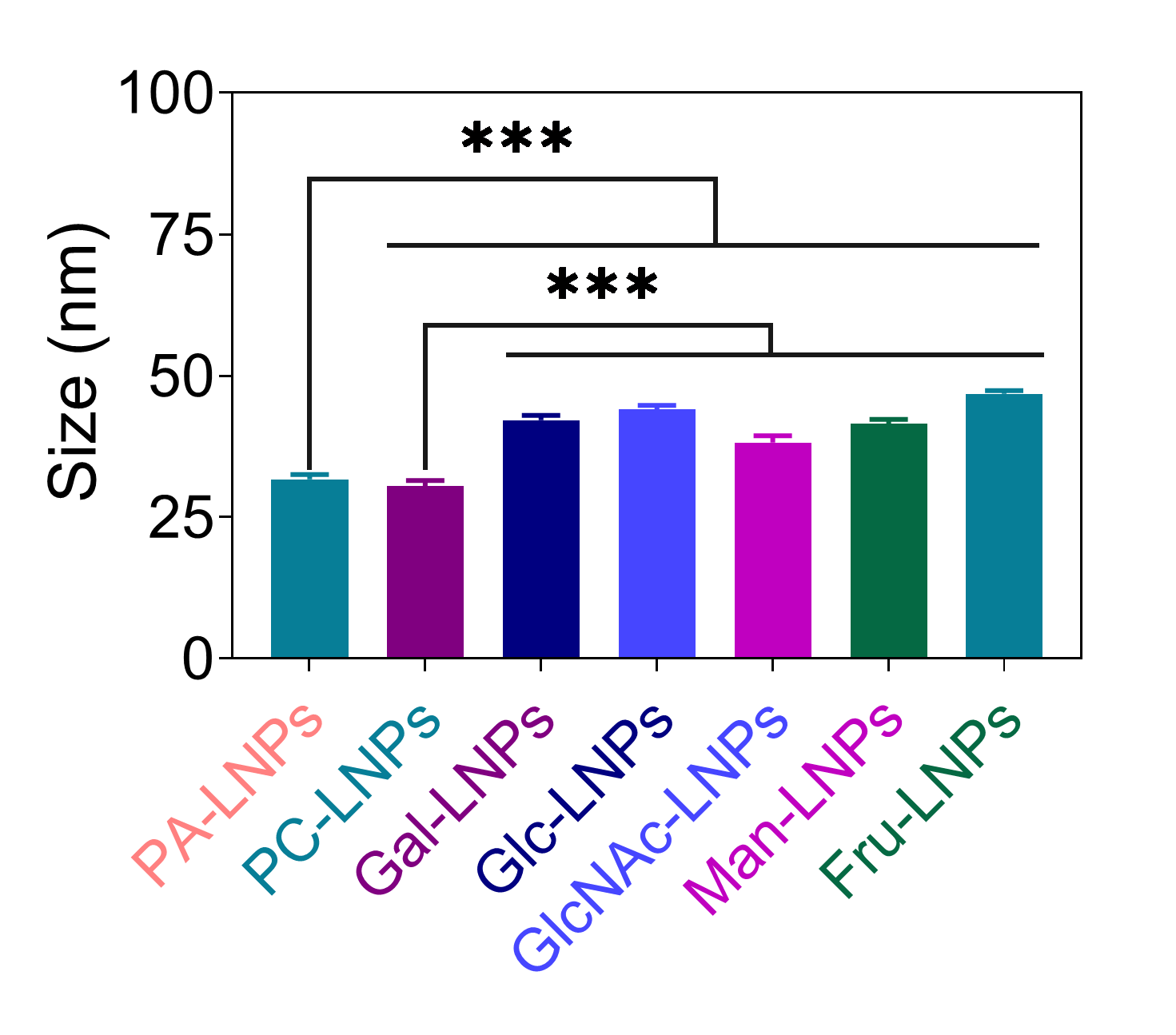


**Figure S11**. Average particle size of G-LNPs determined by transmission electron microscopy and analyzed by Nano Measurer software 1.2. Data are presented as mean ± SD (*n* = 50). Statistical significance was tested with one-way ANOVA. **p* < 0.05, ***p* < 0.01, ****p* < 0.001.


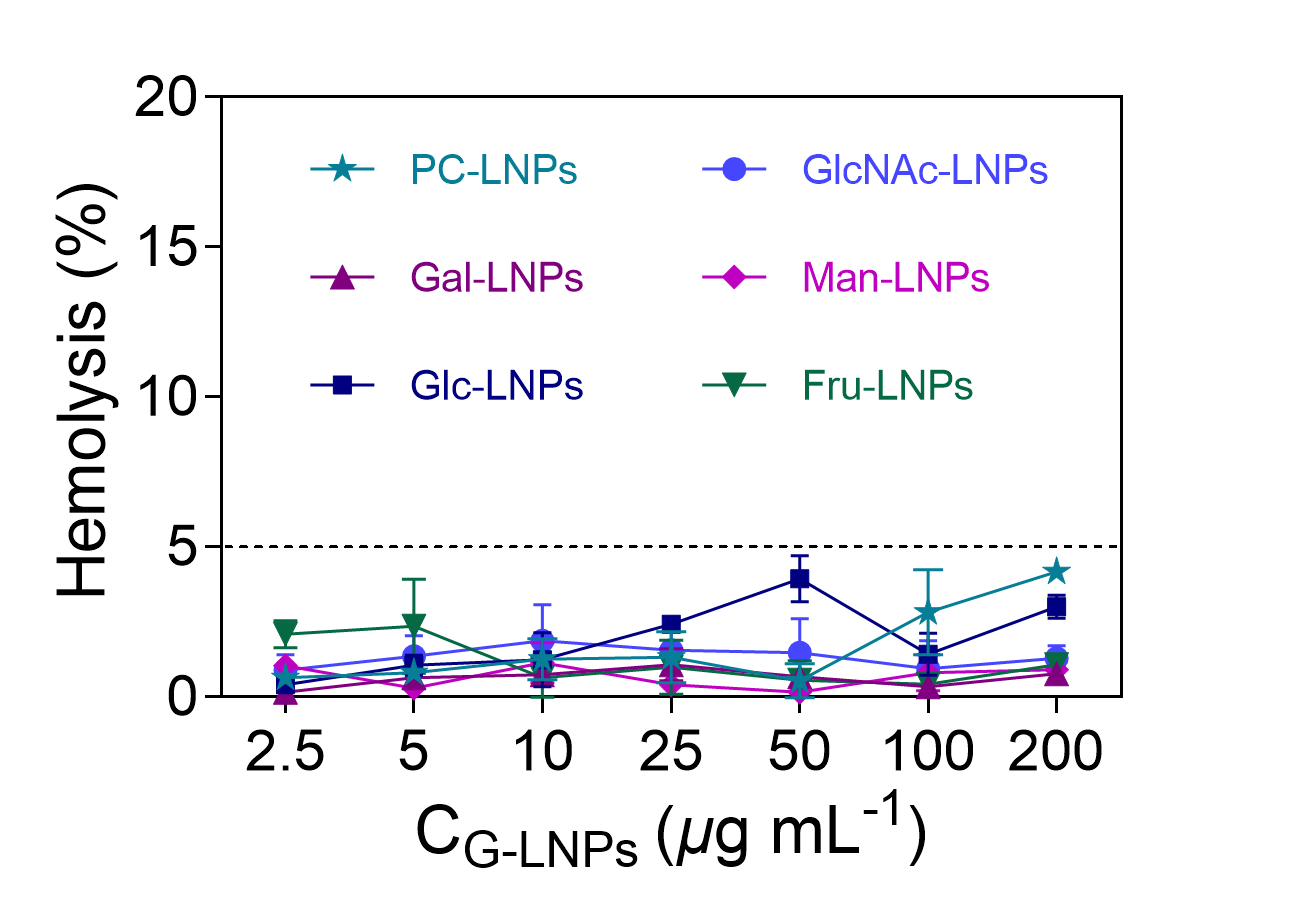


**Figure S12.** Hemolysis of G-LNPs at various concentrations. Data are presented as mean ± SD (*n* = 3).


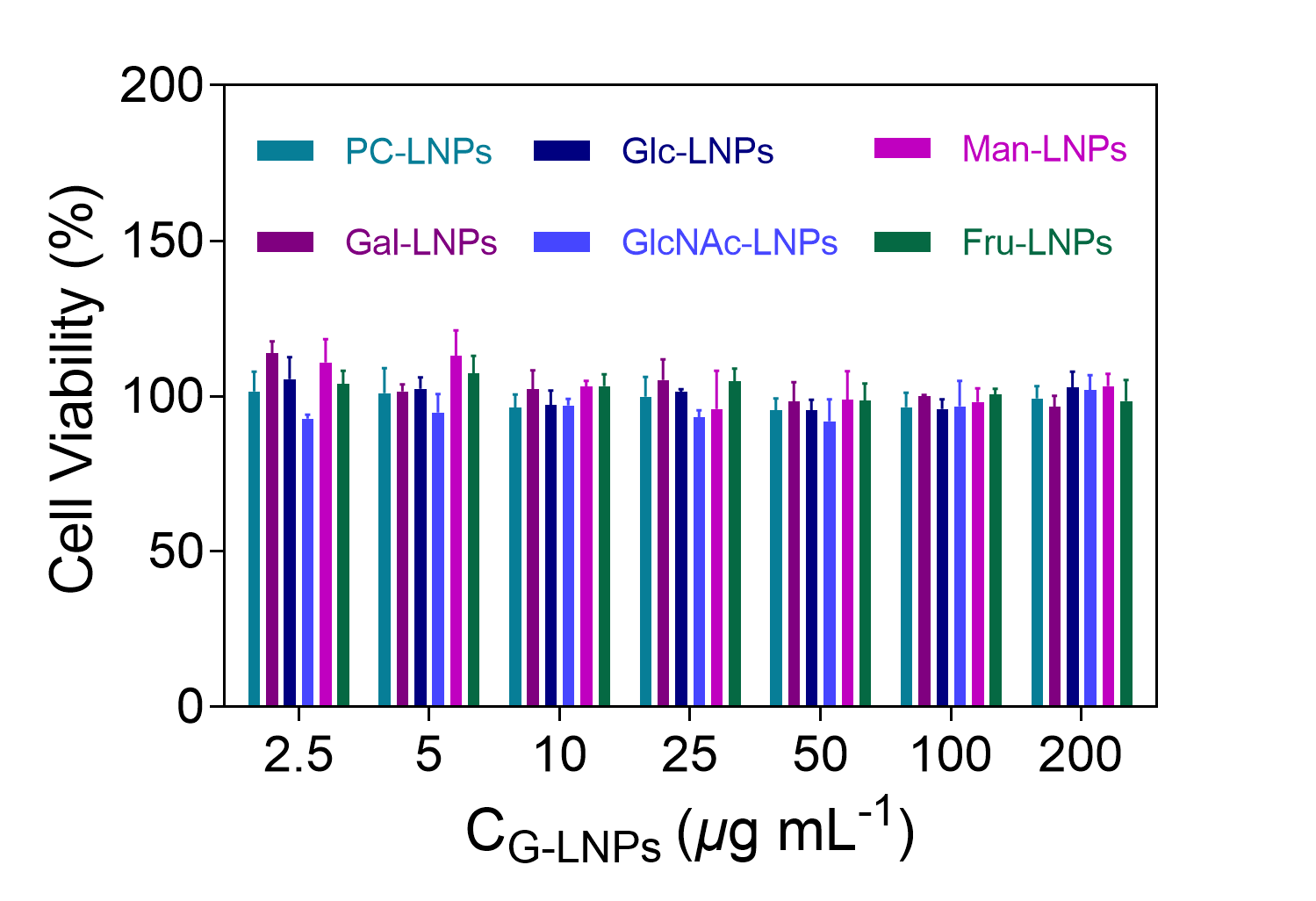


**Figure S13.** Cell viability of HUVECs cells treated with G-LNPs at various concentrations. Data are presented as mean ± SD (*n* = 3).


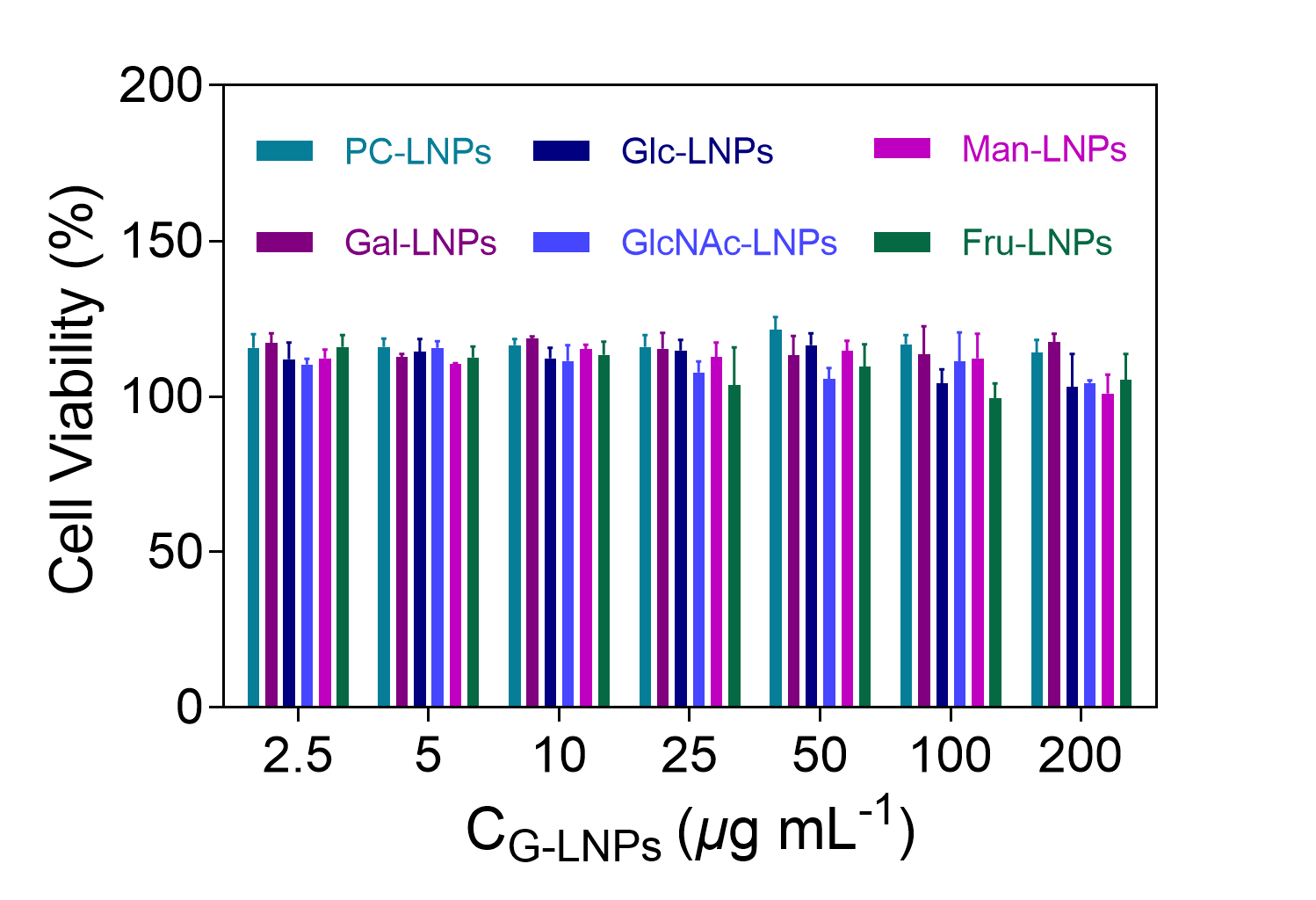


**Figure S14.** Cell viability of L929 cells treated with G-LNPs at various concentrations. Data are presented as mean ± SD (*n* = 3).


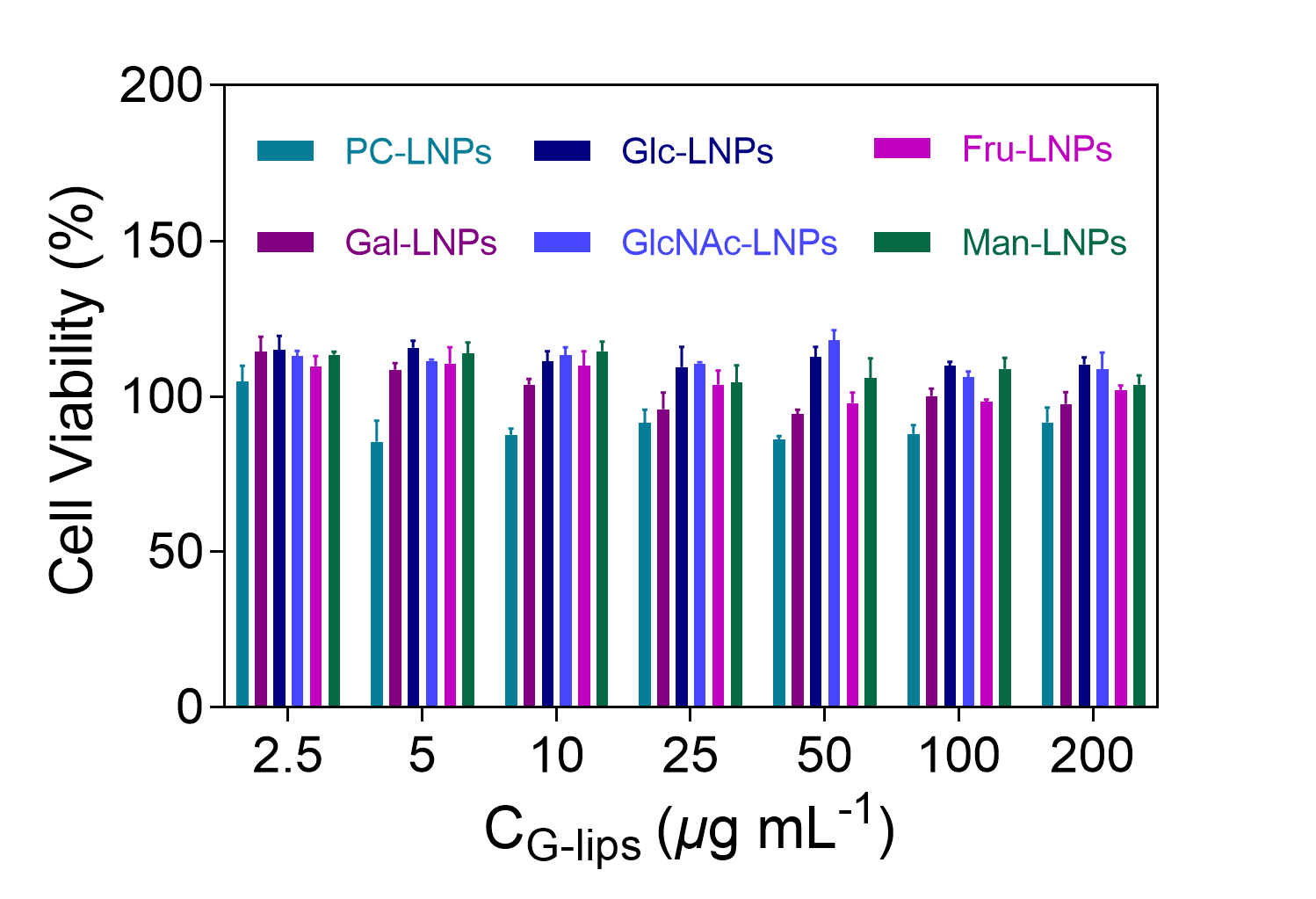


**Figure S15.** Cell viability of RAW264.7 cells treated with G-LNPs at various concentrations. Data are presented as mean ± SD (*n* = 3).


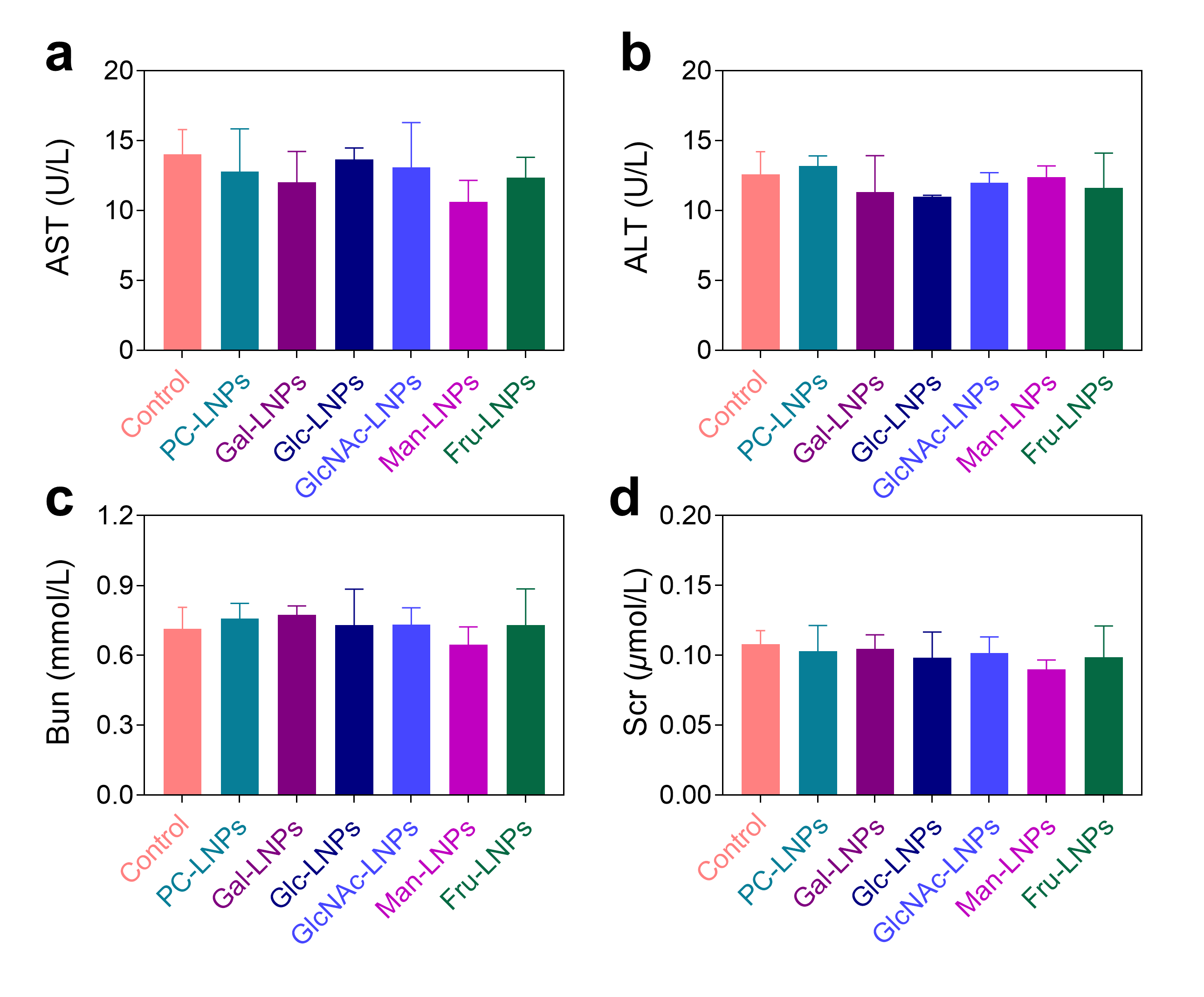


**Figure S16.** Blood biochemistry test of mice after treatment with G-LNPs at 24 h post-injection. AST (a), ALT (b), Bun (c), Scr (d). Data are presented as mean ± SD (*n* = 3).


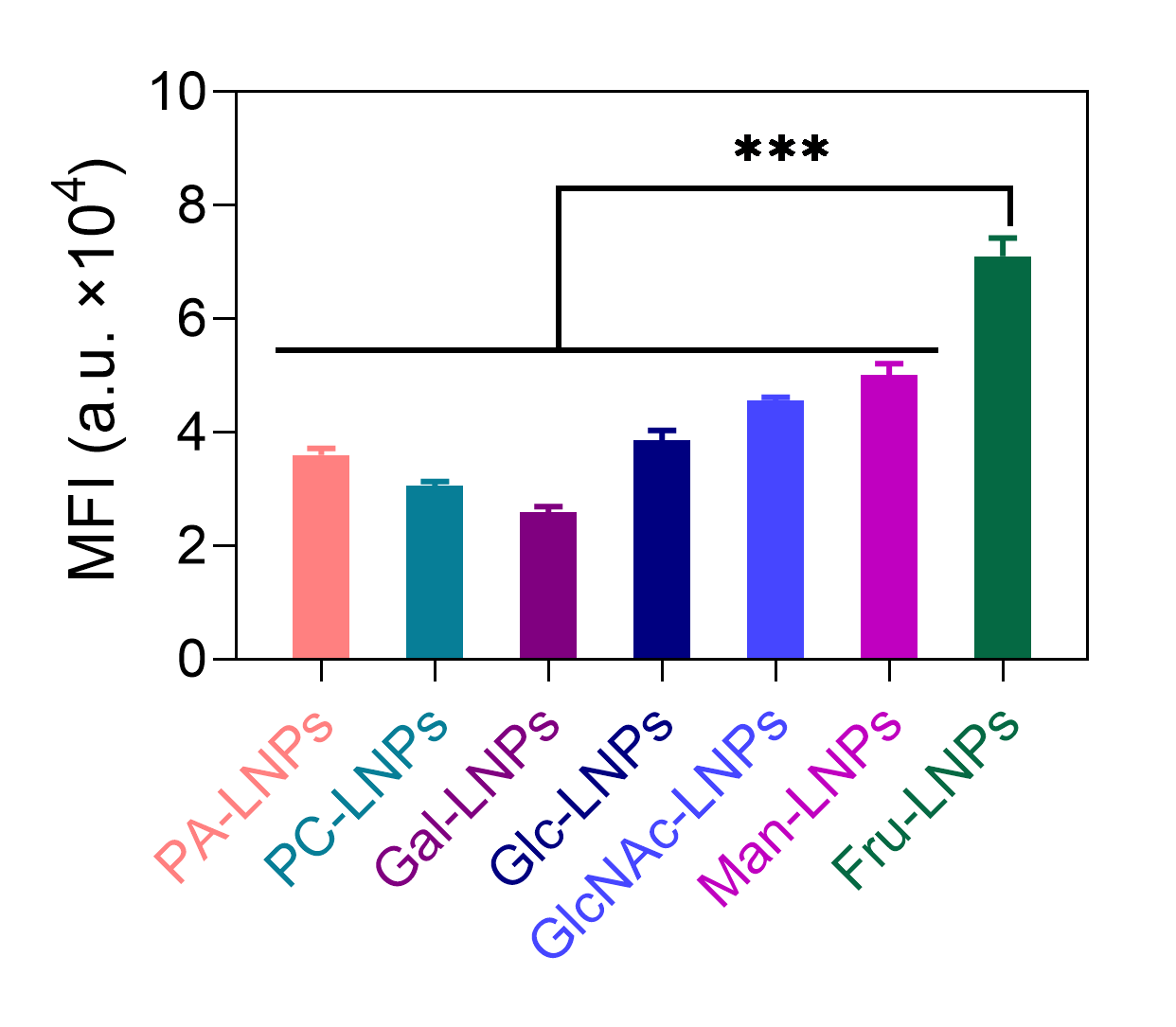


**Figure S17.** Flow cytometry analysis of G-LNPs uptake in 4T1 cells. Data are presented as mean ± SD (*n* = 3).


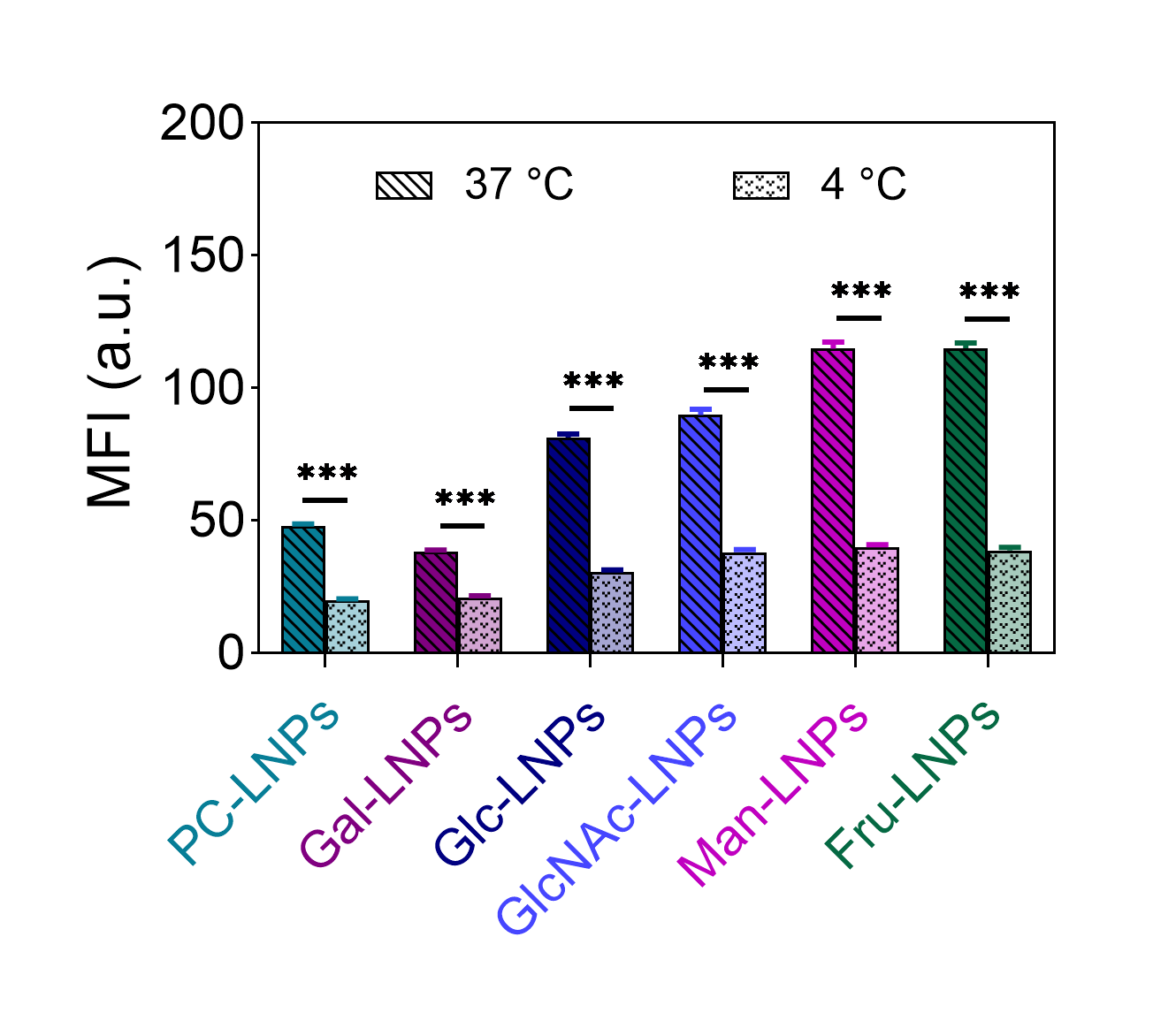


**Figure S18.** Uptake of G-LNPs by 4T1 cells at different temperatures. Data are presented as mean ± SD (*n* = 3).


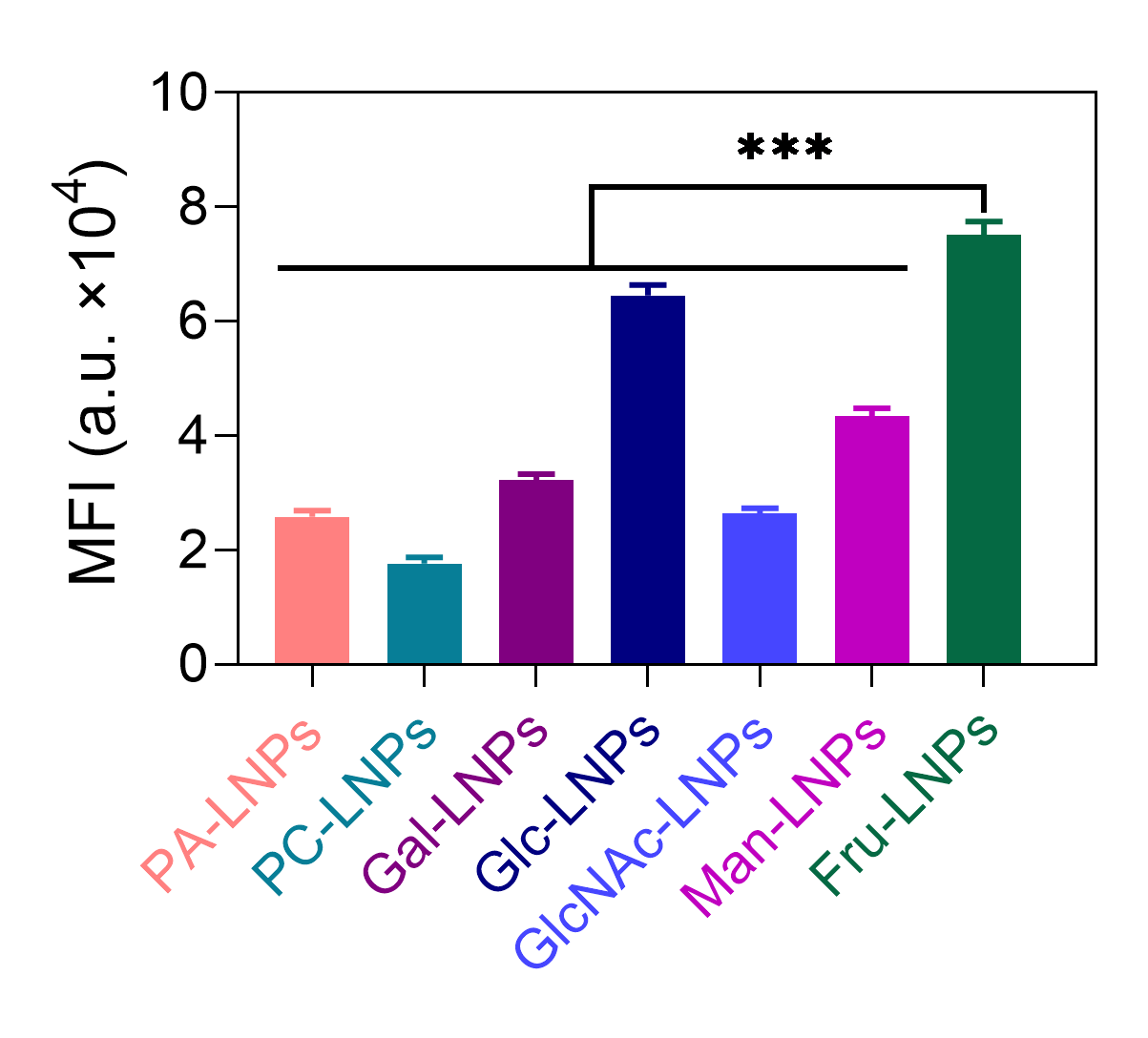


**Figure S19.** Flow cytometry analysis of G-LNPs uptake in MCF-7 cells. Data are presented as mean ± SD (*n* = 3).


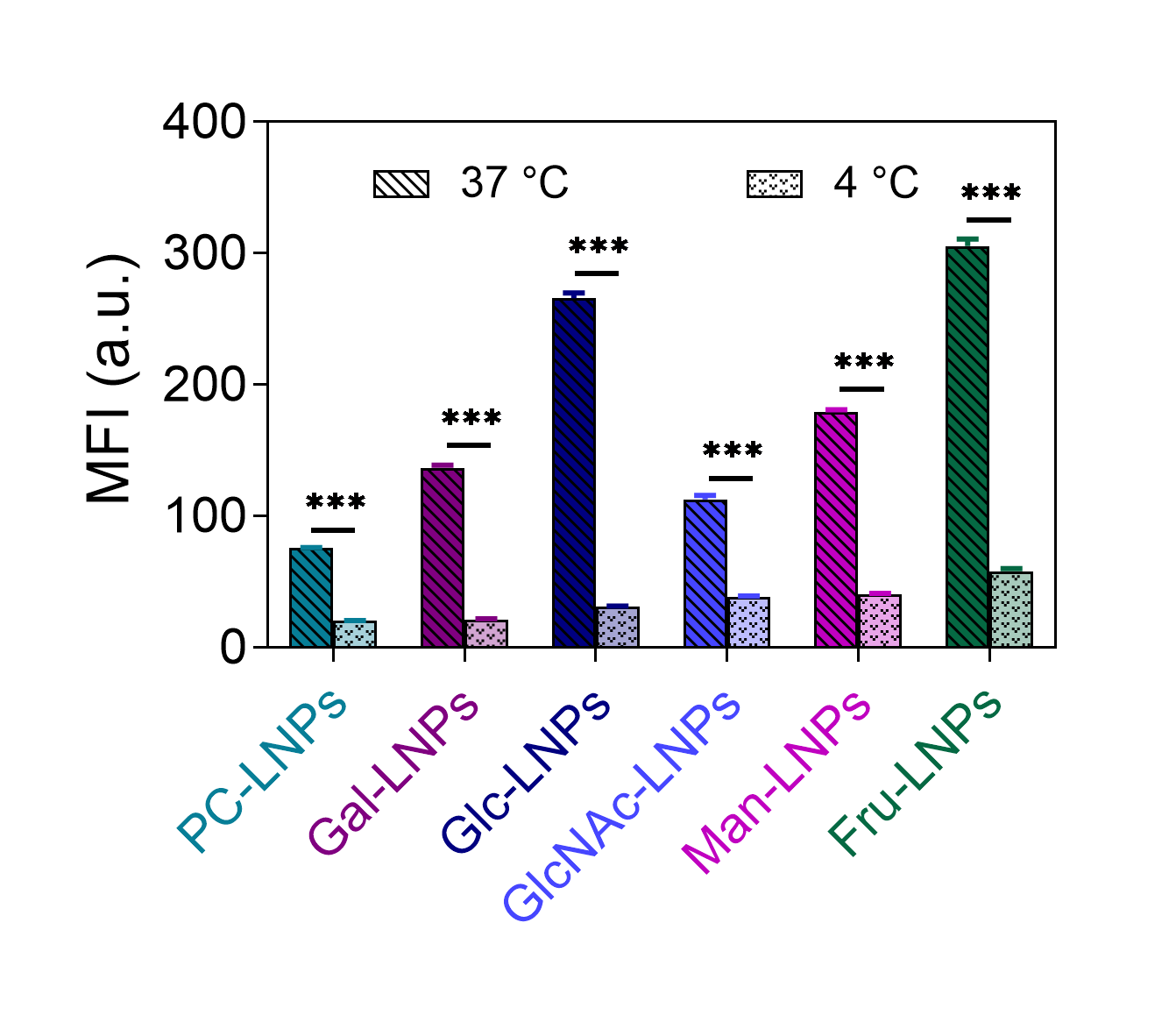


**Figure S20.** Uptake of G-LNPs by MCF-7 cells at different temperatures. Data are presented as mean ± SD (*n* = 3).


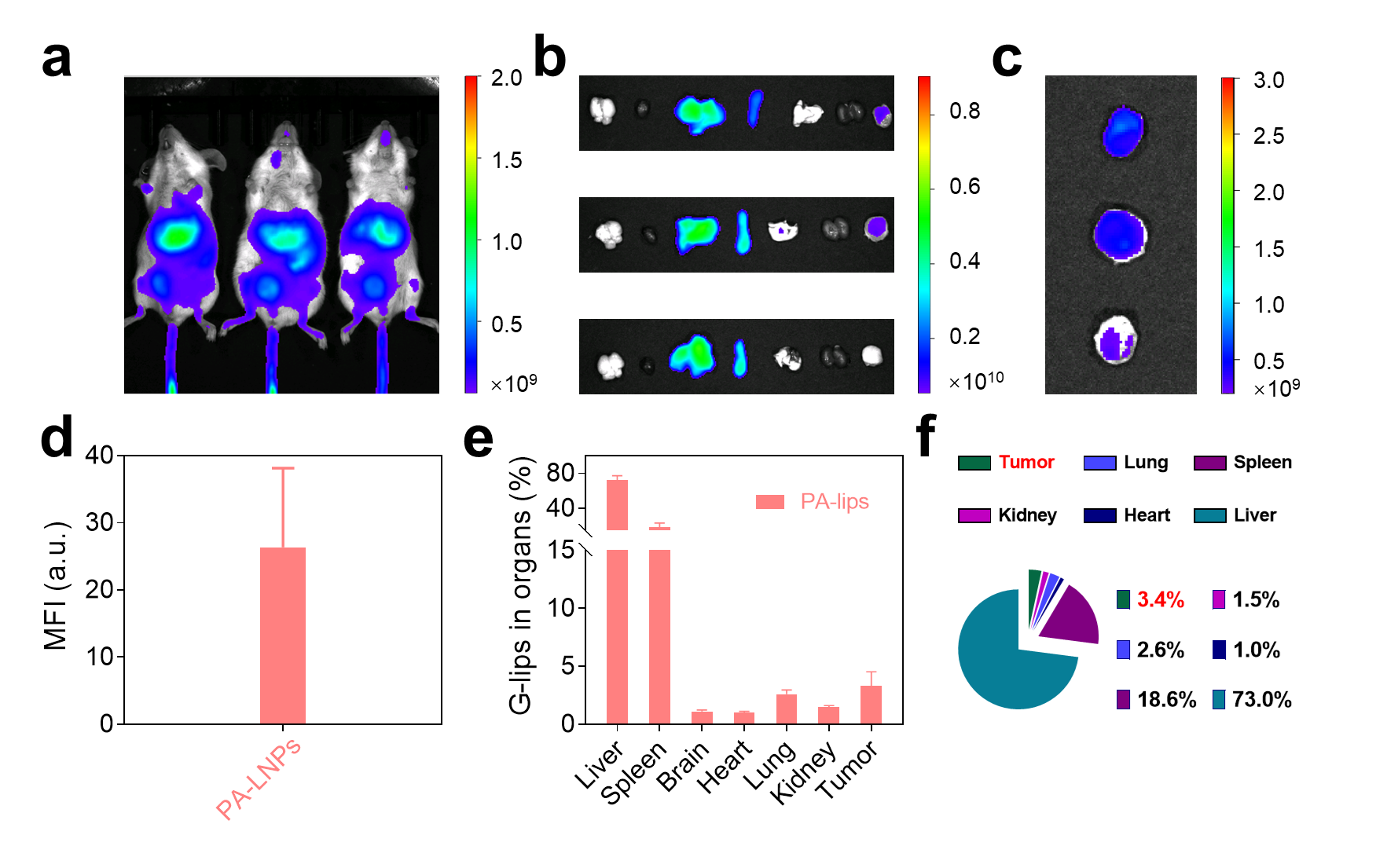


**Figure S21.** Tumor targeting behavior of PA-LNPs. (a) *In vivo* fluorescence images of mice and (b) *In vitro* fluorescence images of major organs at 24 h post intravenous injection of PA-LNPs. (c) *In vitro* fluorescence images of tumors. (d) Quantitative analysis of the tumor fluorescence intensity of PA-LNPs from *in vitro* imaging. (e) Quantitative analysis of the fluorescence intensity of PA-LNPs in all major organs from *in vitro* imaging. (f) Pie charts depicting the fluorescence intensity distribution percentages of PA-LNPs in major organs. Data are presented as mean ± SD (*n* = 3).


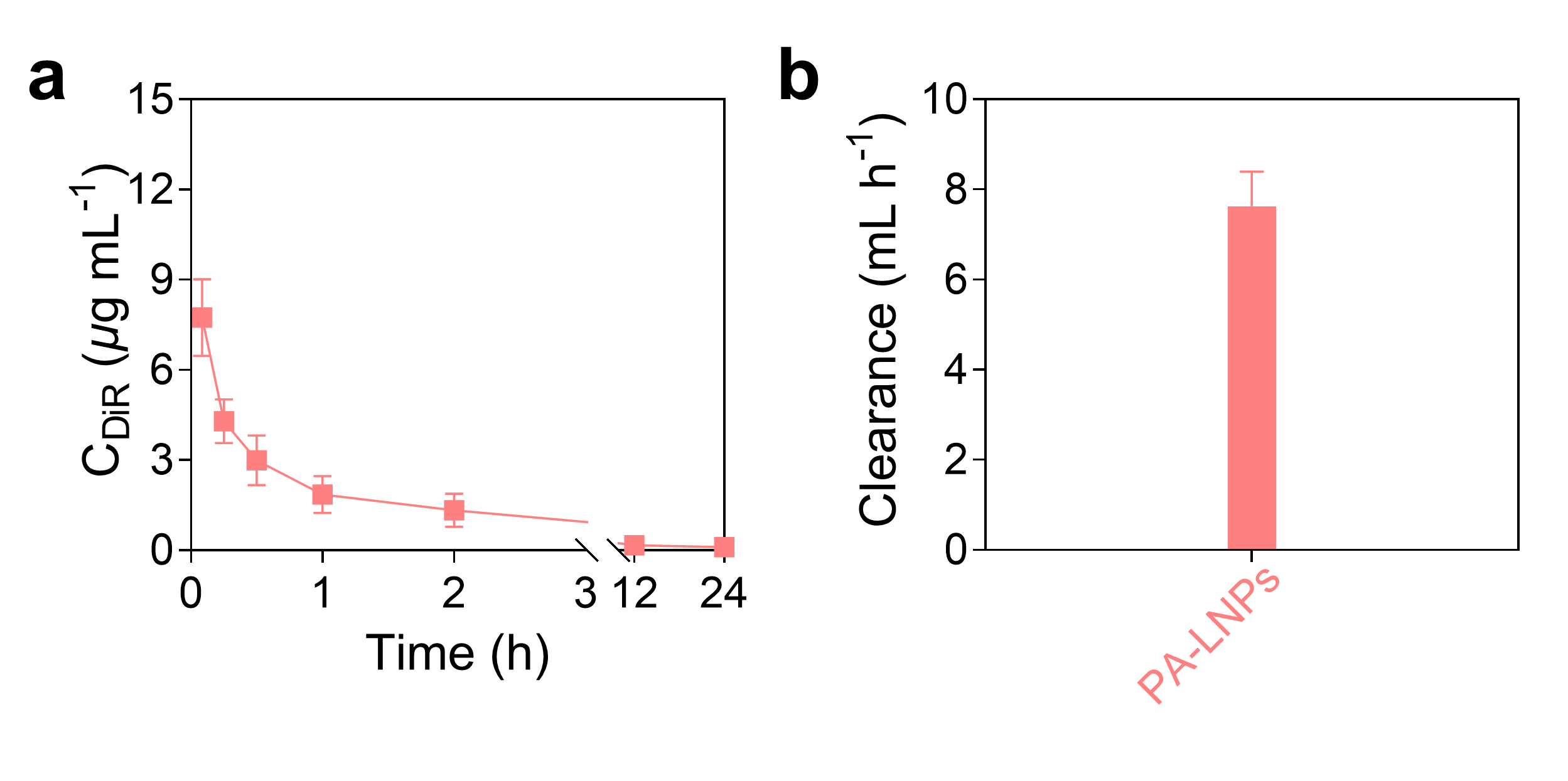


**Figure S22.** Blood concentration-time curves (a) and total clearance (b) of PA-LNPs. Data are presented as mean ± SD (*n* = 6).


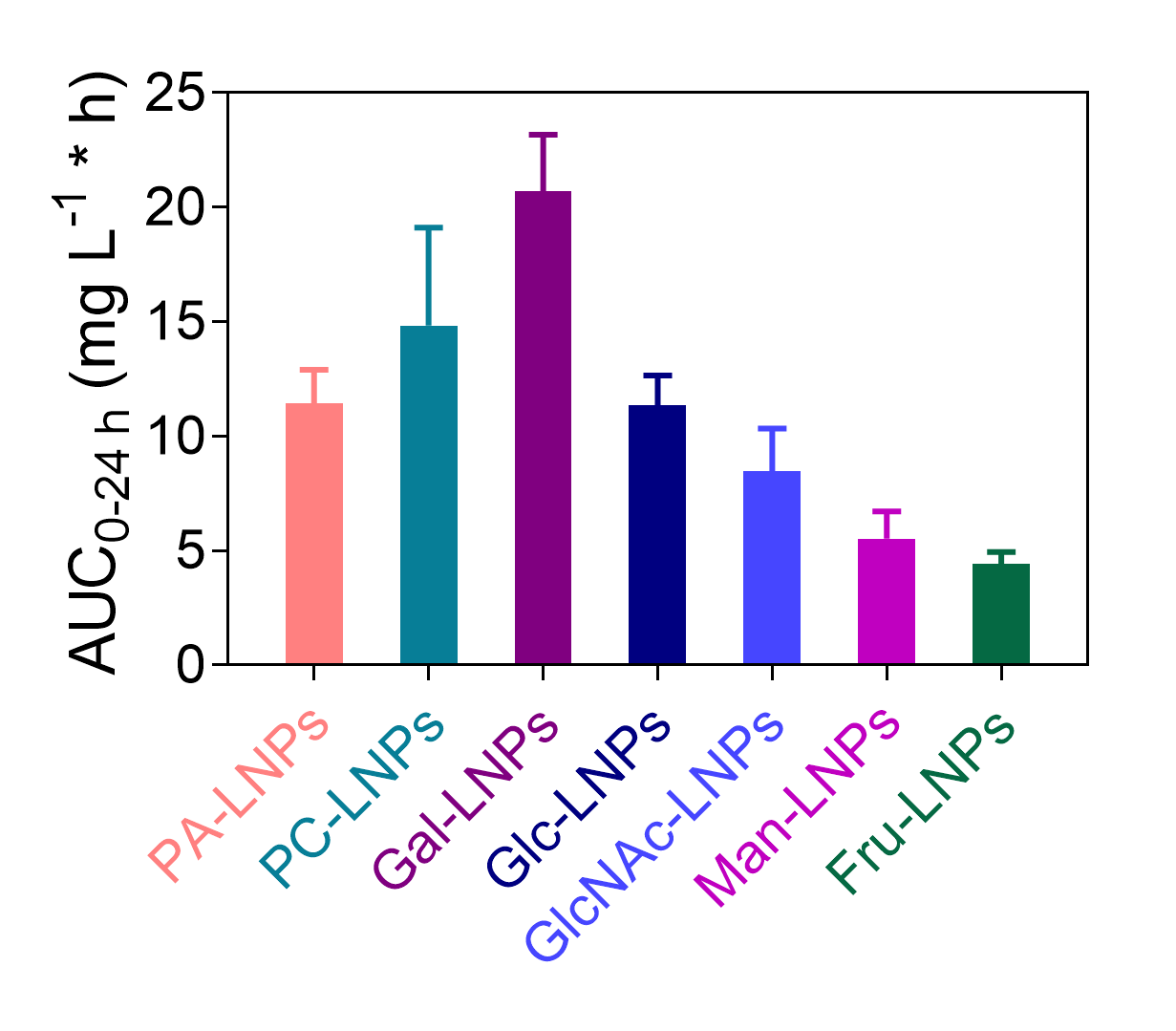


**Figure S23.** Area under the blood concentration-time curve (AUC) for G-LNPs. Data are presented as mean ± SD (*n* = 6).


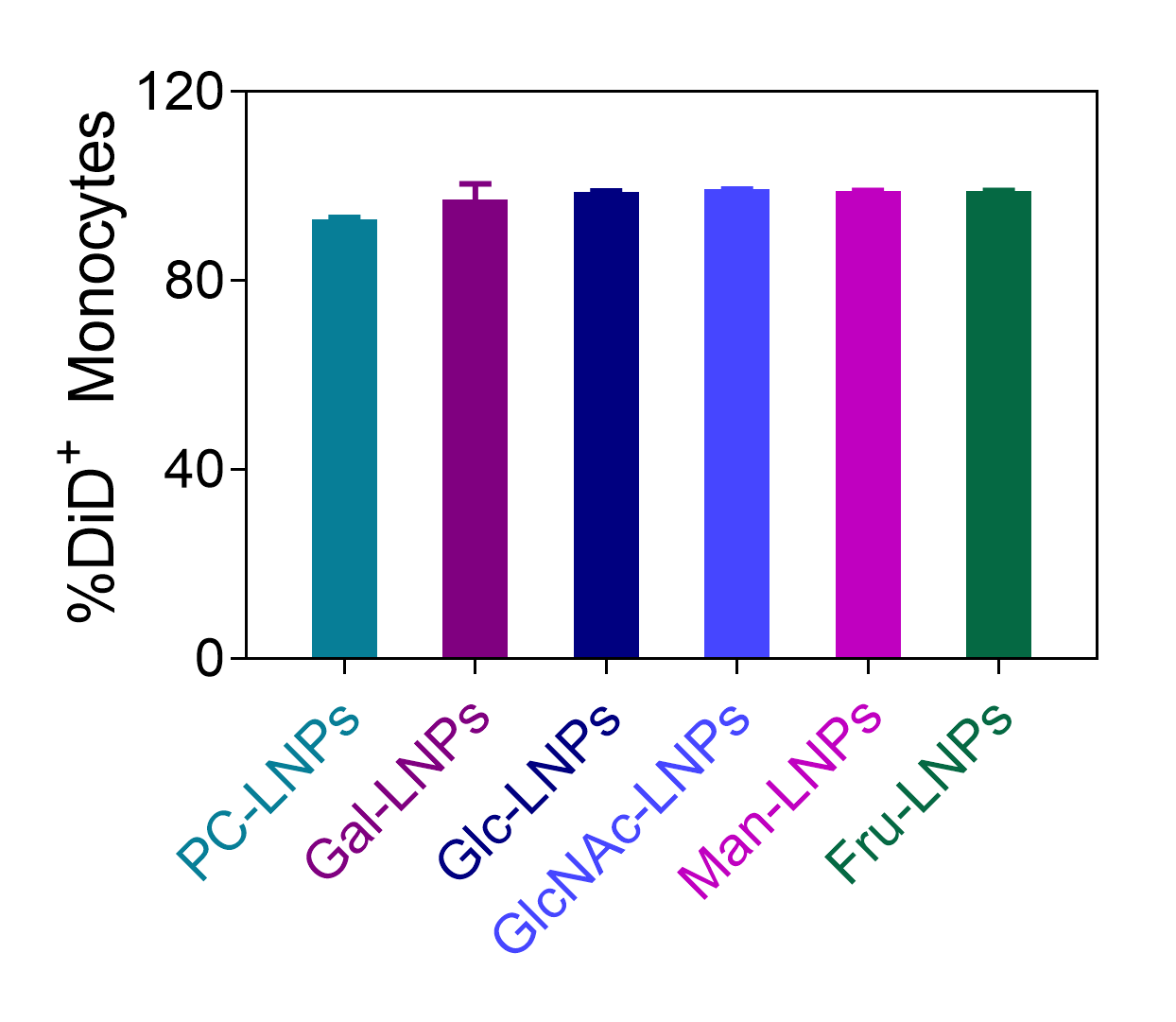


**Figure S24.** Percentage of monocytes that was DiD-positive at 0.5 h post-injection. Data are presented as mean ± SD (*n* = 3).


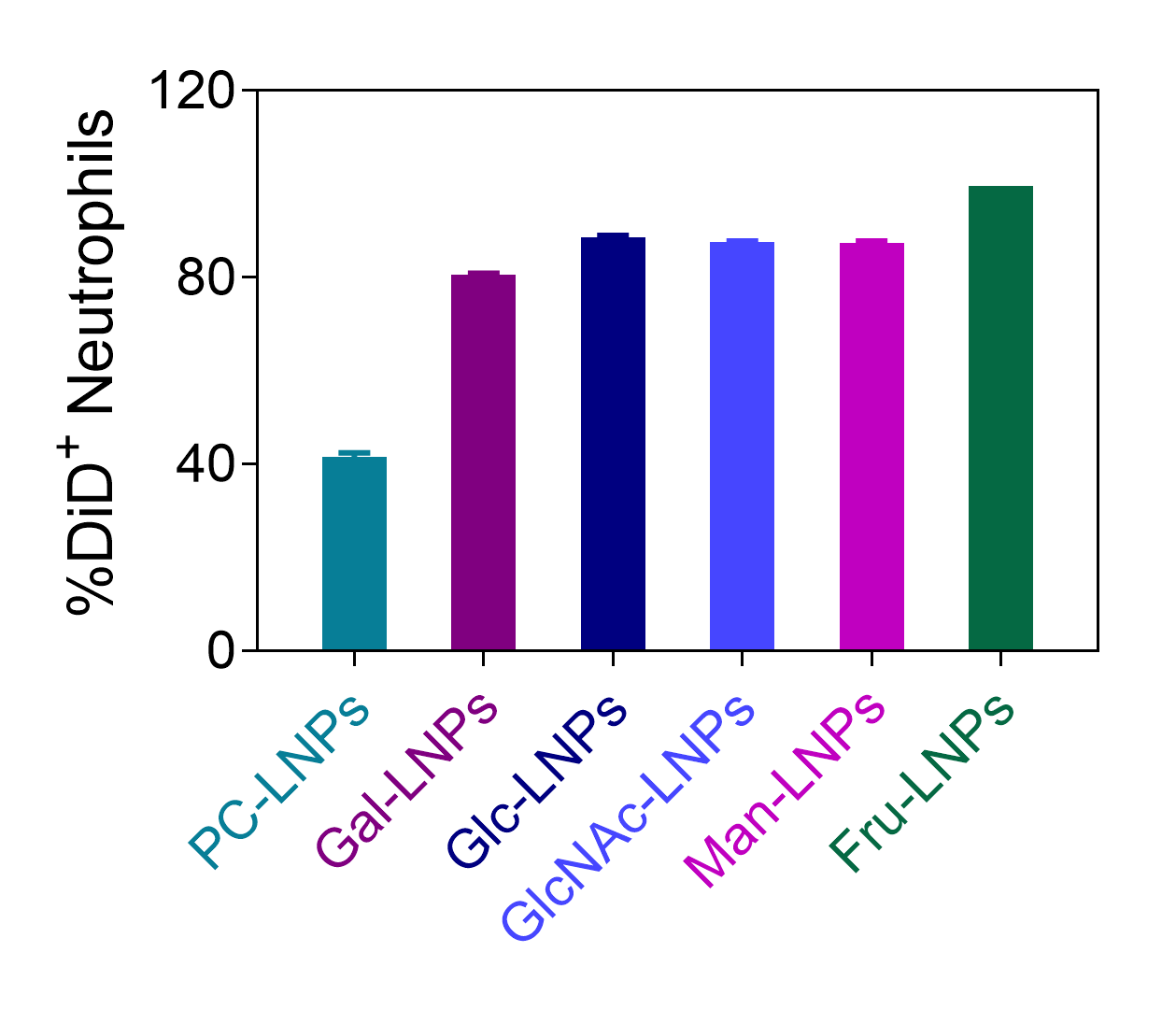


**Figure S25.** Amounts of neutrophils that were DiD-positive at 0.5 h post-injection. Data are presented as mean ± SD (*n* = 3).


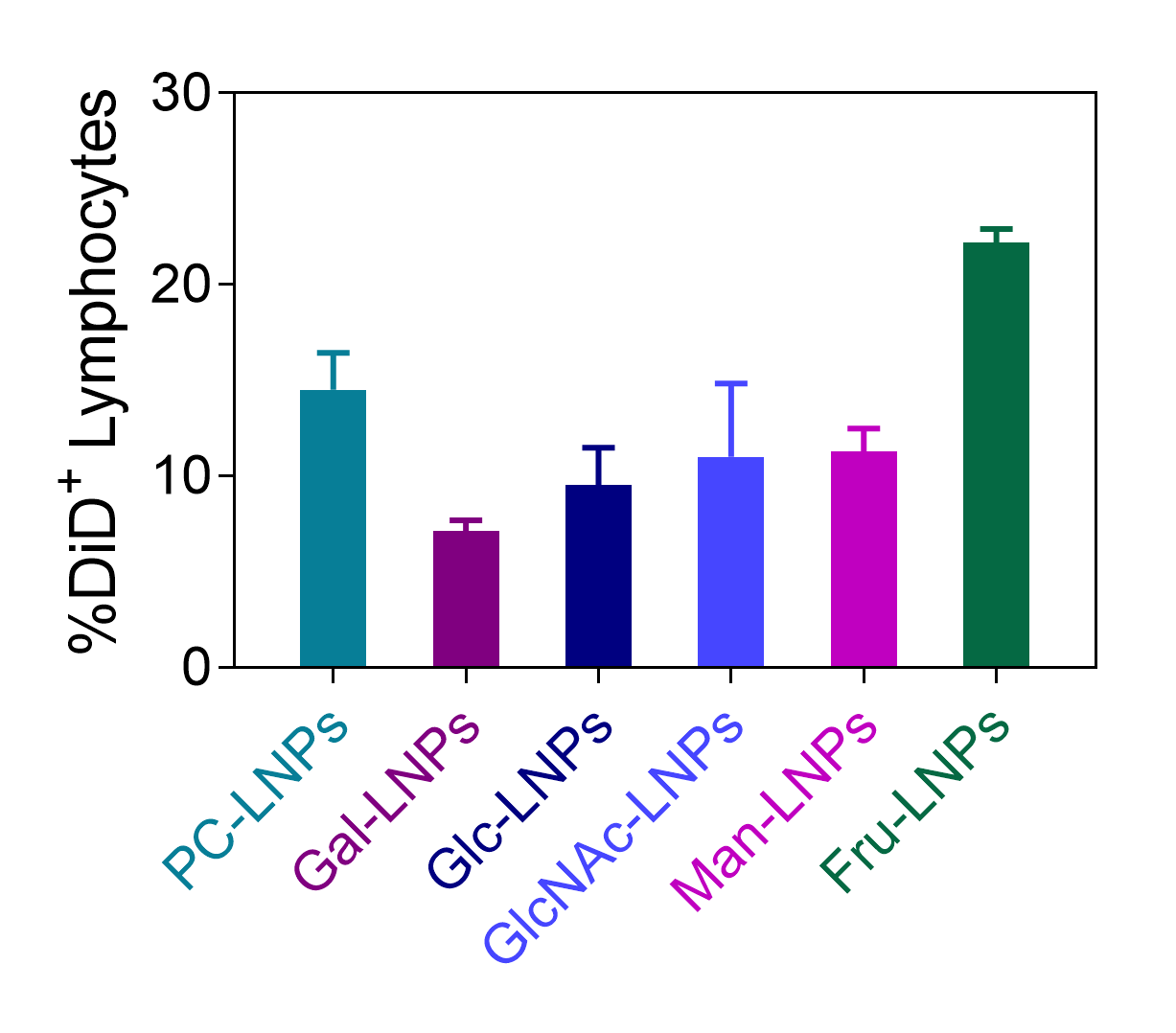


**Figure S26.** Amounts of lymphocytes that were DiD-positive at 0.5 h post-injection. Data are presented as mean ± SD (*n* = 3).


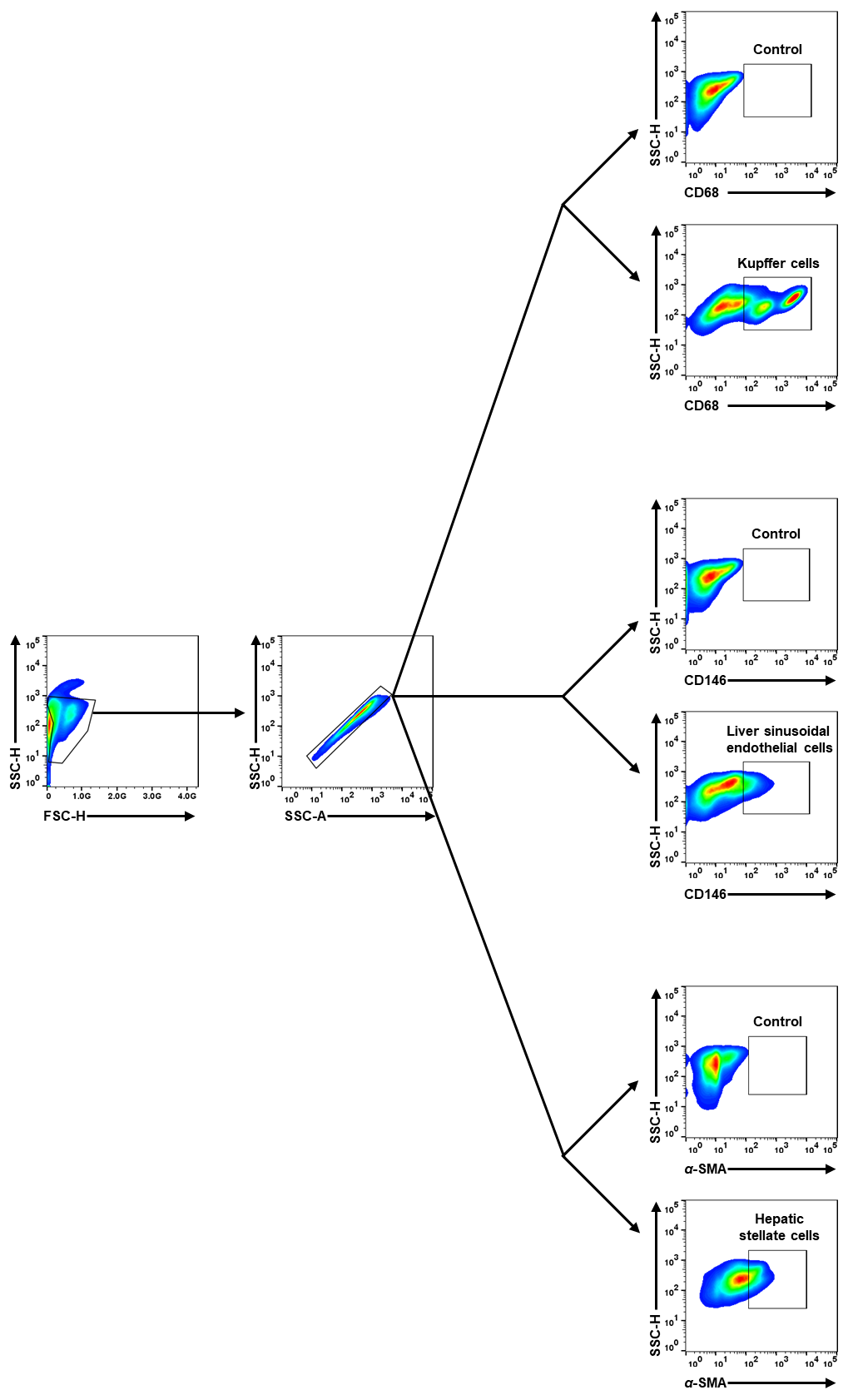


**Figure S27.** Flow cytometry gating strategy utilized to identify hepatic nonparenchymal cell fractions in *in vivo* experiments.


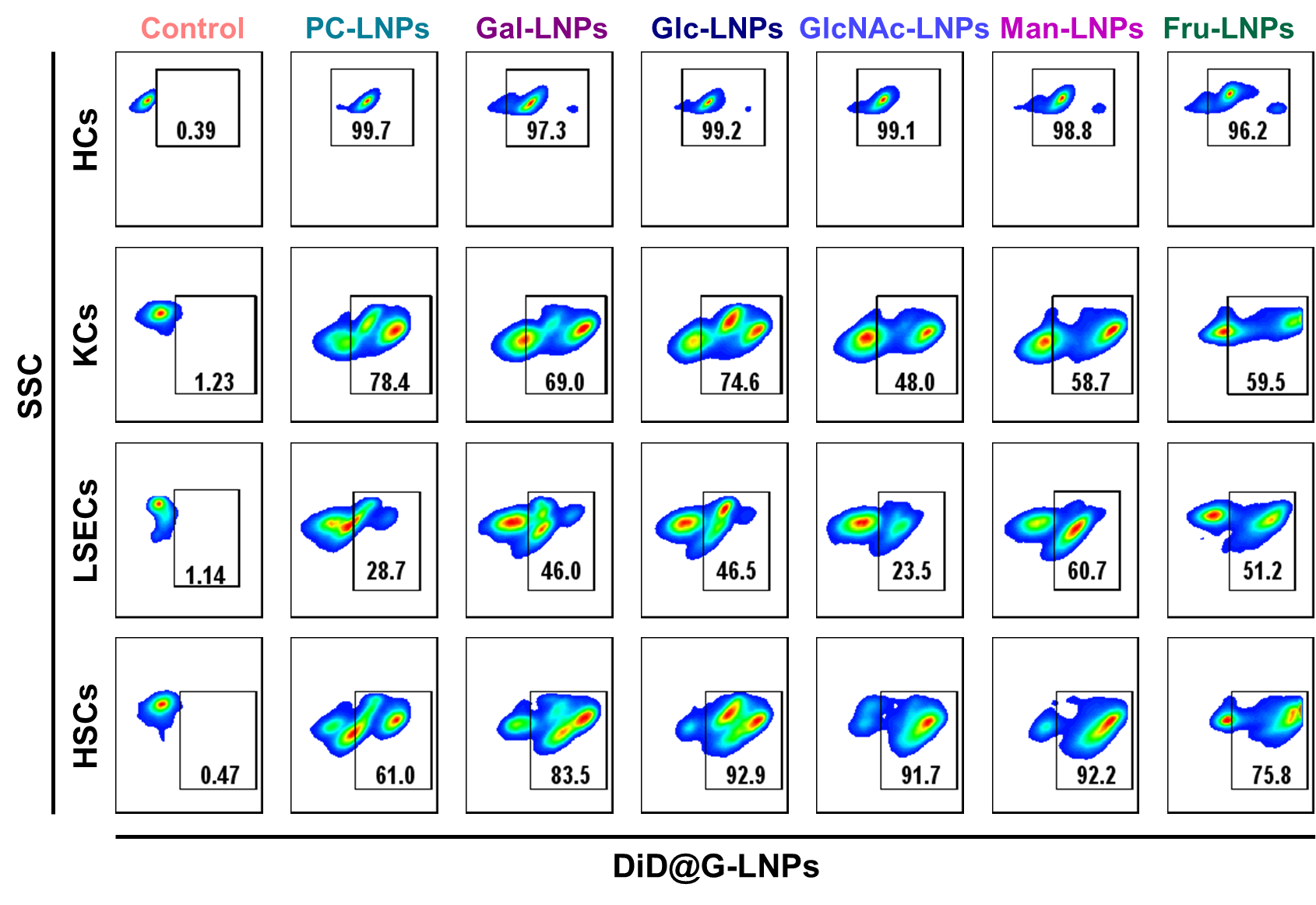


**Figure S28.** Representative flow plots showing DiD@G-LNPs uptake by hepatic cells at 2 h post-injection.


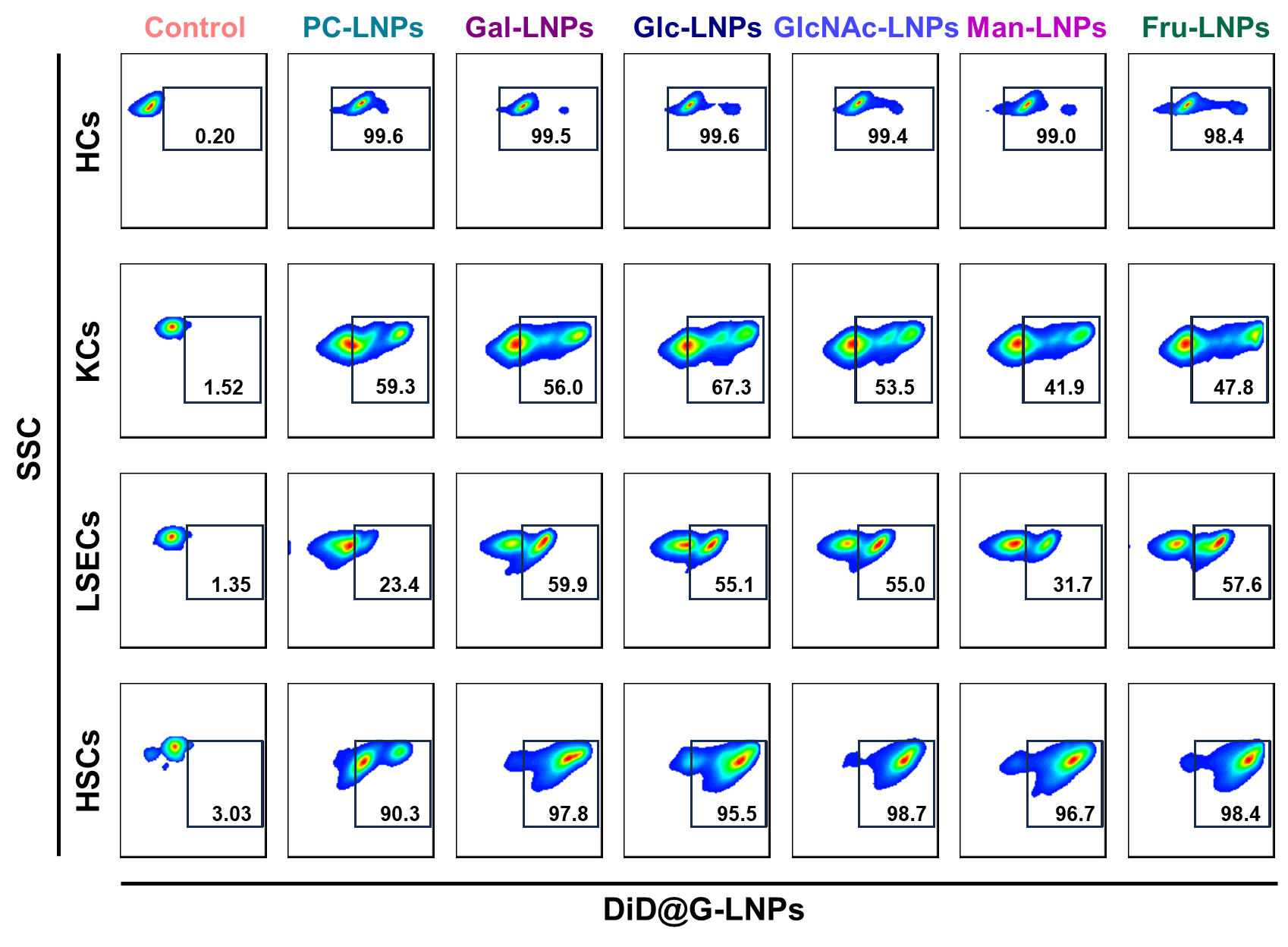


**Figure S29.** Representative flow plots showing DiD@G-LNPs uptake by hepatic cells at 12 h post-injection.


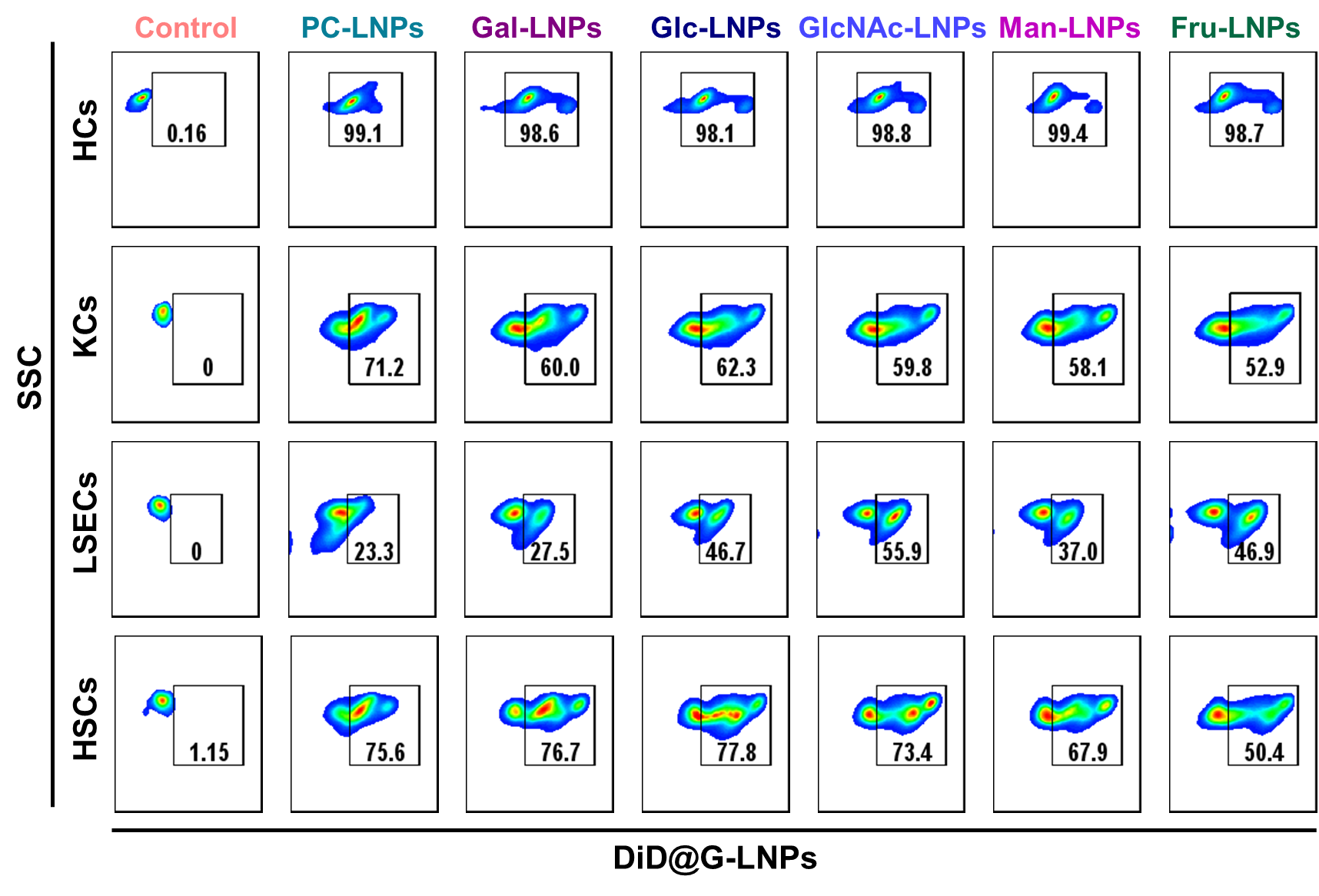


**Figure S30.** Representative flow plots showing DiD@G-LNPs uptake by hepatic cells at 24 h post-injection.


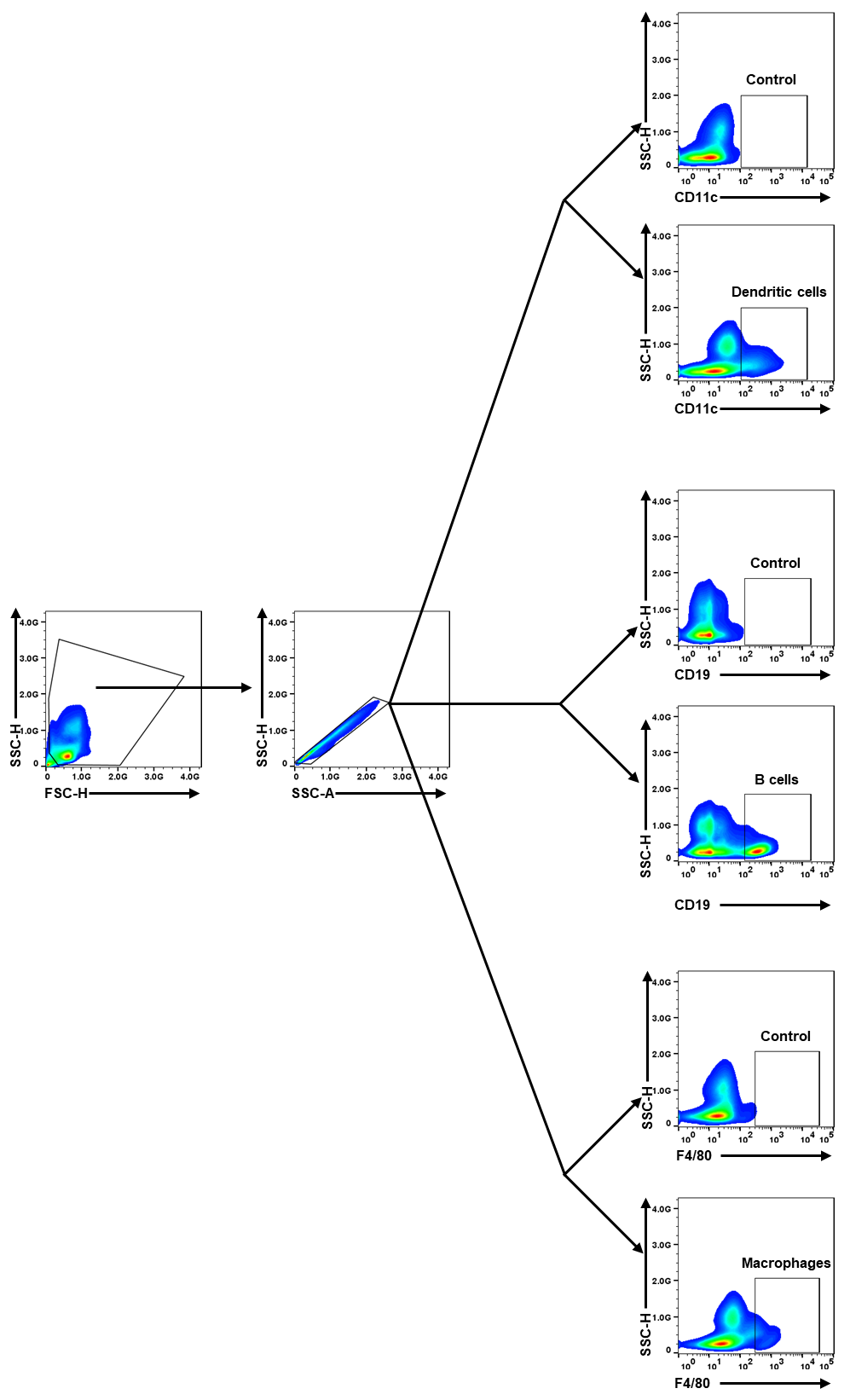


**Figure S31.** Flow cytometry gating strategy utilized to identify splenic antigen-presenting cell fractions in *in vivo* experiments.


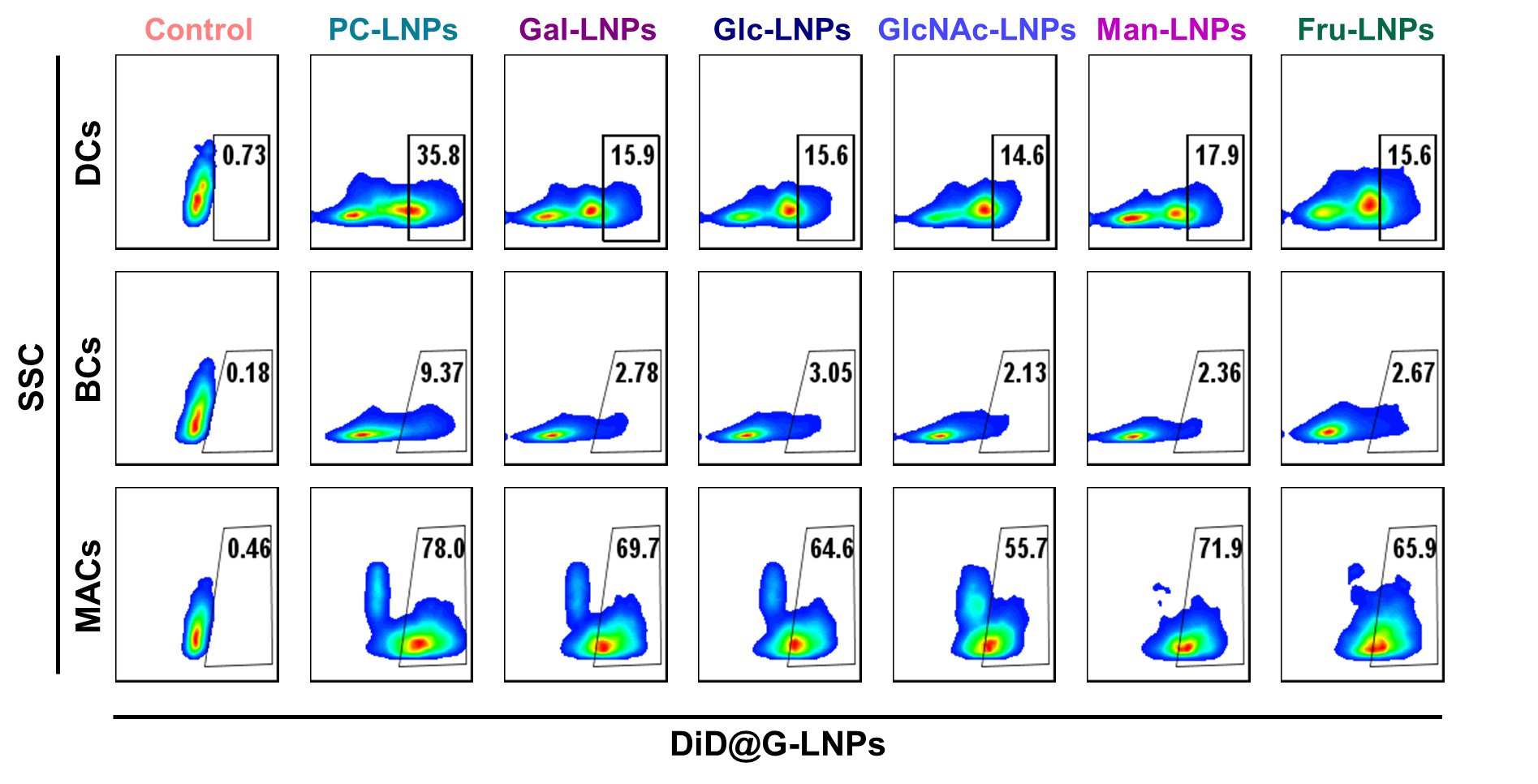


**Figure S32.** Representative flow plots showing DiI@G-LNPs uptake by splenic antigen-presenting cells at 2 h post-injection.


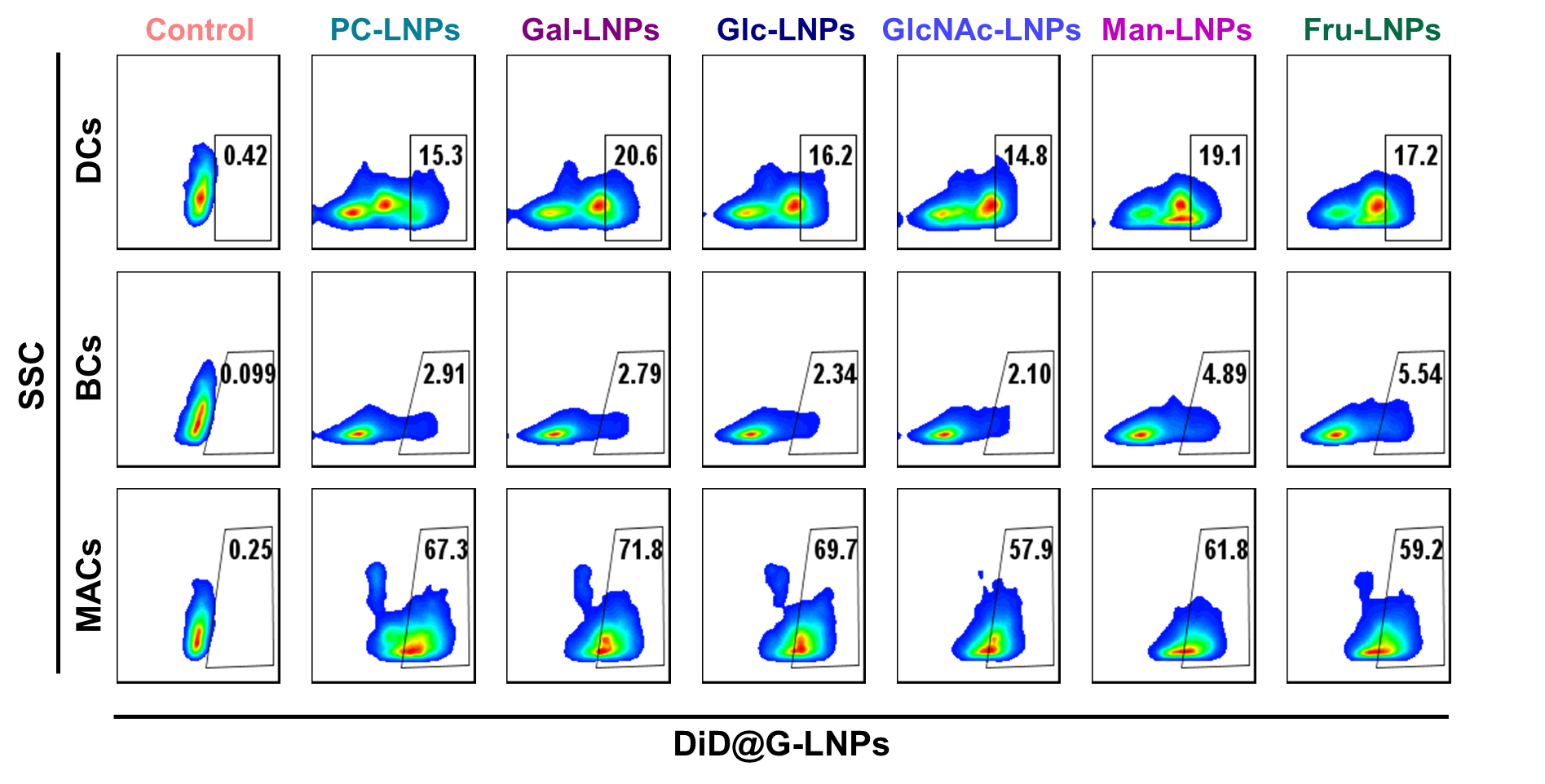


**Figure S33.** Representative flow plots showing DiI@G-LNPs uptake by splenic antigen-presenting cells at 12 h post-injection.


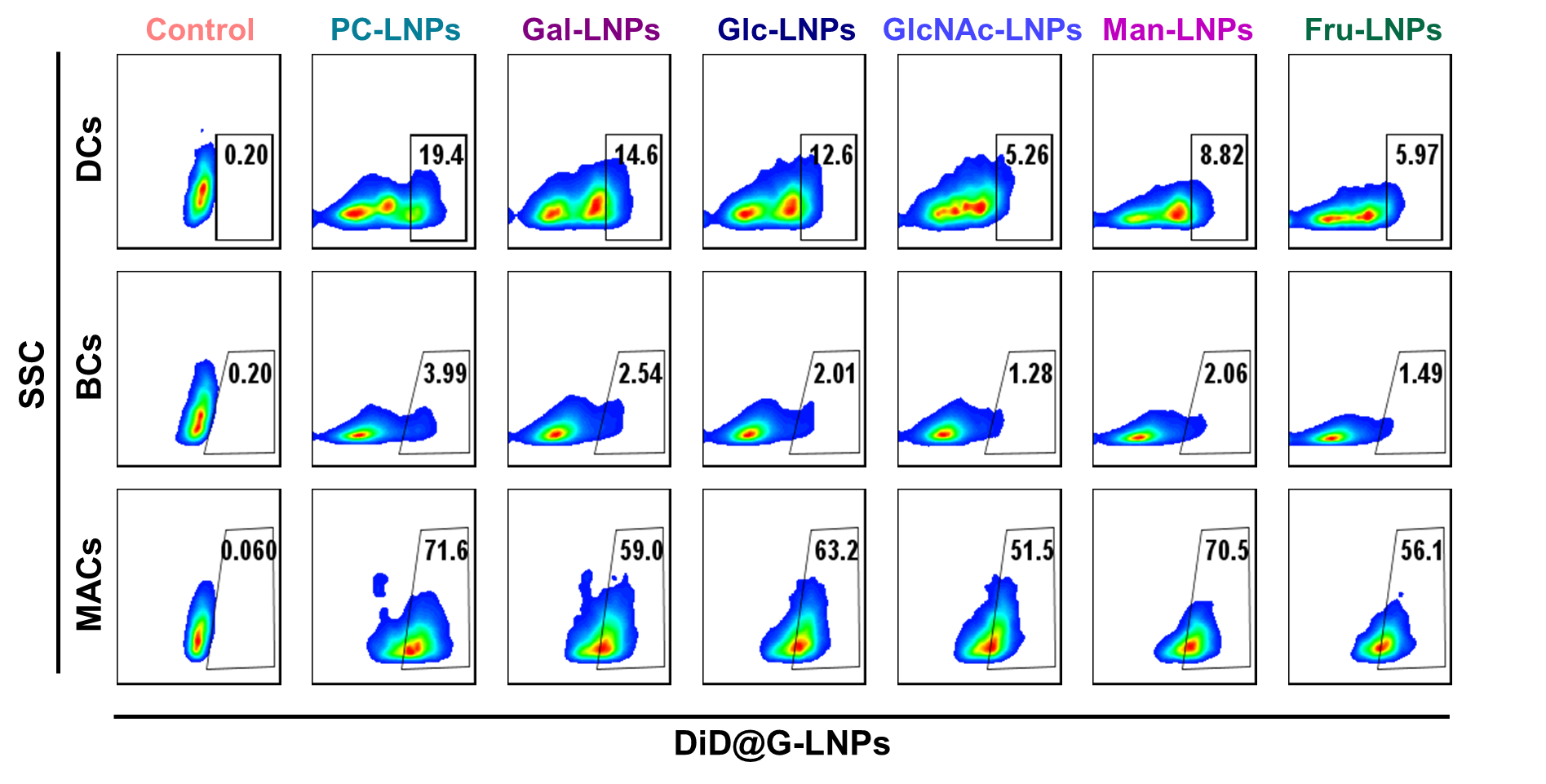


**Figure S34.** Representative flow plots showing DiI@G-LNPs uptake by splenic antigen-presenting cells at 24 h post-injection.


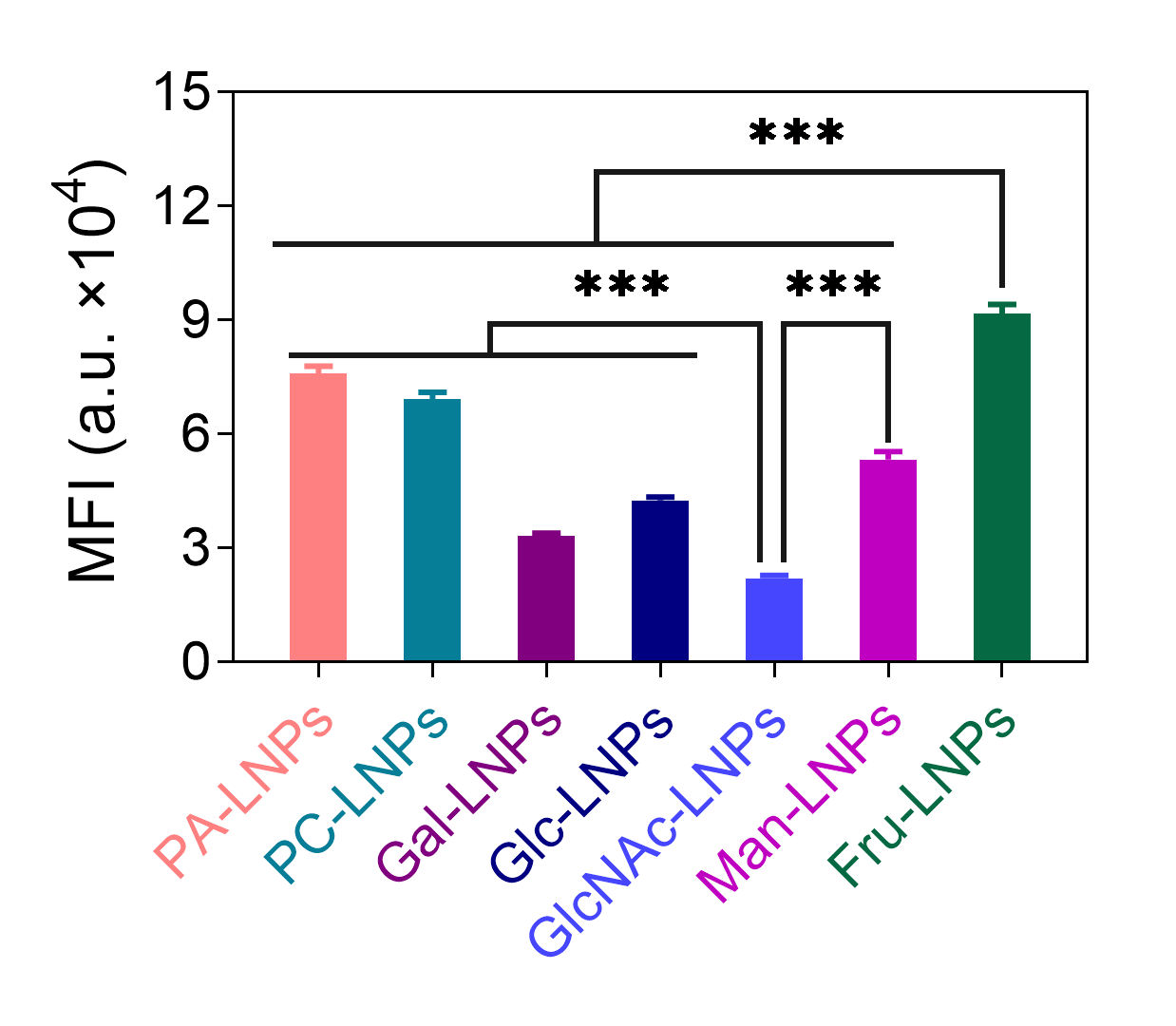


**Figure S35.** Uptake of breast cancer mouse serum (BCMS)-incubated G-LNPs by mouse-derived macrophage RAW264.7 cells. Data are presented as mean ± SD (*n* = 3).

**
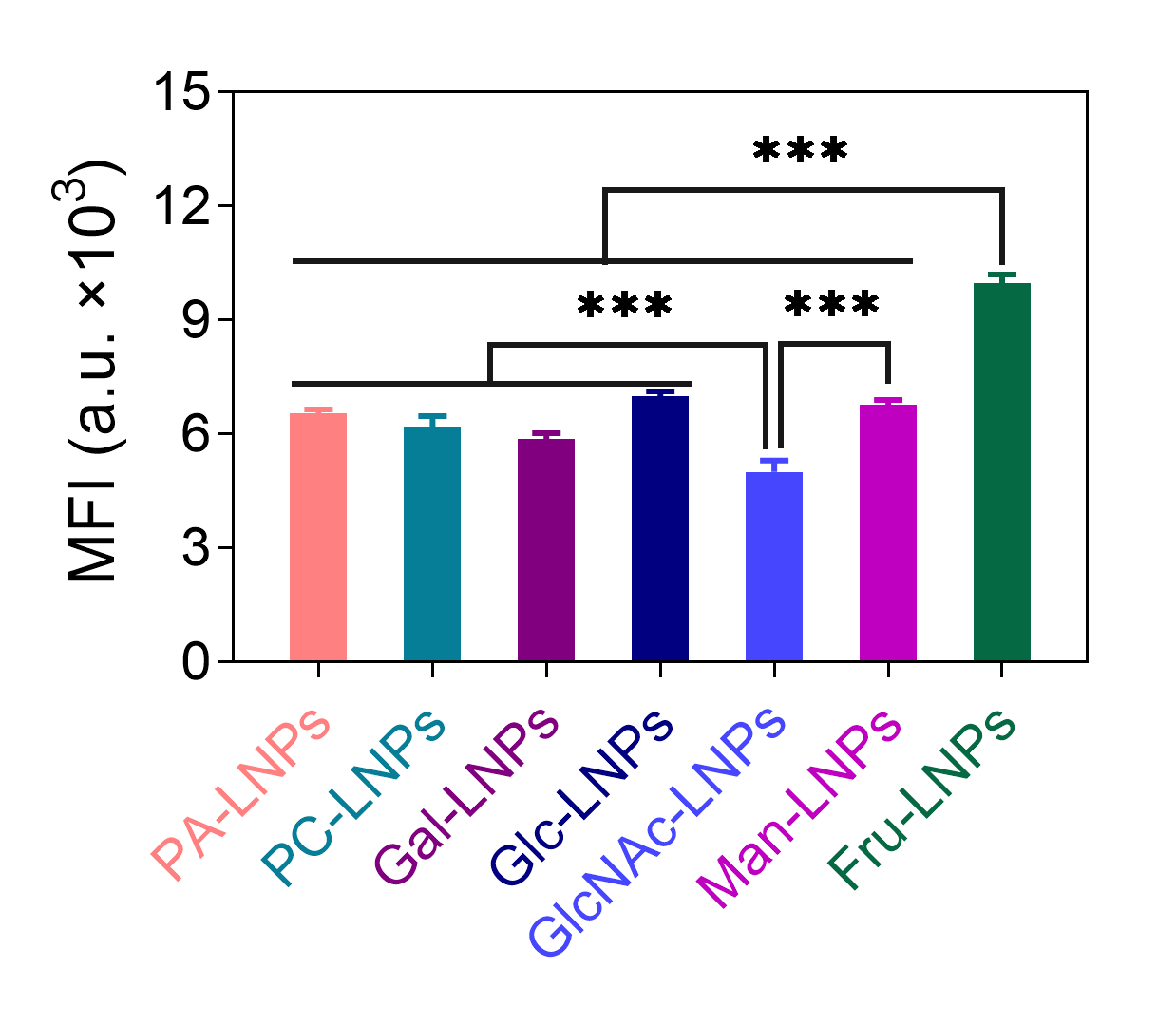
**

**Figure S36.** Uptake of breast cancer human serum (BCHS)-incubated G-LNPs by human-derived macrophage THP-1 cells. Data are presented as mean ± SD (*n* = 3).


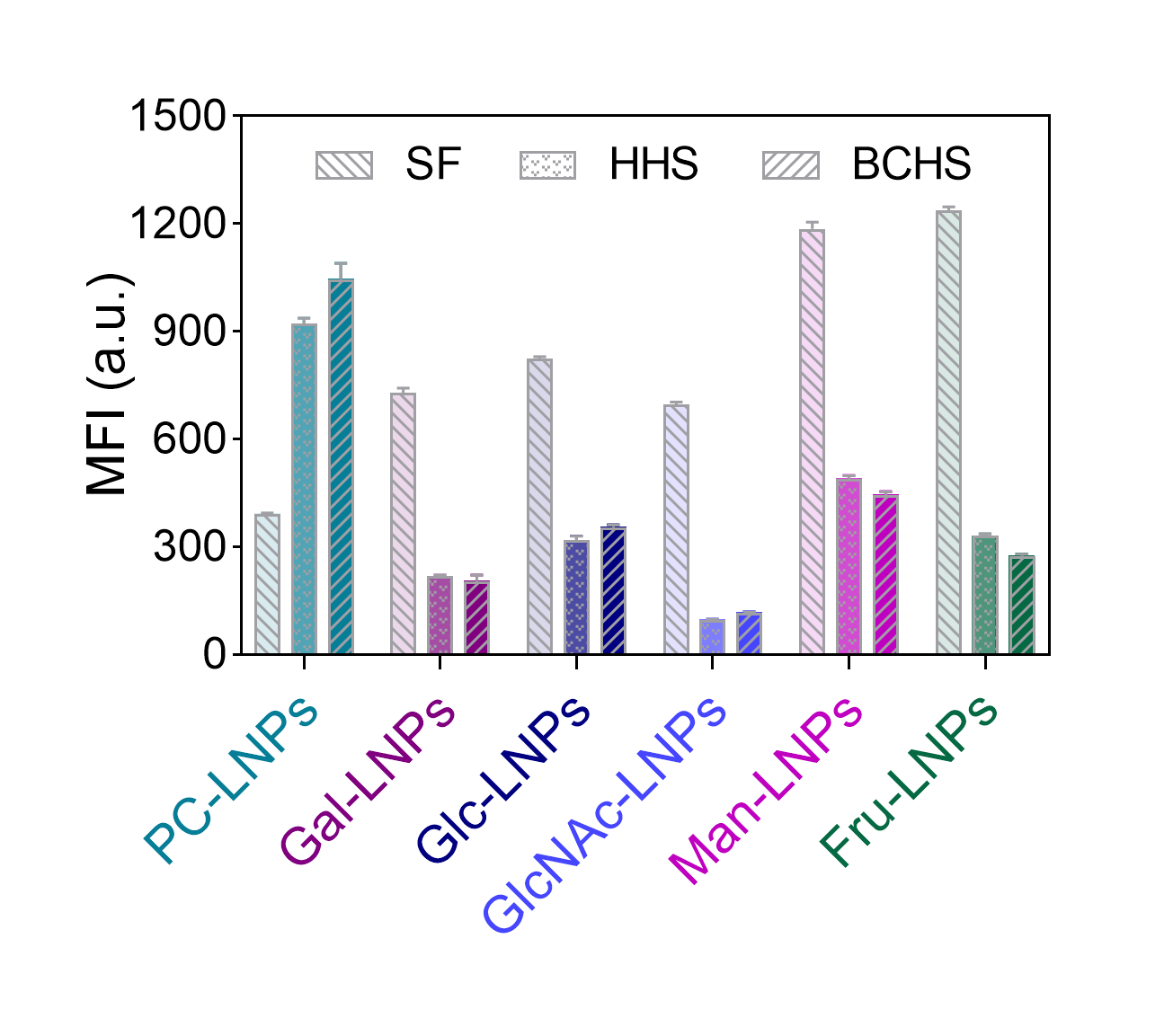


**Figure S37.** Uptake of mouse-derived macrophage RAW264.7 on G-LNPs incubated in human serum. Data are presented as mean ± SD (*n* = 3).


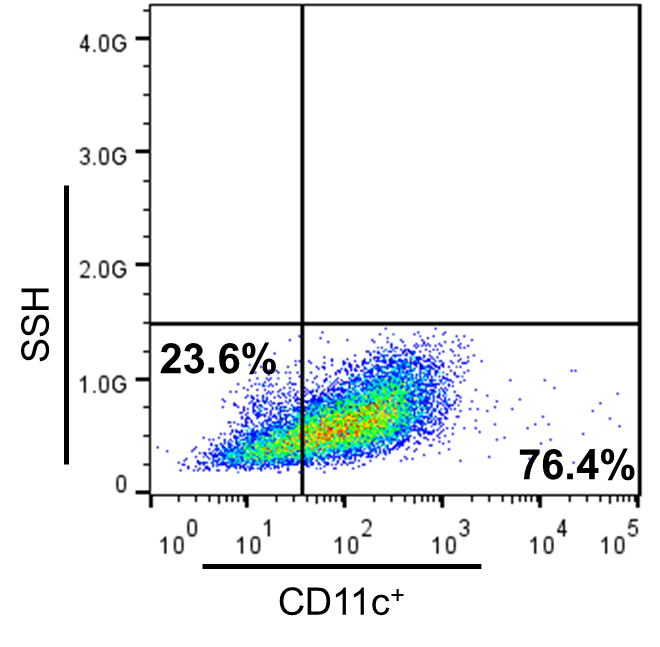


**Figure S38.** Ratio of CD11c+ cells in BMDCs. BMDCs were stained with anti-CD11c antibody and counted by flow cytometry.


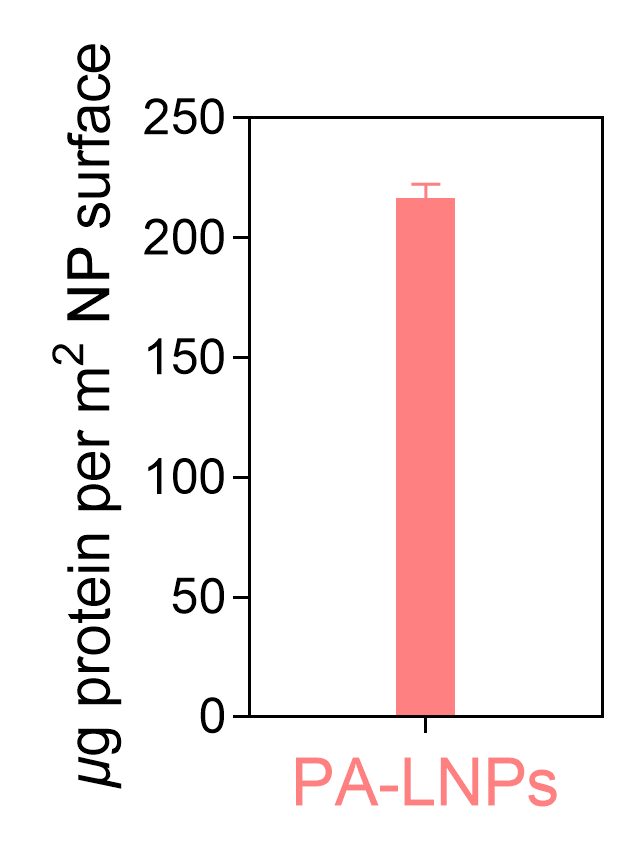


**Figure S39.** Quantification of adsorbed proteins on PA-LNPs (*μ*g protein per m^2^ NP surface) recovered from murine blood. Data are presented as mean ± SD (*n* = 3).


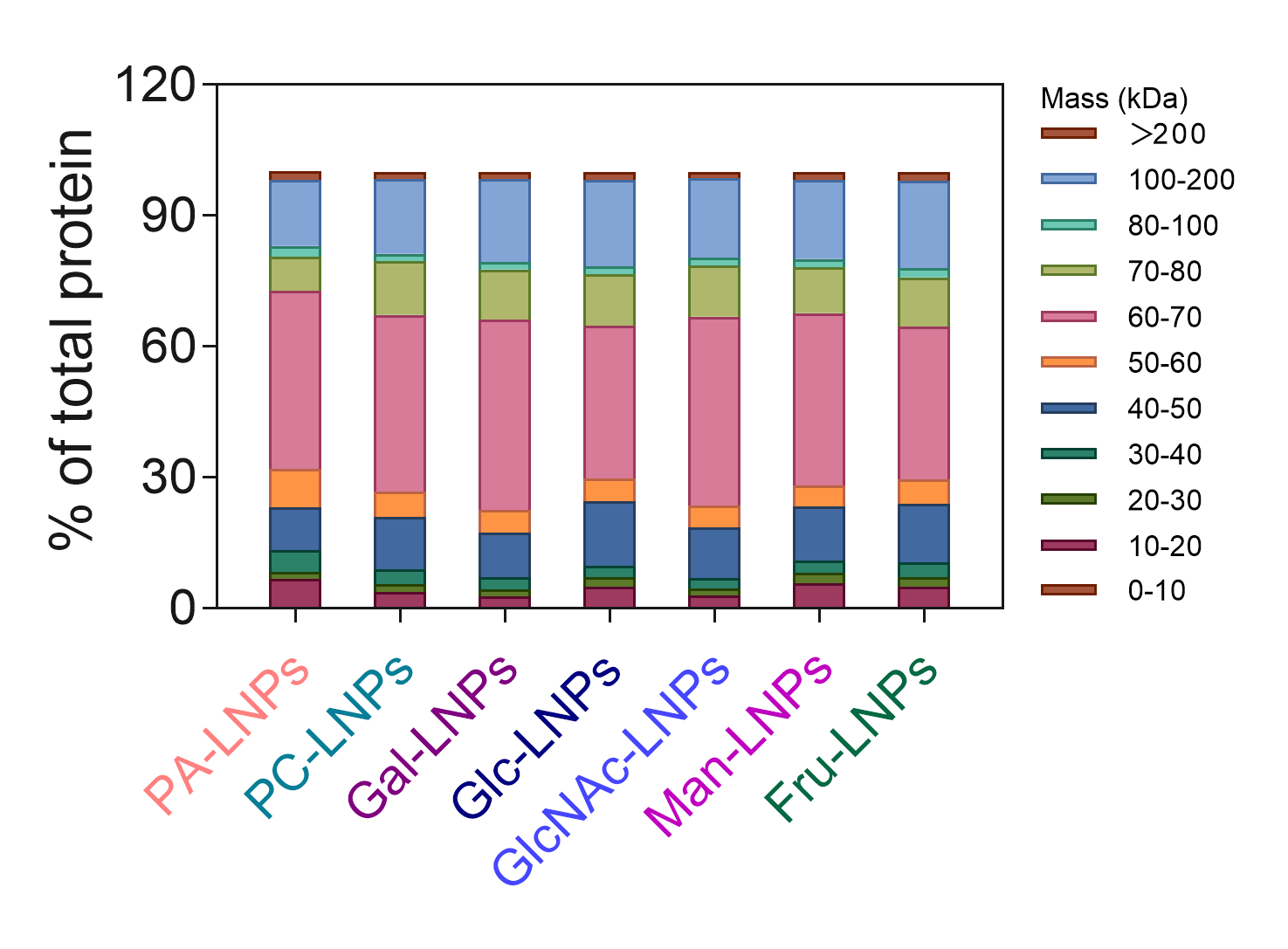


**Figure S40.** Classification of corona proteins according to molecular weight.


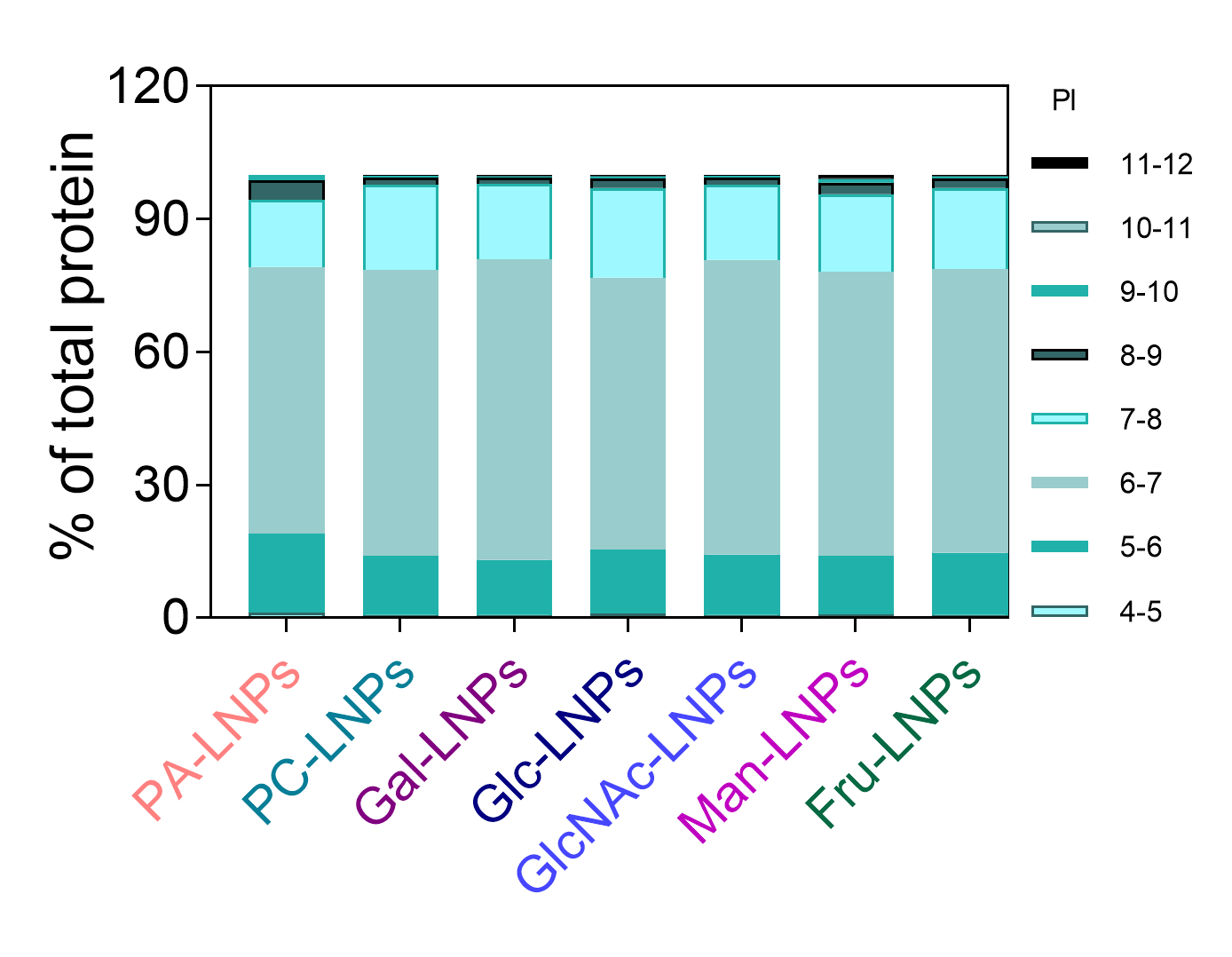


**Figure S41.** Classification of corona proteins according to calculated isoelectric point.


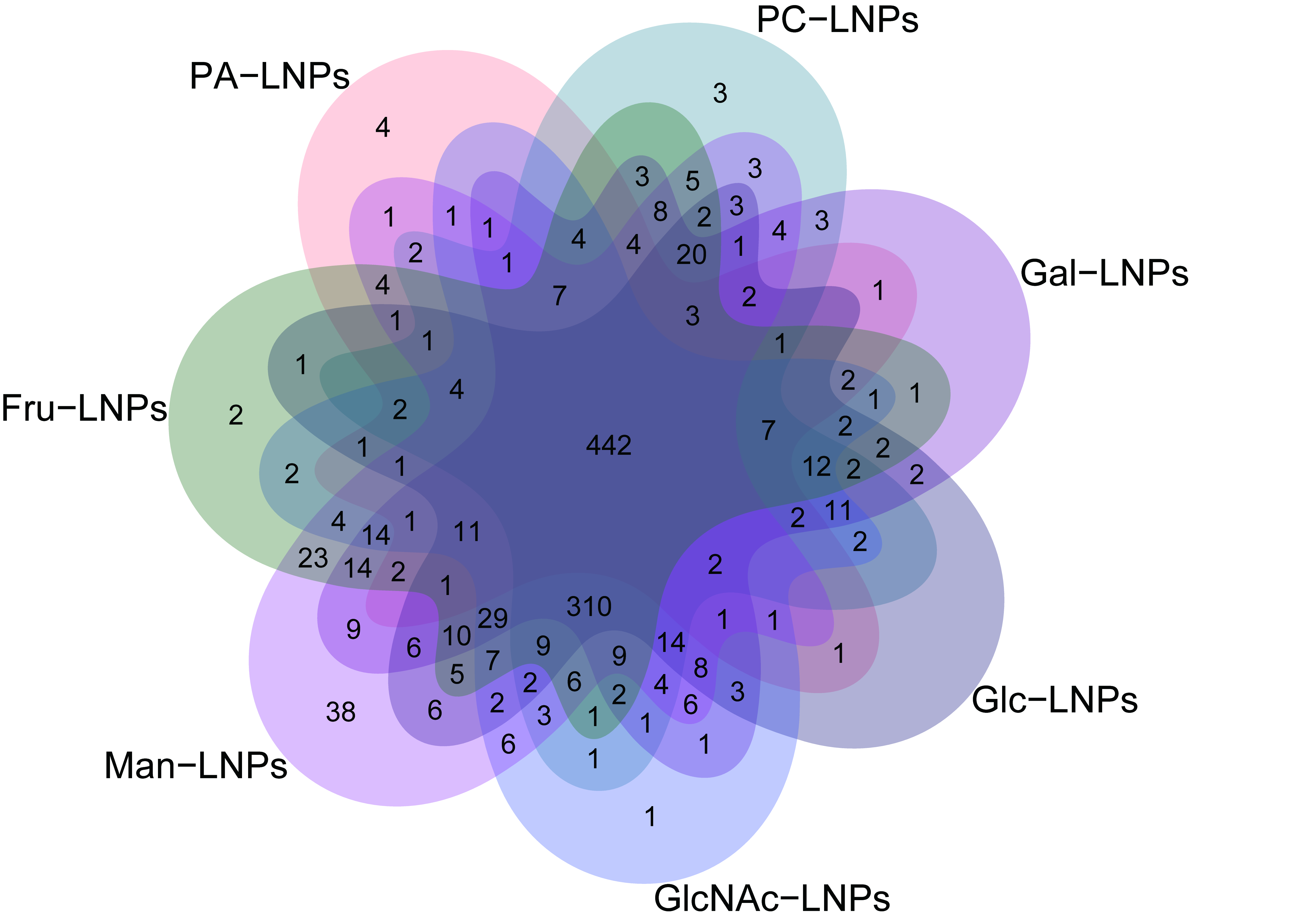


**Figure S42.** Venn diagram of the overlapping identified proteins in each G-LNP group.


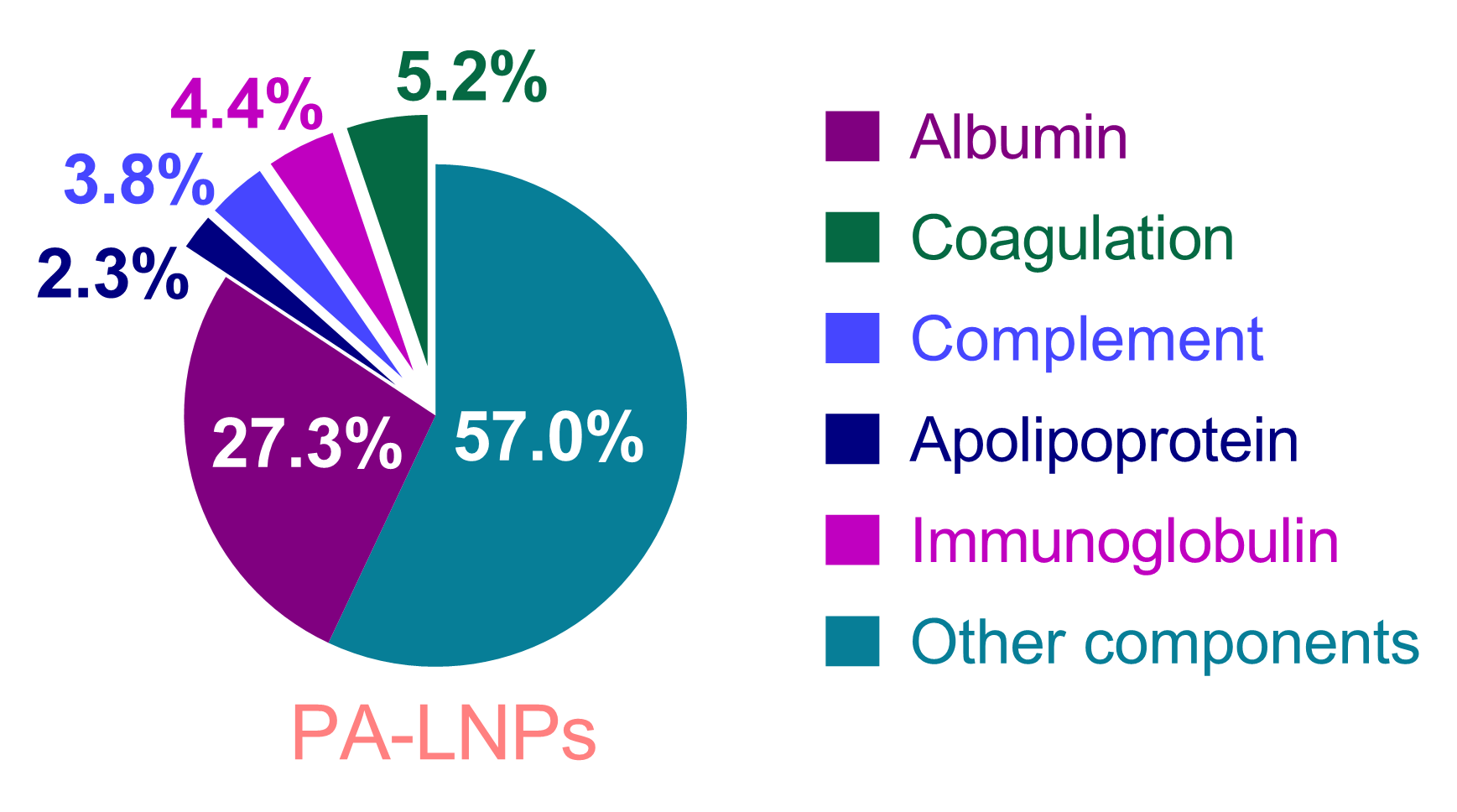


**Figure S43.** Functional classification of proteins adsorbed on the surface of PA-LNPs. All proteins identified by quantitative LC-MS/MS were categorized into six different classes based on their biological function.


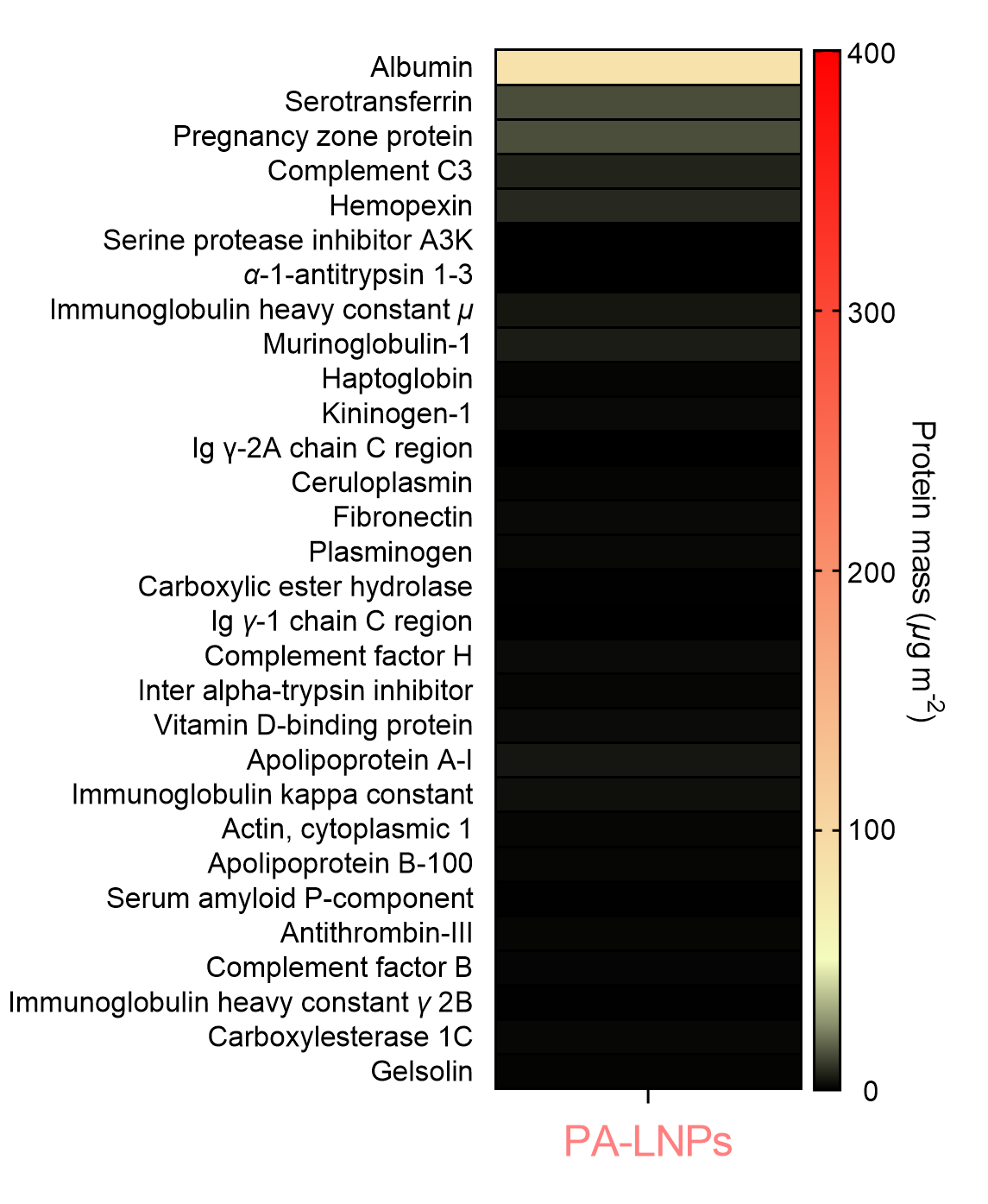


**Figure S44.** Heat map of the 30 most abundant proteins detected in the protein of PA-LNP-protein complex isolated from mice.


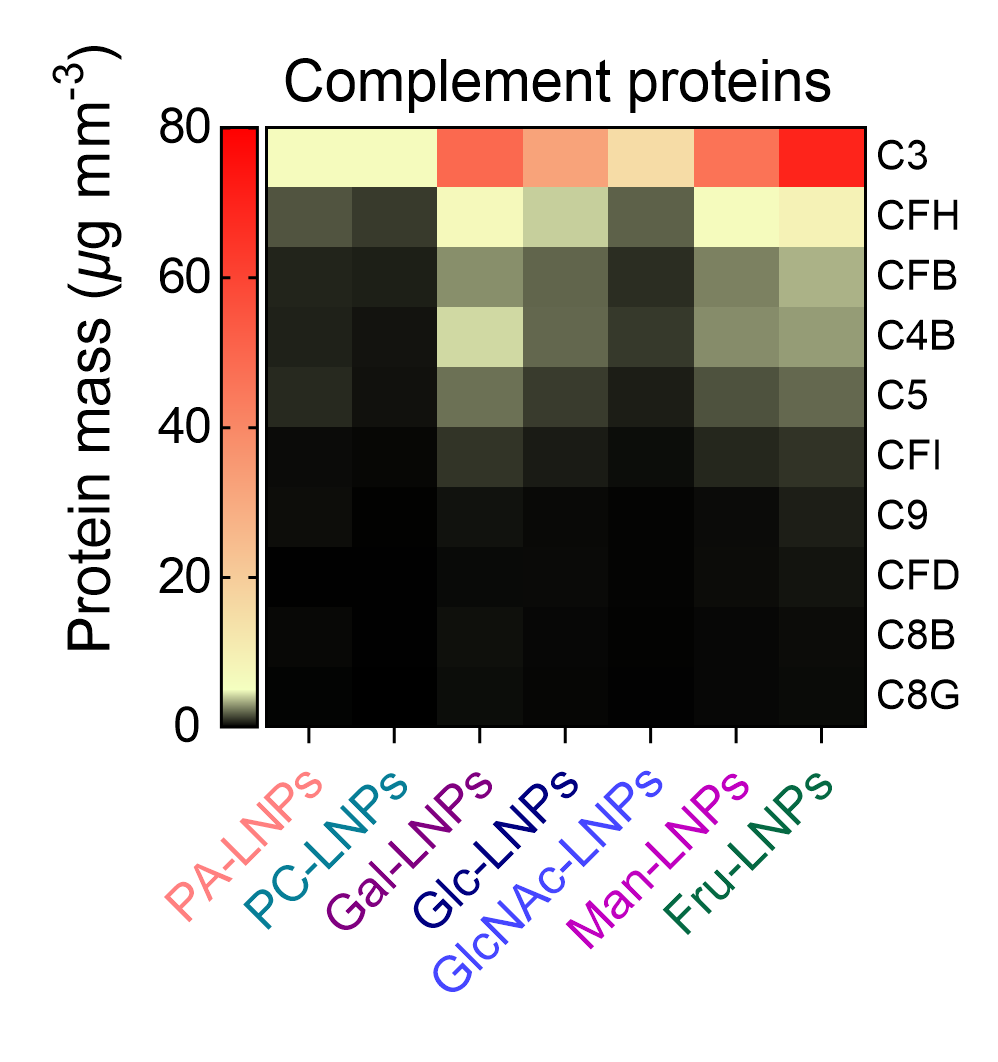


**Figure S45.** Relative abundance and classification of complement in the murine protein corona, as determined by LC-MS/MS.


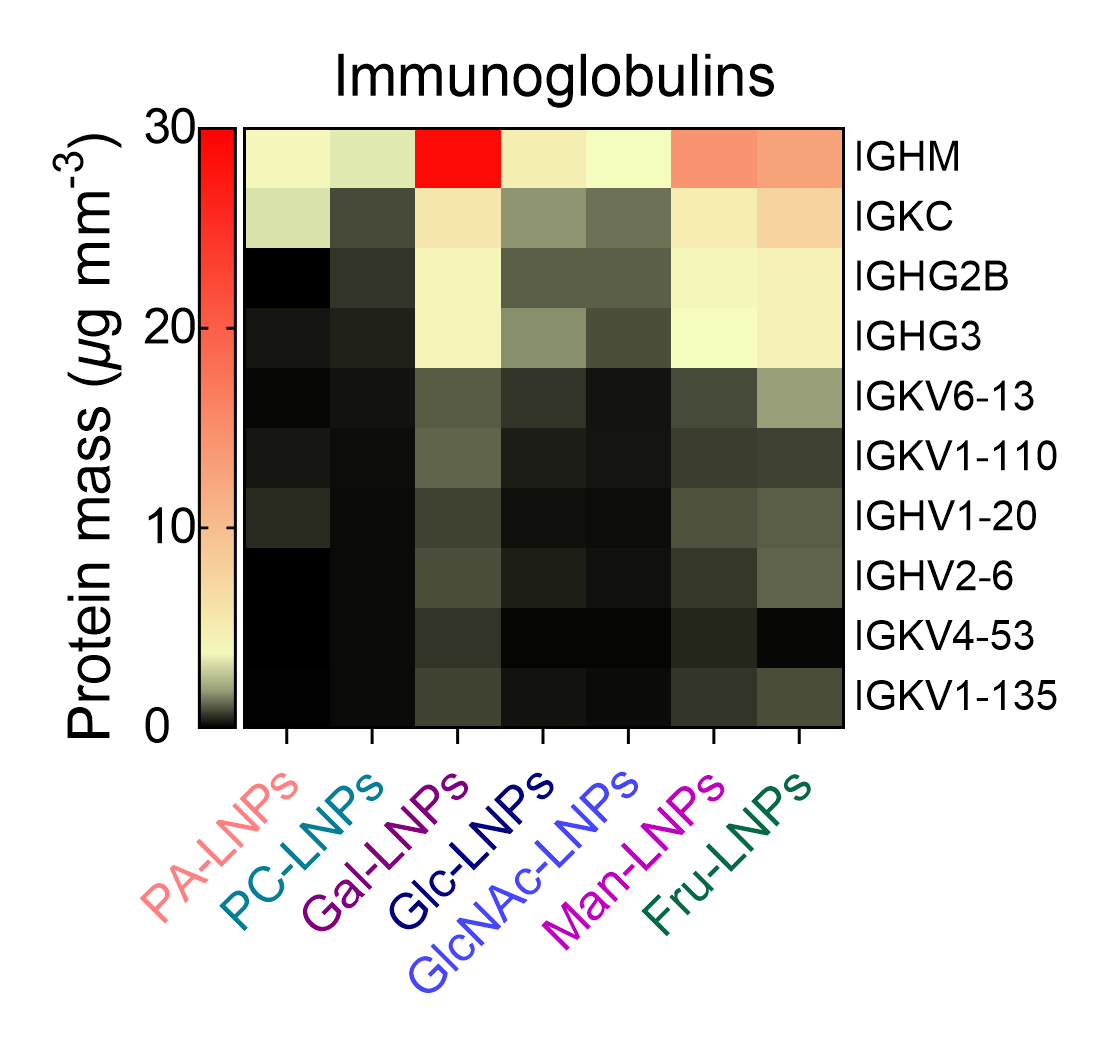


**Figure S46.** Relative abundance and classification of immunoglobulins in the murine protein corona, as determined by LC-MS/MS.


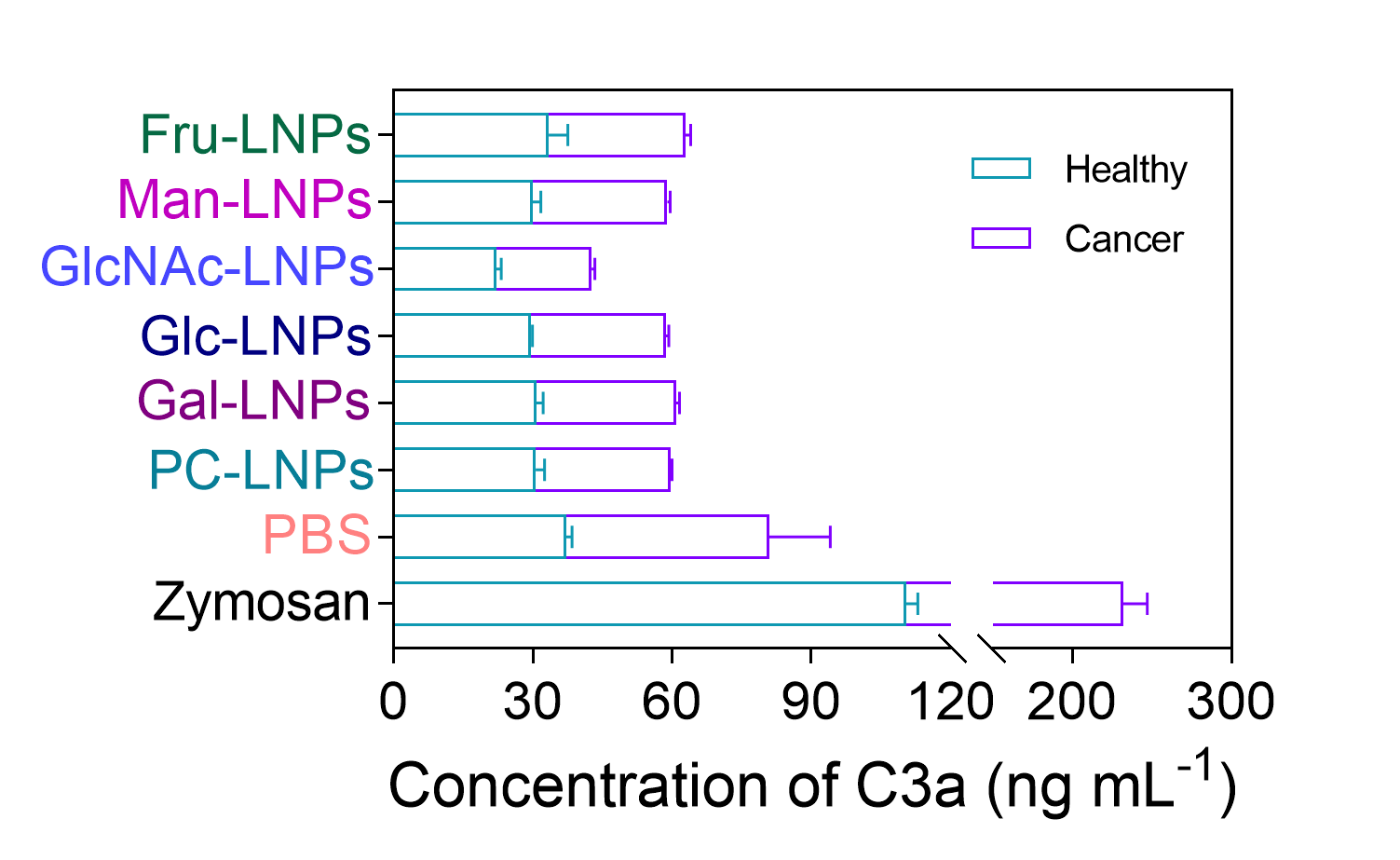


**Figure S47.** C3a concentration in healthy or cancer mice sera after incubation with G-LNPs. Data are presented as mean ± SD (*n* = 3).


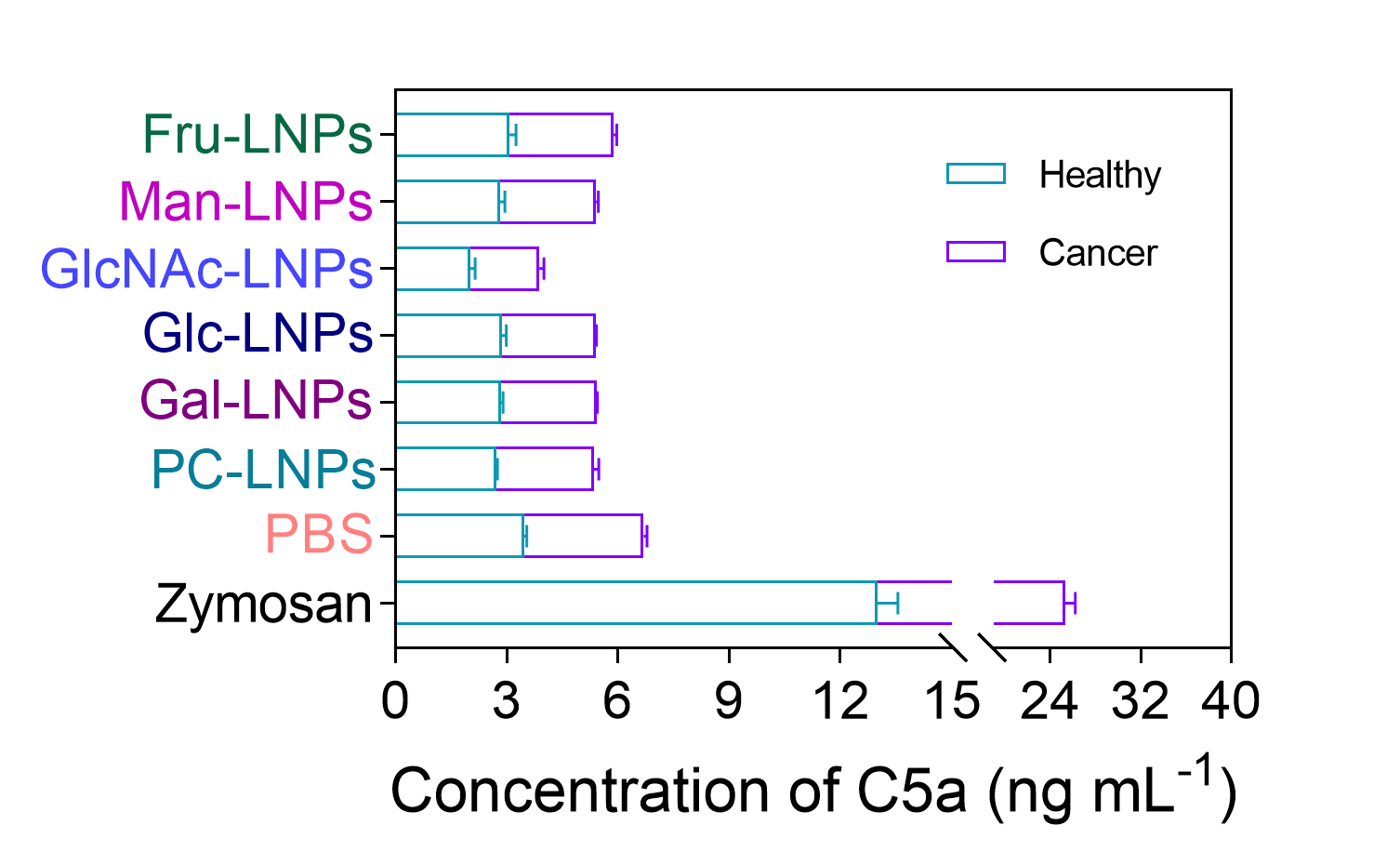


**Figure S48.** C5a concentration in healthy or cancer mice sera after incubation with G-LNPs. Data are presented as mean ± SD (*n* = 3).


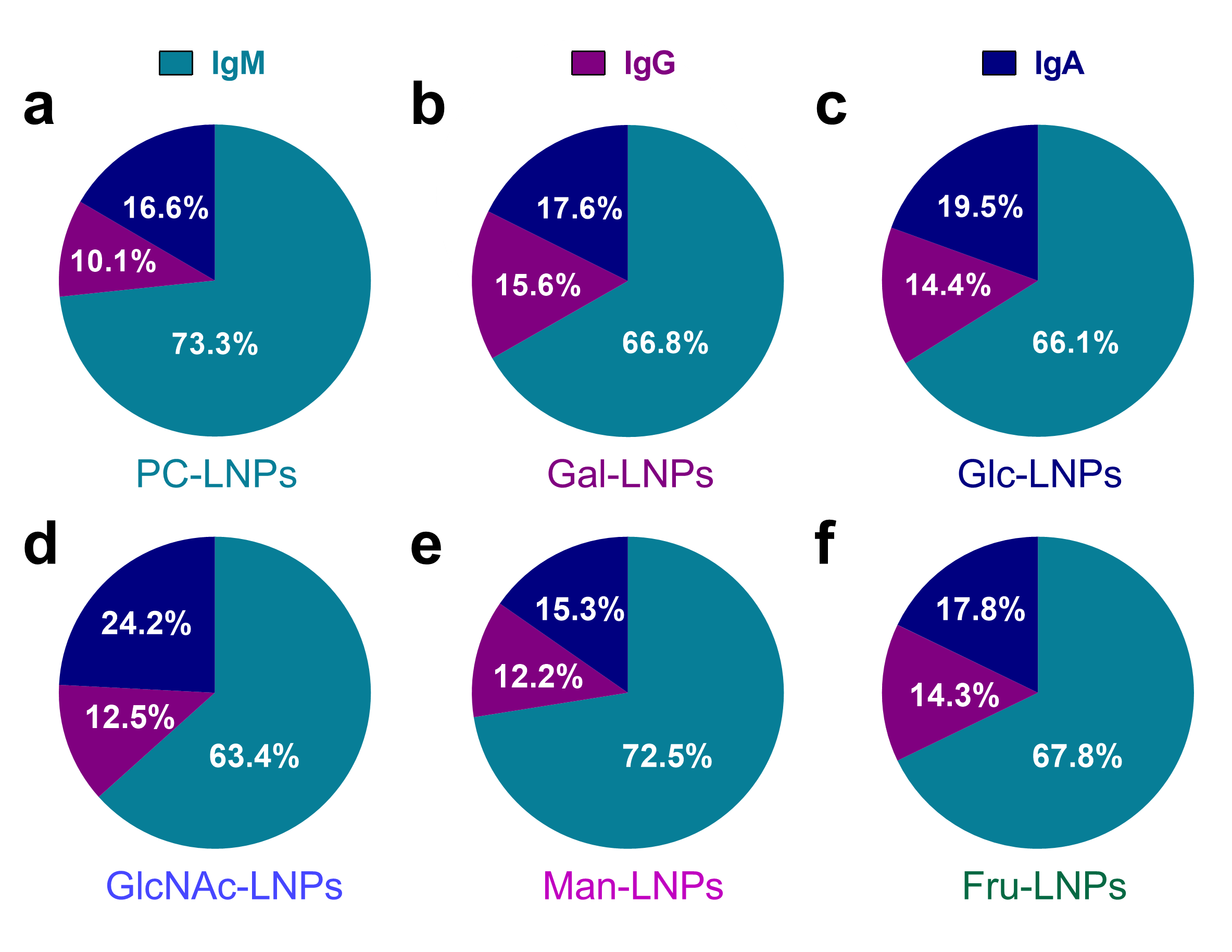


**Figure S49.** Relative amounts of IgG or IgM, and IgA among these 3 immunoglobulins in the protein corona of PC-LNPs (a), Gal-LNPs (b), Glc-LNPs (c), GlcNAc-LNPs (d), Man-LNPs (e), Fru-LNPs (f). Data are presented as mean ± SD (*n* = 3).


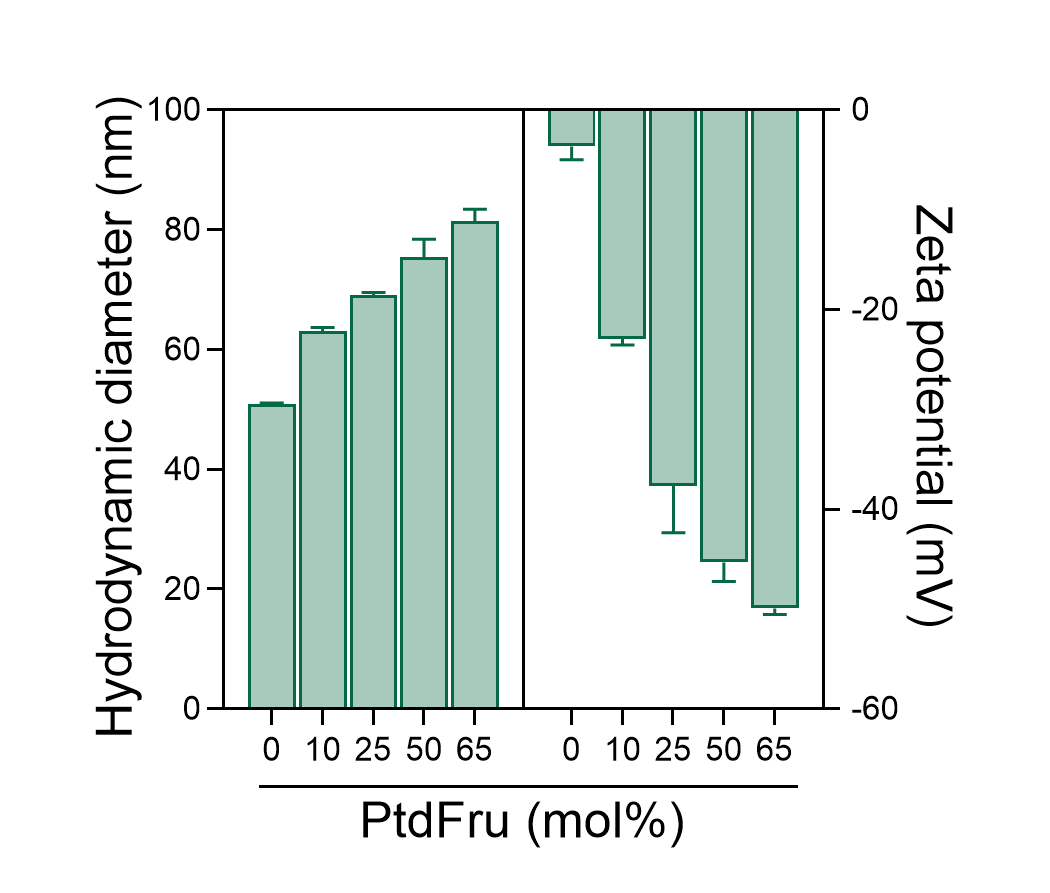


**Figure S50.** Hydrodynamic diameter and zeta potential of Fru-LNPs with varying molar ratios of PtdFru. Data are presented as mean ± SD (*n* = 3).


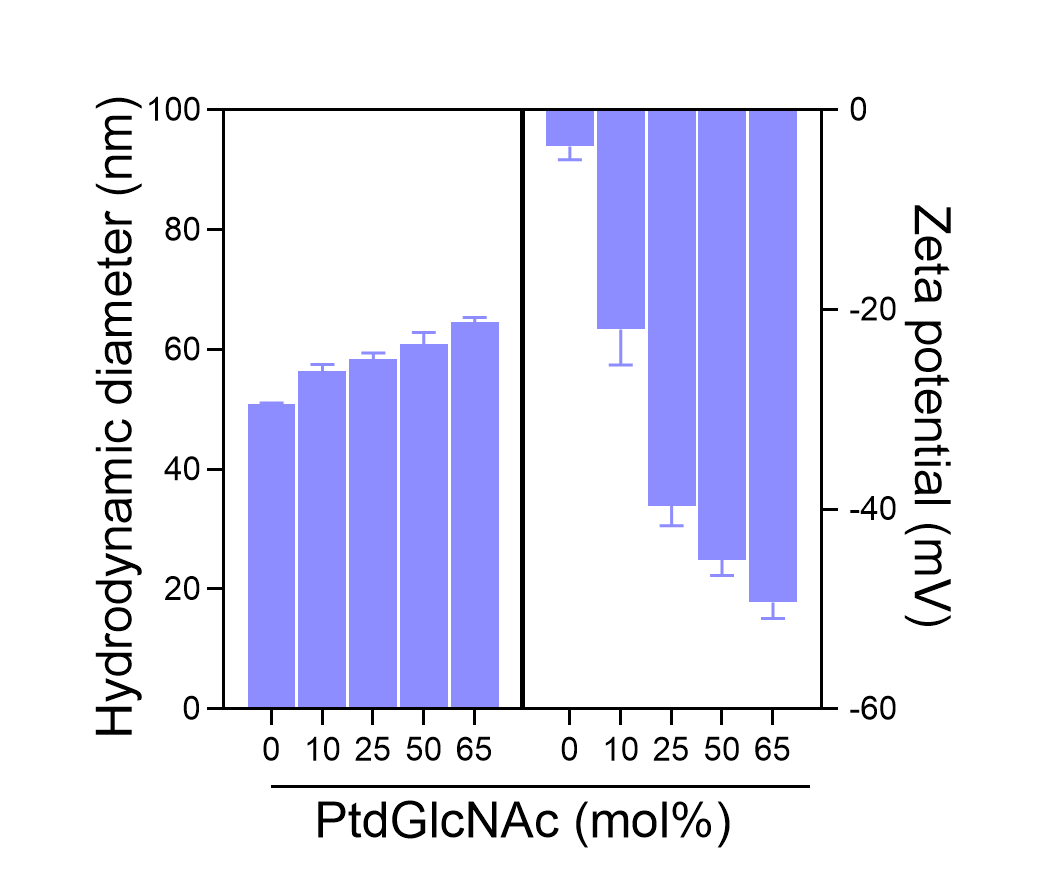


**Figure S51.** Hydrodynamic diameter and zeta potential of GlcNAc-LNPs with varying molar ratios of PtdGlcNAc. Data are presented as mean ± SD (*n* = 3).


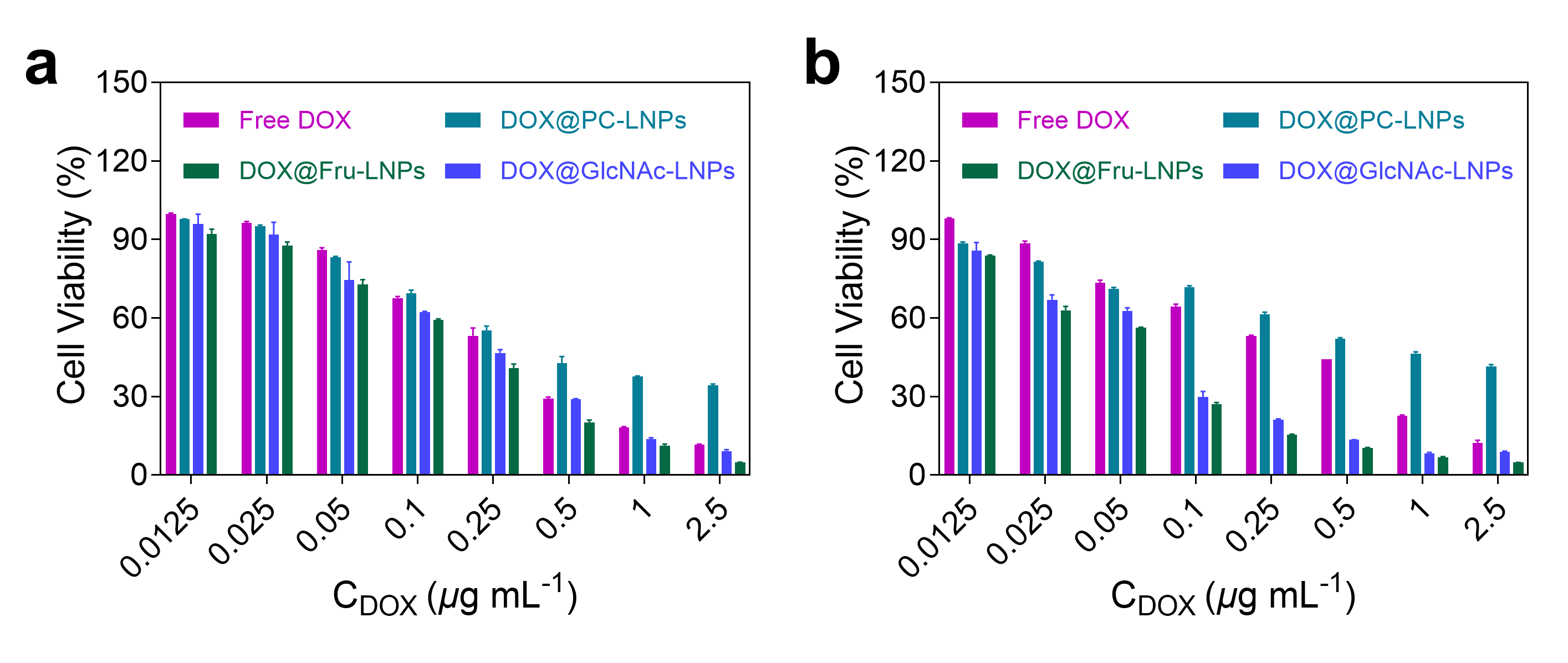


**Figure S52.** Cell viability of 4T1 cell (a) or MCF-7 cells (b) treated with different DOX@G-LNPs formulations at 48 h. Data are presented as mean ± SD (*n* = 3).


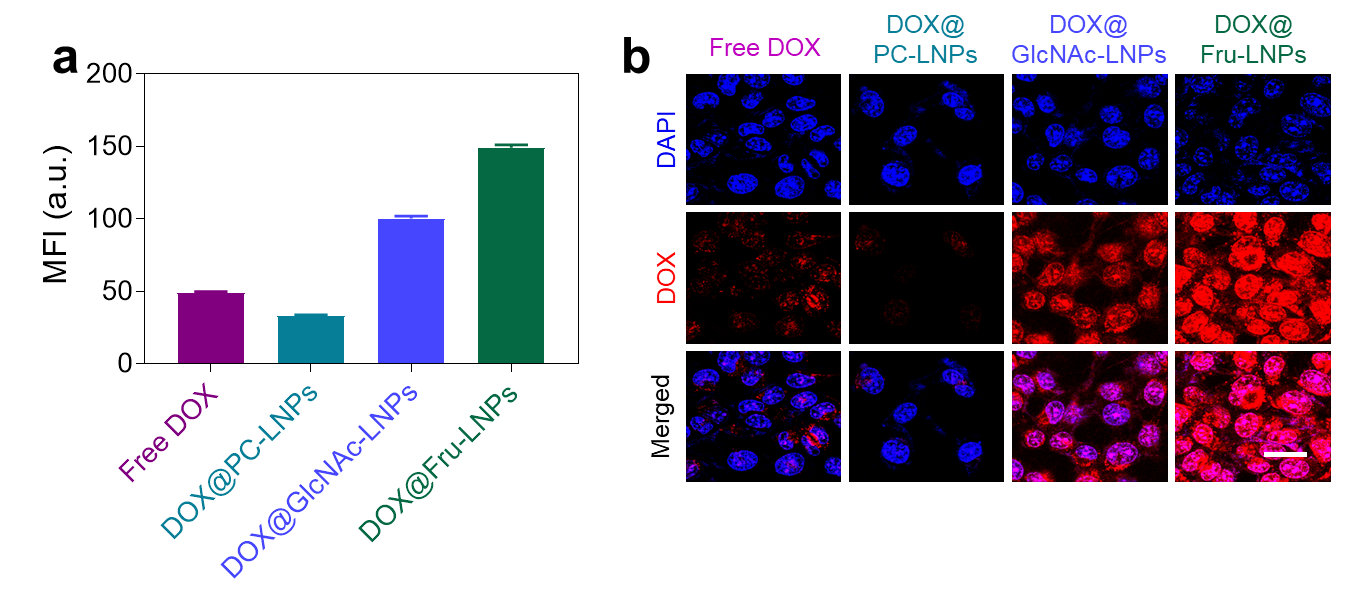


**Figure S53.** Flow cytometry analysis (a) and CLSM images (b) of DOX@G-LNPs uptake in 4T1 cells. Scale bars: 20 *μ*m. Data are presented as mean ± SD (*n* = 3).


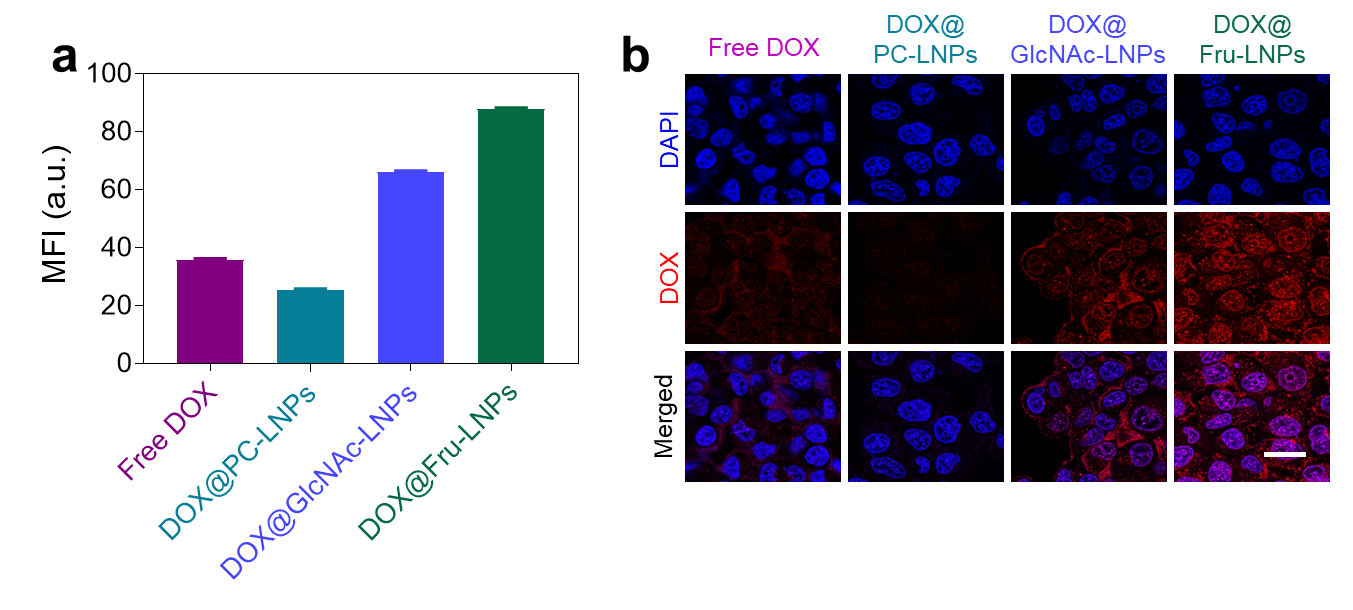


**Figure S54.** Flow cytometry analysis (a) and CLSM images (b) of DOX@G-LNPs uptake in MCF-7 cells. Scale bars: 20 *μ*m. Data are presented as mean ± SD (*n* = 3).


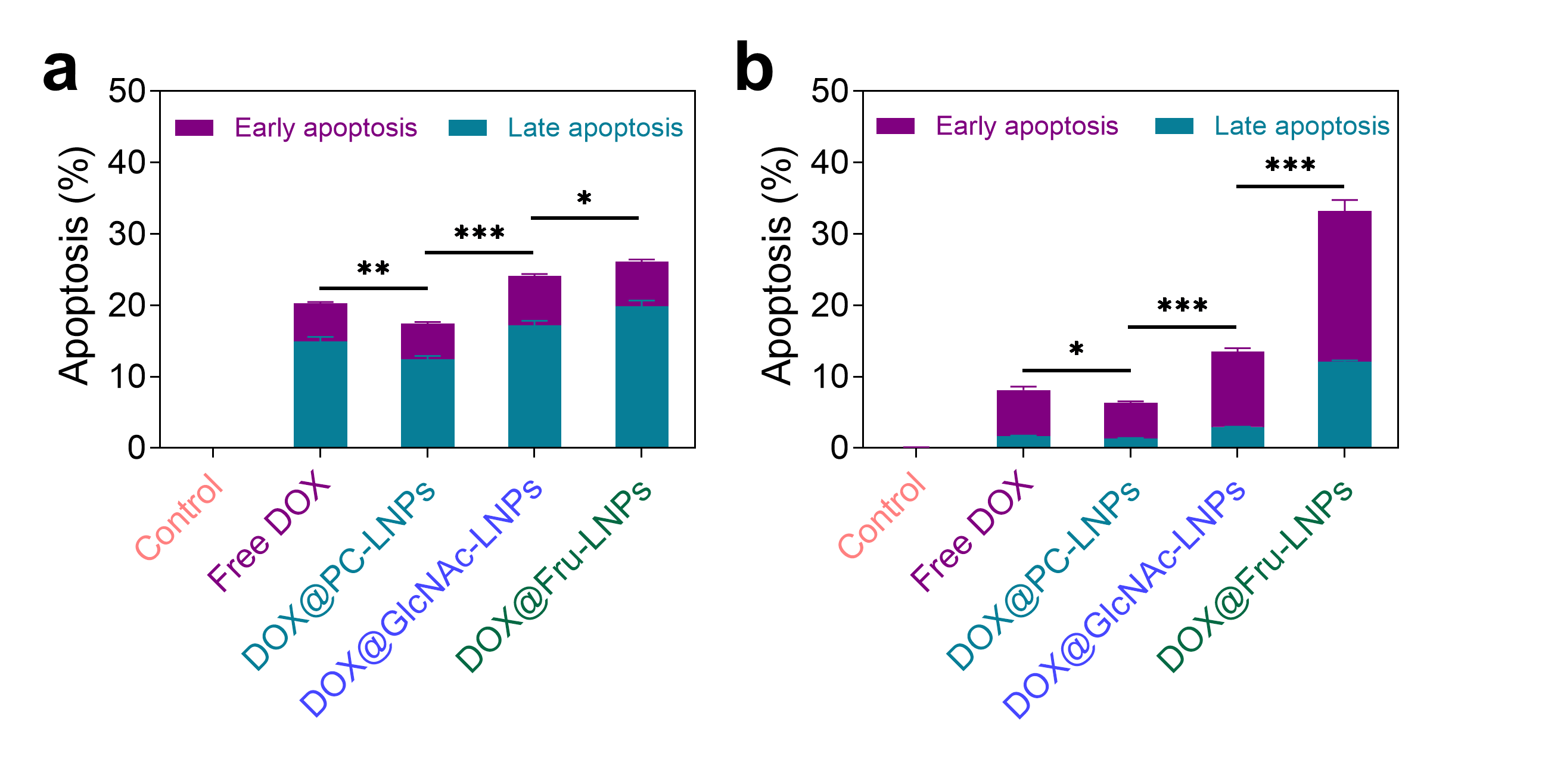


**Figure S55.** Cell apoptosis rate of 4T1 cells (a) and MCF-7 cells (b) treated with different DOX@G-LNPs formulations determined by flow cytometry. Data are presented as mean ± SD (*n* = 3).


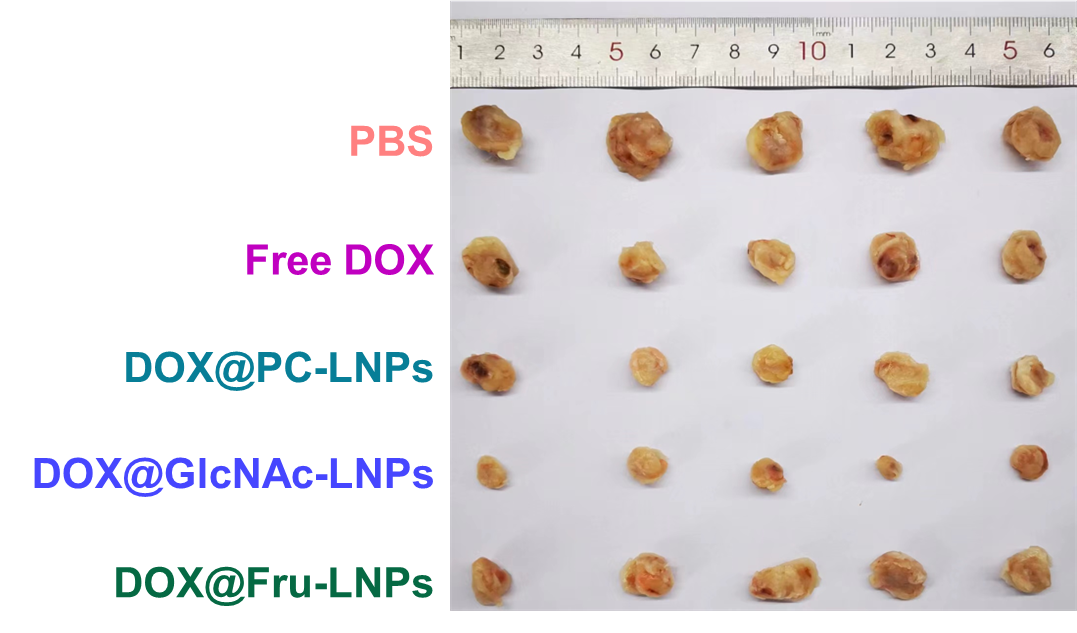


**Figure S56.** Photographs of tumors of each group after resection (*n* = 5).


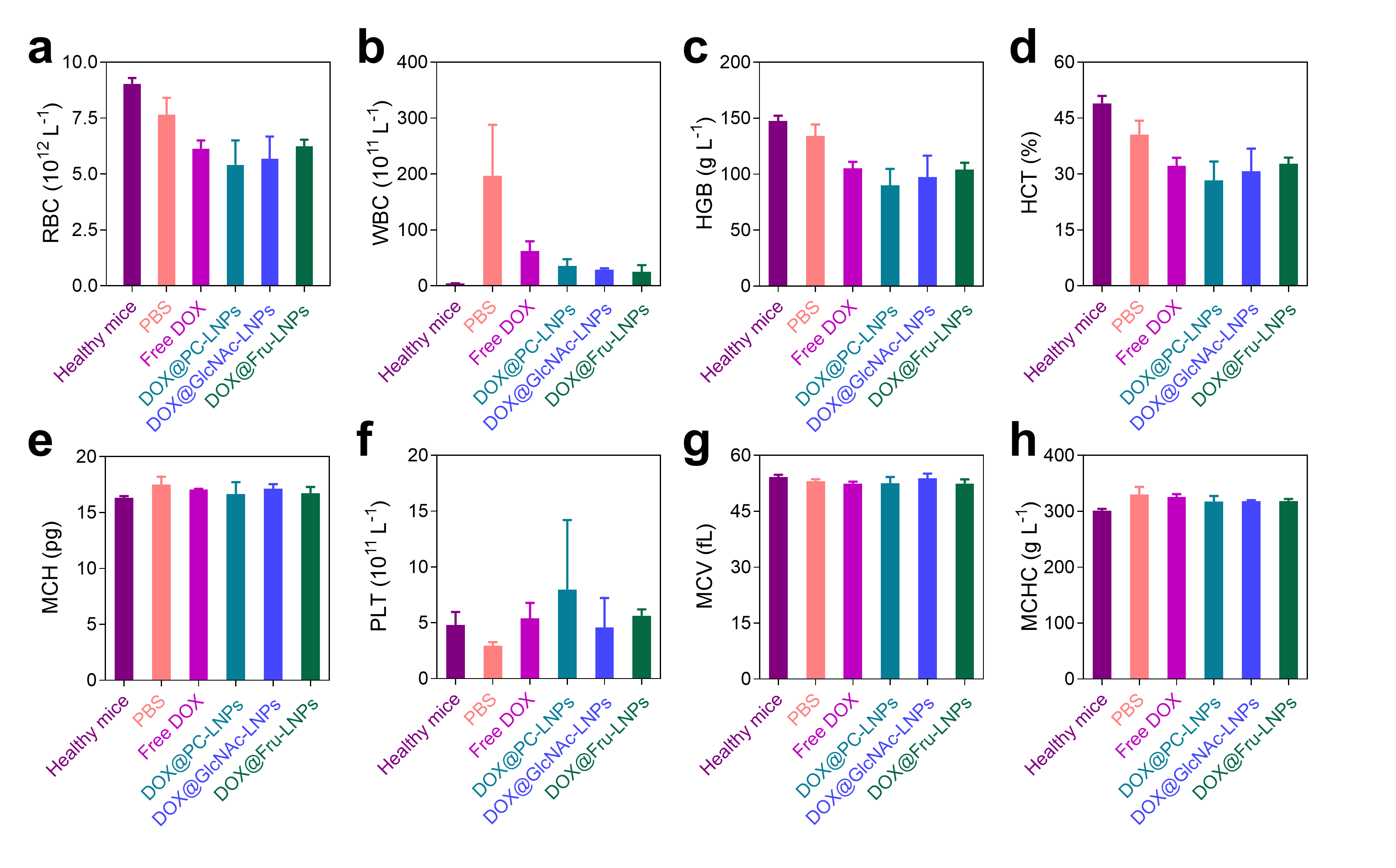


**Figure S57.** Blood routine analysis of mice after treatment with G-LNPs at 24 h post-injection. RBC (a), WBC (b), HGB (c), HCT (d), MCH (e), PLT (f), MCV (g), MCHC (h). Data are presented as mean ± SD (*n* = 3).


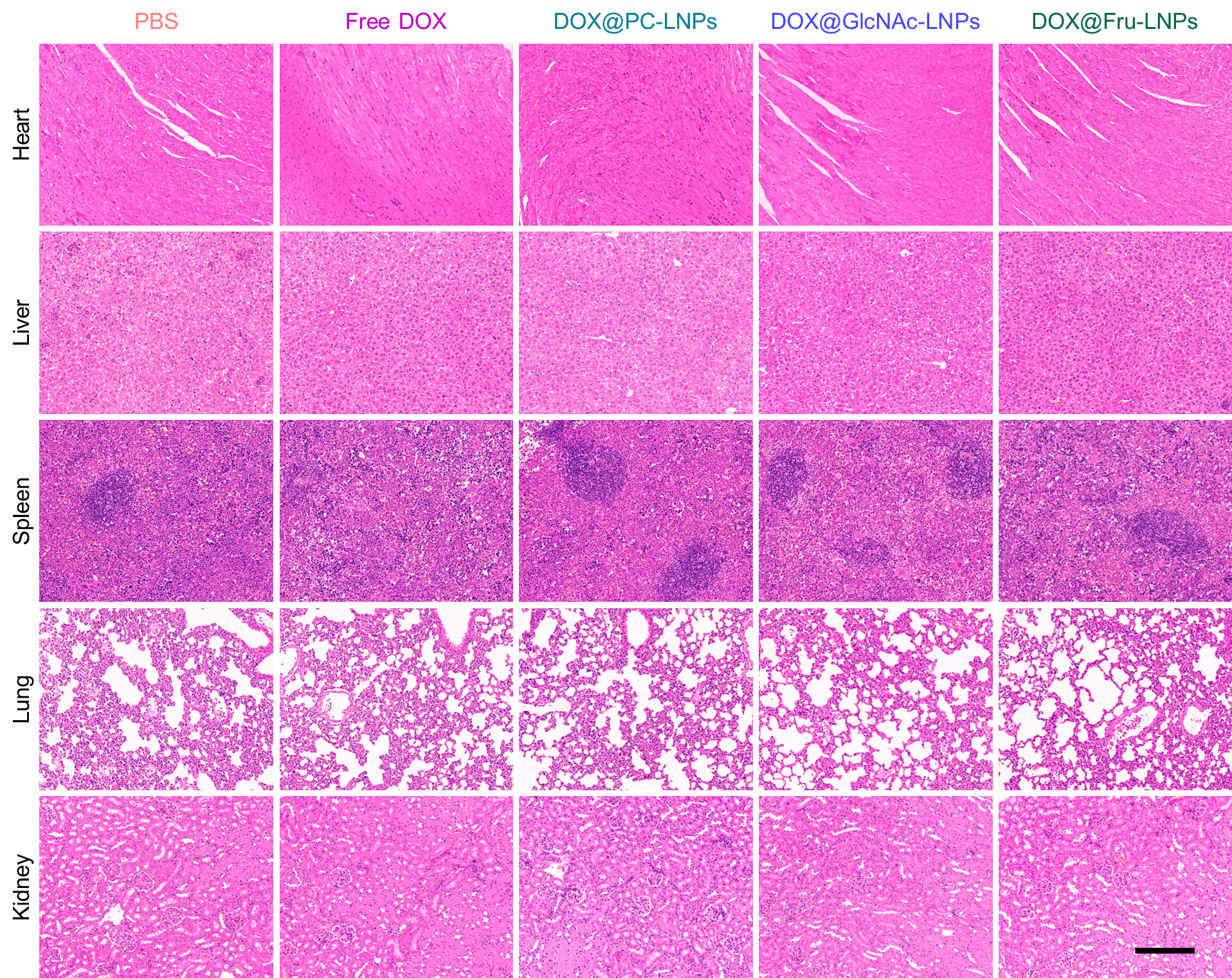


**Figure S58.** Optical microscopy images of pathological sections of major organs in G-LNPs. Scale bars: 200 *μ*m.

**Table S1**. Hydrodynamic diameter (*D*_h_) and zeta potential of liposomal nanoparticles.

| Sample name | *D*_h_ | PDI | Zeta potential (mV) |
| --- | --- | --- | --- |
| PA-LNPs | 70.0±2.3 | 0.175±0.012 | -45.1±2.5 |
| PC-LNPs | 54.7±1.8 | 0.235±0.004 | -3.6±1.1 |
| Gal-LNPs | 69.2±0.9 | 0.236±0.009 | -34.6±2.3 |
| Glc-LNPs | 69.0±3.4 | 0.247±0.010 | -41.5±2.7 |
| GlcNAc-LNPs | 60.9±1.6 | 0.239±0.015 | -45.1±1.3 |
| Man-LNPs | 69.0±1.0 | 0.255±0.004 | -43.0±3.1 |
| Fru-LNPs | 75.5±2.4 | 0.256±0.007 | -45.3±1.6 |

**Table S2**. Size distribution of liposomal nanoparticles by intensity.

| Sample name | Peak1 | | Peak2 | | Peak3 | |
| --- | --- | --- | --- | --- | --- | --- |
|  | Size (nm) | Intensity (%) | Size (nm) | Intensity (%) | Size (nm) | Intensity (%) |
| PA-LNPs-1 | 85.3 | 100.0 | 0.0 | 0.0 | 0.0 | 0.0 |
| PA-LNPs-2 | 94.5 | 100.0 | 0.0 | 0.0 | 0.0 | 0.0 |
| PA-LNPs-3 | 83.9 | 100.0 | 0.0 | 0.0 | 0.0 | 0.0 |
| PC-LNPs-1 | 78.5 | 100.0 | 0.0 | 0.0 | 0.0 | 0.0 |
| PC-LNPs-2 | 58.1 | 94.8 | 3866.0 | 5.2 | 0.0 | 0.0 |
| PC-LNPs-3 | 74.1 | 100.0 | 0.0 | 0.0 | 0.0 | 0.0 |
| Gal-LNPs-1 | 91.9 | 95.3 | 19.5 | 4.7 | 0.0 | 0.0 |
| Gal-LNPs-2 | 94.3 | 100.0 | 0.0 | 0.0 | 0.0 | 0.0 |
| Gal-LNPs-3 | 88.2 | 99.2 | 12.1 | 0.8 | 0.0 | 0.0 |
| Glc-LNPs-1 | 82.7 | 96.5 | 14.5 | 2.4 | 4799.0 | 1.1 |
| Glc-LNPs-2 | 86.0 | 100.0 | 0.0 | 0.0 | 0.0 | 0.0 |
| Glc-LNPs-3 | 80.6 | 100.0 | 0.0 | 0.0 | 0.0 | 0.0 |
| GlcNAc-LNPs-1 | 68.3 | 93.5 | 13.0 | 6.5 | 0.0 | 0.0 |
| GlcNAc-LNPs-2 | 61.8 | 98.5 | 5.3 | 1.5 | 0.0 | 0.0 |
| GlcNAc-LNPs-3 | 62.1 | 90.3 | 17.0 | 9.7 | 0.0 | 0.0 |
| Man-LNPs-1 | 97.2 | 99.1 | 10.5 | 0.9 | 0.0 | 0.0 |
| Man-LNPs-2 | 90.7 | 100.0 | 0.0 | 0.0 | 0.0 | 0.0 |
| Man-LNPs-3 | 91.5 | 100.0 | 0.0 | 0.0 | 0.0 | 0.0 |
| Fru-LNPs-1 | 100.1 | 92.0 | 25.1 | 8.0 | 0.0 | 0.0 |
| Fru-LNPs-2 | 98.3 | 96.2 | 17.6 | 3.8 | 0.0 | 0.0 |
| Fru-LNPs-3 | 90.7 | 100.0 | 0.0 | 0.0 | 0.0 | 0.0 |

**Table S3**. Size distribution of liposomal nanoparticles by number.

| Sample name | Peak1 | | Peak2 | | Peak3 | |
| --- | --- | --- | --- | --- | --- | --- |
|  | Size (nm) | Number (%) | Size (nm) | Number (%) | Size (nm) | Number (%) |
| PA-LNPs-1 | 33.7 | 100.0 | 0.0 | 0.0 | 0.0 | 0.0 |
| PA-LNPs-2 | 33.2 | 100.0 | 0.0 | 0.0 | 0.0 | 0.0 |
| PA-LNPs-3 | 25.4 | 100.0 | 0.0 | 0.0 | 0.0 | 0.0 |
| PC-LNPs-1 | 25.5 | 100.0 | 0.0 | 0.0 | 0.0 | 0.0 |
| PC-LNPs-2 | 28.2 | 100.0 | 0.0 | 0.0 | 0.0 | 0.0 |
| PC-LNPs-3 | 28.4 | 100.0 | 0.0 | 0.0 | 0.0 | 0.0 |
| Gal-LNPs-1 | 16.6 | 100.0 | 0.0 | 0.0 | 0.0 | 0.0 |
| Gal-LNPs-2 | 22.9 | 100.0 | 0.0 | 0.0 | 0.0 | 0.0 |
| Gal-LNPs-3 | 10.8 | 100.0 | 0.0 | 0.0 | 0.0 | 0.0 |
| Glc-LNPs-1 | 12.8 | 100.0 | 0.0 | 0.0 | 0.0 | 0.0 |
| Glc-LNPs-2 | 18.4 | 100.0 | 0.0 | 0.0 | 0.0 | 0.0 |
| Glc-LNPs-3 | 45.9 | 100.0 | 0.0 | 0.0 | 0.0 | 0.0 |
| GlcNAc-LNPs-1 | 38.3 | 0.3 | 10.6 | 99.7 | 0.0 | 0.0 |
| GlcNAc-LNPs-2 | 15.3 | 0.2 | 4.5 | 99.8 | 0.0 | 0.0 |
| GlcNAc-LNPs-3 | 13.3 | 100.0 | 0.0 | 0.0 | 0.0 | 0.0 |
| Man-LNPs-1 | 8.7 | 100.0 | 0.0 | 0.0 | 0.0 | 0.0 |
| Man-LNPs-2 | 17.7 | 100.0 | 0.0 | 0.0 | 0.0 | 0.0 |
| Man-LNPs-3 | 14.2 | 100.0 | 0.0 | 0.0 | 0.0 | 0.0 |
| Fru-LNPs-1 | 20.5 | 100.0 | 0.0 | 0.0 | 0.0 | 0.0 |
| Fru-LNPs-2 | 13.1 | 100.0 | 0.0 | 0.0 | 0.0 | 0.0 |
| Fru-LNPs-3 | 11.4 | 100.0 | 0.0 | 0.0 | 0.0 | 0.0 |

**Table S4**. Encapsulation efficiency (EE) of DOX@G-LNPs

| Sample name | DOX@PC-LNPs | DOX@Fru-LNPs | DOX@GlcNAc-LNPs |
| --- | --- | --- | --- |
| EE (%) | 92.13%±1.17% | 95.43%±0.61% | 95.43%±4.85% |

**References**

[1] G. Haran, R. Cohen, L. K. Bar, Y. Barenholz, *Biochim Biophys Acta* **1993**, *1151*, 201-215.

[2] X. Wei, J. Gao, C. Zhan, C. Xie, Z. Chai, D. Ran, M. Ying, P. Zheng, W. Lu, *J Control Release* **2015**, *218*, 13-21.

[3] J. Guan, Q. Shen, Z. Zhang, Z. Jiang, Y. Yang, M. Lou, J. Qian, W. Lu, C. Zhan, *Nat Commun* **2018**, *9*, 2982.

[4] D. Kim, C. H. Whang, J. Hong, M. C. Prayogo, W. Jung, S. Lee, H. Shin, Y. Kim, J. Yu, M. J. Kim, K. Kim, H. S. Lee, S. Jon, *Adv Mater* **2024**, *36*, e2311283.
